# Novel Algorithms for Improved Sensitivity in Non-Invasive Prenatal Testing

**DOI:** 10.1038/s41598-017-02031-5

**Published:** 2017-05-12

**Authors:** L. F. Johansson, E. N. de Boer, H. A. de Weerd, F. van Dijk, M. G. Elferink, G. H. Schuring-Blom, R. F. Suijkerbuijk, R. J. Sinke, G. J. te Meerman, R. H. Sijmons, M. A. Swertz, B. Sikkema-Raddatz

**Affiliations:** 1 University of Groningen, University Medical Centre Groningen, Department of Genetics, Groningen, The Netherlands; 2 University of Groningen, University Medical Centre Groningen, Genomics Coordination Centre, Groningen, The Netherlands; 3 University Medical Centre Utrecht, Department of Genetics, Utrecht, The Netherlands

## Abstract

Non-invasive prenatal testing (NIPT) of cell-free DNA in maternal plasma, which is a mixture of maternal DNA and a low percentage of fetal DNA, can detect fetal aneuploidies using massively parallel sequencing. Because of the low percentage of fetal DNA, methods with high sensitivity and precision are required. However, sequencing variation lowers sensitivity and hampers detection of trisomy samples. Therefore, we have developed three algorithms to improve sensitivity and specificity: the chi-squared-based variation reduction (χ^2^VR), the regression-based Z-score (RBZ) and the Match QC score. The χ^2^VR reduces variability in sequence read counts per chromosome between samples, the RBZ allows for more precise trisomy prediction, and the Match QC score shows if the control group used is representative for a specific sample. We compared the performance of χ^2^VR to that of existing variation reduction algorithms (peak and GC correction) and that of RBZ to trisomy prediction algorithms (standard Z-score, normalized chromosome value and median-absolute-deviation-based Z-score). χ^2^VR and the RBZ both reduce variability more than existing methods, and thereby increase the sensitivity of the NIPT analysis. We found the optimal combination of algorithms was to use both GC correction and χ^2^VR for pre-processing and to use RBZ as the trisomy prediction method.

## Introduction

The discovery of cell-free fetal DNA (cffDNA) fragments in the maternal bloodstream^1^ in combination with the development of massively parallel sequencing has made it possible to perform non-invasive prenatal testing (NIPT). The traditional invasive procedures for prenatal aneuploidy testing, amniocentesis and chorionic villi biopsy, are associated with an elevated miscarriage risk^2^. This disadvantage can be overcome by NIPT, which can detect fetal aneuploidies in maternal blood as early as ten weeks into the pregnancy without the need for an invasive procedure^3^. NIPT makes use of cell-free DNA fragments isolated from blood plasma. Some of these fragments, the cffDNA, originate from the placenta and are informative of the fetus: when a chromosomal trisomy is present, the number of fragments originating from that chromosome will be higher than what is expected based upon statistical analysis using a set of non-trisomy control samples. Because NIPT is based upon analysis of very small amounts of DNA, measurements are very sensitive to the introduction of variability between samples and experiments. The statistical analysis in NIPT was first improved by the introduction of the Z-score calculation^4^, which compares the individual sample with a set of non-trisomy controls. However, when applying the standard Z-score calculation without prior data correction, a high variability was found for chromosomes 13 and 18^5^. This is undesirable because it lowers the sensitivity of the test. Thus, if a low fraction of cffDNA is present, there is a risk of false-negative results.

An important cause of variability is the guanine and cytosine (GC) content of the DNA fragments analyzed. There are various GC-bias correction methods, such as those based on locally weighted scatterplot smoothing regression (LOESS)^5–8^ or on the average coverage of genomic regions having a similar GC-content^9^. We used the latter method in combination with a peak correction that removes regions having significantly more reads than average^9^.

Variability can also be reduced by adapting the Z-score calculation, for instance by using the normalized chromosome value (NCV)^6, 10^ or the median absolute deviation (MAD) based Z-score^11^.

Our aim here was to further decrease variability and thus increase the sensitivity of NIPT. We therefore developed three new algorithms: the chi-squared-based variation reduction (χ^2^VR), the regression-based Z-score (RBZ), and the Match QC score. The χ^2^VR reduces the weight of the number of reads in regions that have a higher variation than expected by chance, regardless of the origin of the bias. The RBZ uses a model based on forward regression for prediction. The Match QC score calculates whether the non-trisomy control set is representative for the analyzed sample.

We compared the performance of our algorithms against and in combination with existing algorithms. Furthermore, we show that the Match QC score can indicate whether a sample fits within a control set.

## Material and Methods

**Figure 1:**
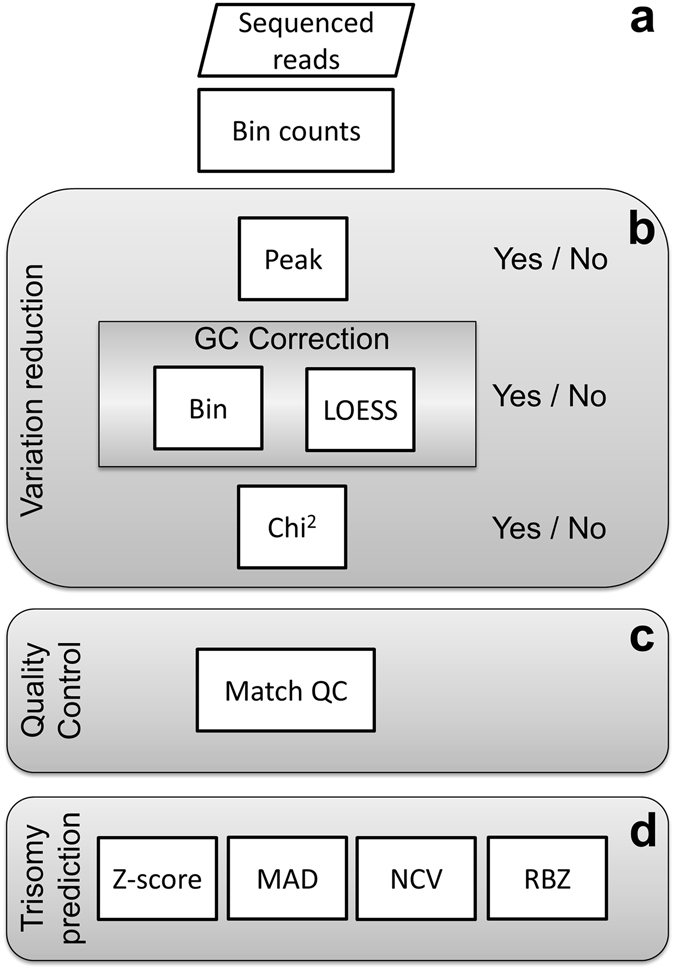
Flowchart showing the analysis steps. (**a**) First, sequenced reads are aligned, partitioned into 50,000 bp bins and counted. These bins are the units for further analysis and data quality can be improved using zero or more variation reduction methods. (**b**) Peak correction removes bins showing an unusually high coverage compared with the average coverage of bins on the same chromosome. GC correction corrects for coverage differences between bins having a different GC percentage, using one of two methods: ‘bin’ or ‘LOESS’ GC-correction. The chi-squared variation reduction corrects bins showing a higher variation in read counts between samples than expected by chance. Analysis is performed based on (corrected) read counts. (**c**) The Match QC indicates whether a control-group is informative for the analyzed sample. (**d**) Various algorithms (standard Z-score, MAD-based Z-score, Normalized Chromosome Value and Regression-based Z-score) are used for predicting trisomy.

To assess the added value of the χ^2^VR, RBZ and the Match QC score to the sensitivity and quality control of trisomy prediction, the performance of the algorithms was compared to that of existing variation reduction methods (peak correction and bin or LOESS GC correction) and trisomy prediction methods (standard Z-score, NCV and MAD-based Z-score) (Fig. 1). We included all methods used, except peak correction and the MAD-based Z-score, in NIPTeR, an R package publicly available under the GNU GPL open source license on CRAN and at https://github.com/molgenis/NIPTeR.

We focused on whole genome sequencing analysis, in which the fraction of sequenced reads originating from the chromosome of interest in the sample is compared with that of a set of non-trisomy control samples. In all analyses, only data from autosomal chromosomes was used.

Each chromosome was partitioned into bins of 50,000 base pairs. This bin size is in line with previous methods^3, 5–7, 9^. In each bin, the number of reads aligned to the forward and reverse strands reads were counted. The bin counts were used as the basic components for all further processing.

### Chi-squared-based variation reduction

The novel χ^2^VR reduces the weight of the number of reads in bins that have a higher variation than expected by chance and thus reduces the impact of these bins on the chromosomal fractions. No prior knowledge on the origin of the variation is needed. The χ^2^VR performs a sum of squares calculation: per bin, the sum of the chi-squared value is calculated over all the selected control samples. For this calculation, the observed read counts *o* are first normalized by multiplying them with a normalization factor. This factor is the mean number of observed total read counts for all autosomal bins *i* of all control samples *j* divided by the mean number of observed total read counts for all autosomal bins of the sample *s*. In short, the observed normalized read count for a specific bin (*on*_*i*_) can be calculated as follows:

1 $$o{n}_{is}={o}_{is}\times \frac{({\sum }_{ij=1}^{n}{o}_{ij})/({n}_{i}\times {n}_{j})}{({\sum }_{i=1}^{n}{o}_{is})/{n}_{i}}$$

where n_i_ is the number of bins and n_j_ is the number of control samples. Then, the chi-squared value for each bin *i* is calculated for each control sample *j* by dividing the squared difference between the expected and observed normalized read count by the expected normalized read count for that bin, where the expected normalized read count is the average normalized read count for a specific bin in all control samples (µ_ij_). The sum chi-squared value is calculated by adding up the chi-squared values of all the control samples for the bin:

2 $$\sum _{j=1}^{n}{\chi }_{ij}^{2}=\frac{{({\mu }_{ij}-o{n}_{ij})}^{2}}{{\mu }_{ij}}$$

The sum chi-squared value for each bin is transformed to a standard normal distribution *N*(*0*, *1*) by subtracting the degrees of freedom *df* (number of control samples minus one) from the sum chi-squared value and dividing this by the square root of two times the degrees of freedom.

3 $$N(0,1)=\frac{({\sum }_{j=1}^{n}{\chi }_{ij}^{2})-df}{\sqrt{2df}}$$

This results in a Z-score, which shows the number of standard deviations (SD) an observation differs from the expectation. Reads in bins with a Z-score higher than 3.5 are divided by the sum chi-squared value divided by the degrees of freedom, thereby reducing the variability between the samples. Normalized read counts in bins with a Z-score lower than 3.5 are not corrected. The justification for this procedure is that probability plots show the expected chi-squared distribution up to a Z-value of about 3.5. Values above 3.5 are much more frequent than would be expected, so instead of ignoring those bins we chose to reduce the weights, assuming that there is still information present in the over-dispersed bin counts. An overview of the analysis steps and their effects is shown in Supplement 1.

### Regression-based Z-score

The RBZ combines linear regression with a Z-score calculation. In the RBZ calculation the fraction of the chromosome of interest is predicted using stepwise regression with forward selection, in short forward regression. The reads aligned to the forward and reverse strands are used as separate predictors, because several chromosomes show a small, but consistent, over- or underrepresentation of reads aligned to the forward or reverse strand (Supplement 2). However, all reads aligned to the chromosome of interest are taken together rather than separated, because the higher number of reads leads to a lower variability in the number of reads aligned to the chromosome of interest.

For each chromosome of interest, the four best predictor sets, which each consist of four predictors, are determined by forward regression, using the adjusted R squared of the model as a selection criterion. The predictors can have either a positive or a negative correlation with the chromosome of interest. Within each predictor set only one predictor can be selected from each chromosome, limiting the risk of introducing bias.

Using the models created for each control sample *s* the expected chromosomal fraction (*ef*) is calculated for the chromosome of interest. Subsequently, the observed chromosomal fraction of the total read count of the chromosome of interest (*of*) is divided by this expected fraction. In combination with the standard deviation of the prediction, a Z-score is calculated for each sample. Because the mean of the control group after regression is one, the coefficient of variation of the control group has the same value as the SD.

In short, the RBZ can be formulated as:

4 $$\frac{o{f}_{s}/e{f}_{s}-1}{\sqrt{{\sum }_{j=1}^{n}{(o{f}_{j}/e{f}_{j}-\overline{of/ef})}^{2}/n-1}}$$

where *s* is the sample of interest, *j* is an individual control sample and n is the total number of control samples.

The RBZ not only uses information from chromosomes having a positive correlation of read counts with the chromosome of interest, but also from chromosomes showing a negative correlation. An overview of an example RBZ calculation is shown in Supplement 3.

### Match QC score

For the sample of interest, the novel Match QC score algorithm calculates how well the overall pattern of chromosomal fractions matches the pattern of the control samples. If the pattern of the sample differs too much from that of the controls, the sample does not fit within the control group, making the control set non-representative for the sample. Cut-offs are control-group-specific and can be set using the Match QC scores of the individual control group samples. The Match QC score uses the data used for trisomy prediction as input. Variation reduction, e.g. GC-correction or χ^2^VR, is applied before calculating the Match QC score.

To obtain the Match QC score, first the chromosomal fractions (*of*) are calculated for the sample and all control samples. This is done by dividing the (weighted or corrected) total read count of each chromosome by the total read count of all autosomal chromosomes, excluding chromosomes 13, 18 and 21. Subsequently, for each control sample, the sum of squared differences of the chromosomal fractions between the sample and the control for all autosomal chromosomes, excluding chromosomes 13, 18 and 21, is calculated.

In short, the Match QC score between a sample of interest *s* and an individual control sample *j* can be formulated as:

5 $$\sum _{k=1}^{m}{(o{f}_{ks}-o{f}_{kj})}^{2}$$

where *k* is the chromosome and *m* is the total number of chromosomes, excluding chromosomes 13, 18 and 21.

Smaller differences indicate a better match. An overall Match QC score is calculated by taking the average of the results of all samples. The formula for the overall Match QC score is:

6 $$\frac{{\sum }_{j=1}^{n}{\sum }_{k=1}^{m}{(o{f}_{ks}-o{f}_{kj})}^{2}}{j}$$

where *n* is the number of control samples.

### Validation of algorithms

#### Samples

To assess the effects of different variation reduction and trisomy prediction algorithms, we sequenced 128 non-trisomy and 43 trisomy samples using the SOLiD Wildfire platform (Life Technologies, Carlsbad, CA, USA) and 142 non-trisomy and 7 trisomy samples using the HiSeq 2500 platform (Illumina, San Diego, CA, USA). A further 34 non-trisomy samples had an alternative plasma-isolation and were sequenced on a HiSeq. The trisomy status of all samples was determined using karyotyping or quantitative fluorescence PCR following amniocentesis or chorionic villi biopsy.

Samples were selected in accordance with and as part of the trial by Dutch laboratories for evaluation of non-invasive prenatal testing (TRIDENT) program, supported by the Dutch Ministry of Health, Welfare and Sport (11016-118701-PG). The program was also approved by the Ethics Committee of the University Medical Center Groningen. All participants signed an informed consent form.

#### Plasma isolation, sample preparation and sequencing

Plasma was obtained from two different sources. The first source was fresh EDTA blood, either processed within 3 hours of blood collection or within 24 hours if stabilizing reagent was present in the tubes (Streck Inc., Omaha, NE, USA). For samples sequenced using the Illumina platform, blood was first centrifuged at 1200 rcf for 10 minutes, without using brakes to stop the rotor. The plasma was then transferred to another tube and centrifuged at 2400 rcf for 20 minutes. The plasma was transferred to a third tube and stored at −80 °C. For samples sequenced on the SOLiD platform, the centrifugal forces used were 1600 rcf and 16000 rcf, respectively. The second source of plasma was obtained using an alternative isolation method using only the first centrifugation step at 1200 rcf, after which the blood plasma was stored at −20 °C.

For samples sequenced on the HiSeq, we isolated cell-free DNA (cfDNA) from 1.5 ml plasma with the QIAamp MinElute Virus Spin kit (Qiagen, Valencia, CA, USA) (90 non-trisomy and 6 trisomic samples), the Qiagen circulating nucleic acid kit (Qiagen) (21 non-trisomy samples) and the Akonni TruTip kit (Akonni Biosystems, Frederick, MD, USA) (31 non-trisomy samples and 1 trisomic sample). After DNA isolation, sample preparation was performed with NEBNext Multiplex Oligos for Illumina (New England Biolabs Inc., Ipswich, MA, USA). Before the amplification step, we performed a two-step size selection using Agencourt AMPure xp beads (Beckman Coulter, Inc., Brea, CA, USA), using a beads/sample ratio of 0.6:1 in the first step and a ratio of 1.2:1 in the second step. Samples were sequenced with a 50 bp read length on a HiSeq 2500 sequencing platform (Illumina).

For samples sequenced on the SOLiD, cfDNA was extracted from 1 ml plasma using the QIAamplDSP DNA blood mini kit (Qiagen). Libraries were prepared according to factory protocol and sequenced with a 35 bp read length on the SOLiD 5500 Wildfire sequencing platform (Life Technologies).

#### Read alignment

For Illumina data, after an initial quality control of the fastq data using the program fastqc (v.0.7.0), the data were aligned to the human reference genome build b37 as released by the 1000 Genomes project^12^ using BWA aln samse (0.5.8_patched) with default settings^13^. After alignment a Sam output file^14^ was created for each sample. Using Picard tools 1.6.1, a set of tools designed by the Broad Institute (Cambridge, USA) (http://broadinstitute.github.io/picard/) for processing and analyzing next generation sequencing data, the Sam files were transformed into Bam files. These Bam files were sorted and Bam index files formed. The Bam index files link the reads to the genome position. Quality metrics files were then created and the duplicate reads in the Bam files marked.

For SOLiD data, raw reads were mapped against the human reference genome (GRCh37/ hg19) using BWA v0.5.9^13^. Options used for mapping were −c, −l 25, −k 2, and −n 10. The Bam files were filtered using Sambamba v0.4.5^15^ to retain non-duplicate reads, uniquely mapped reads (XT:A:R), reads with no mismatches to the reference genome (CM:i:0), and reads with no second best hits in the reference genome (X1:i:0).

After filtering and removal of duplicate reads, the total autosomal read count was on average 20.2 million (SD 5.6 million) for SOLiD data and 12.5 million (SD 2.2 million) for Illumina data.

#### Variation reduction

Aligned reads were divided into 50,000 bp bins and variation between samples was reduced using all possible combinations of zero or more variation reduction methods: peak correction, GC-correction and χ^2^VR. When more than one method was used, they were performed in the order described above (Fig. 1). A maximum of one GC-correction method was used. Since the LOESS GC-correction has been described more often^5–8^ than the weighted bin GC-correction^9^, we used LOESS GC-correction to evaluate the other variation reduction and prediction methods.

#### Peak correction

Peak correction was performed as described by Fan and Quake^9^. This method removes bins having a read count that significantly differs from the average using the information of all control samples. A bin was considered to deviate from normal if the total read count fell outside 1.96 SD compared with total read counts in the bins on the same chromosome for that sample. We interpreted bins to have a consistent pattern of region-specific variations if the variation deviated from normal in 95% or more of the control samples.

#### GC-correction

An important factor explaining the systematic uncontrolled variation between chromosomes is the guanine and cytosine (GC) content of the DNA fragments analyzed. When this GC-bias is corrected during preprocessing of the data, it results in a significantly lower variability^8^. GC-correction was performed based on total read counts using two different methods. The first GC-correction method is based on a LOESS curve fitted to the reads counts in bins sorted on GC content^5–8^ and based on R v3.0.2 default settings (span 0.75; degree = 2). The second GC-correction method is based on the average coverage of bins having a similar GC-content^9^. The GC% of each bin is determined for both methods. Bins not containing any reads and bins with an unknown base composition are ignored. The weights of the correction factors were based on GC-content intervals of 0.1% and consisted of the average coverage of the bins within the GC-interval divided by the average coverage of all bins.

#### Trisomy prediction

We predicted trisomies using four different prediction methods: standard Z-score prediction^5^, NCV, using only the most informative chromosomes^10^, MAD-based Z-score^11^ and RBZ. Depending on the variation reduction methods employed, we used corrected or uncorrected read counts for prediction. For all analyses chromosomes 13, 18 and 21 were not used as predictor chromosomes, since the prediction would be affected if a trisomy was present in one of the chromosomes used for prediction.

In short, the standard Z-score calculates the fraction of reads originating from the chromosome of interest compared with all reads originating from autosomal chromosomes, and then subtracts the mean fraction – which is the expected fraction – of the chromosome of interest in a set of control samples. The result is then divided by the SD of the fraction in the control set.

The NCV does not use all the autosomal chromosomes to calculate the fraction of the chromosome of interest, instead using the most informative chromosomes, which were selected using a training set^10^. All combinations of denominator chromosomes were tested for both the Illumina and SOLiD datasets, and the combinations yielding the lowest CVs were selected. The NCV is sometimes compared to using an internal reference^6^ because, during analysis, the selected reference chromosomes behave similarly to the chromosome of interest. This positive correlation results in less sample to sample variation, reduces the need for GC correction, and increases prediction precision.

The MAD-based Z-score replaces the SD by 1.4826 * MAD, making the calculation more tolerant of outliers in the control set^11^. The MAD was calculated in three steps. First, the median of the fractions of the chromosome of interest in the control set was calculated. Second, the absolute difference of the chromosomal fraction to the median was calculated for each control sample. Finally, the MAD was calculated by taking the median of these absolute differences.

#### Comparison of the algorithms

In comparing the algorithms we used the CV as a benchmark for performance. The CV is a standardized measure of dispersion of a probability distribution and is defined as the ratio of the SD to the mean. In this manner it enables comparison between normal distributions with a different mean. The height of the CV of the control group, together with the percentage cffDNA, determines the discriminative power between normal and trisomic samples. When the CV decreases, the sensitivity increases (Supplement 4). We determined the added value of each variation reduction or prediction algorithm to lowering the CV to determine the best combination of algorithms.

For our analysis, we used all the non-trisomy samples sequenced with the same platform that underwent the same plasma isolation procedure as control samples. This resulted in control group sizes of 142 for the Illumina and 128 for the SOLiD sequencer. For all algorithms, the control group is only used when it is normally distributed as determined using the Shapiro Wilk statistical test (p > 0.05).

#### Algorithm combinations tested

We evaluated the effects of both peak correction and χ^2^VR on the CV of the control samples, the effect of the two different GC correction methods in combination with all prediction methods on the CV, and the effect of the different prediction methods on CV and Z-scores in combination with all possible variation reduction methods, except peak correction and the bin GC correction. The consistency of the RBZ trisomy prediction was determined by estimating three additional trisomy prediction models for each analysis.

### Match QC score

To provide a proof of principle for the Match QC score performance, we divided the Illumina control group into a training set of 85 and a test set of 57 samples. The 34 Illumina samples that underwent a different plasma isolation protocol were used as an example of samples having undergone an alternative procedure.

We then calculated the Match QC score for all samples, using uncorrected, χ^2^VR, LOESS GC, and combined LOESS GC and χ^2^VR-corrected data. Cut-offs for the Match QC score were set on the average Match QC of the training set plus three SD. For all samples Z-scores were calculated for chromosomes 13, 18 and 21 to determine whether the scores fall within three SD of the average of the control set.

## Results

For both the SOLiD and Illumina control groups, the CV of chromosomes 13, 18 and 21 was determined for all combinations of variation reduction and trisomy prediction methods and their theoretical effect on sensitivity and specificity was calculated (Supplement 5). The estimated percentages of cffDNA in the tested trisomy samples are shown in Supplement 6.

### Effect of peak correction

**Figure 2:**
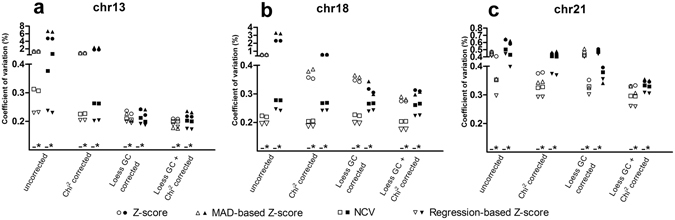
Effect of peak correction on the CV of control samples. The effect is shown for SOLiD (white) and Illumina data (black) with no other correction, for data that also had a chi-squared correction, or LOESS GC correction, or both LOESS GC and chi-squared correction. For each type of correction the CV of four prediction algorithms (standard Z-score, MAD-based Z-score, Normalized Chromosome Value and regression-based Z-score) are shown for (**a**) chromosome 13, (**b**) chromosome 18 and (**c**) chromosome 21. ^–^not peak corrected; *peak corrected.

To examine the effect of correcting bins with a coverage that deviates significantly from the average, we compared the CV of the peak-corrected data with that on which no peak correction was performed. Peak correction reduced the CV in most analysis strategies (Fig. 2). The largest relative effect for all chromosomes was observed when a GC-correction was also performed. The effect was largest in chromosome 21, which was the chromosome showing the lowest GC-bias when no correction was applied, suggesting that the influence of coverage peaks on variability only comes to light when GC-bias is limited. In data that was also χ^2^VR corrected, the variation did not further decrease but it did sometimes increase after use of a peak correction. This suggests that the peak correction and the χ^2^VR are partly correcting the same sources of bias.

### Effects of the two GC correction methods

**Figure 3:**
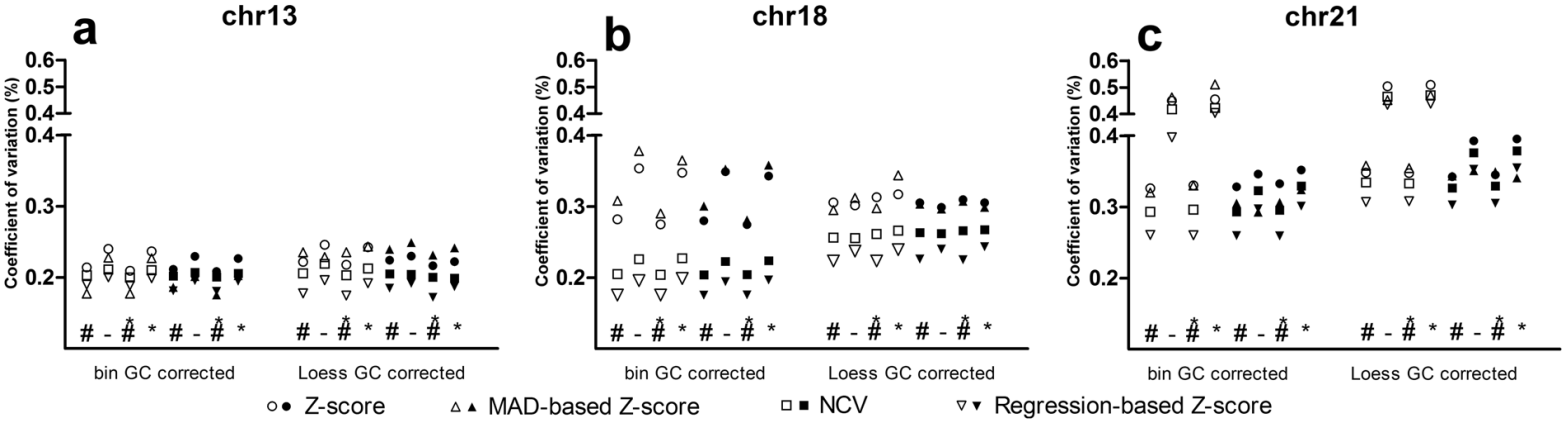
Comparison of the effect of two GC correction methods (bin GC correction and LOESS GC correction) on the CV of the control samples. SOLiD data (white) and Illumina data (black). For each type of correction the CVs of four prediction algorithms (standard Z-score, MAD-based Z-score, Normalized Chromosome Value and regression-based Z-score) are shown for (**a**) chromosome 13, (**b**) chromosome 18 and (**c**) chromosome 21. ^#^Chi-squared corrected; ^–^not corrected; *peak corrected.

To examine the performance of the weighted bin GC correction and the LOESS GC-correction, we compared the performance of both methods in combination with all other variation reduction and prediction methods for chromosomes 13, 18 and 21 (Fig. 3). For chromosome 13, both GC correction methods performed equally well regardless of the other variation reduction and prediction methods used. For chromosome 18, the weighted bin GC correction had a better performance for the NCV and RBZ compared to LOESS GC correction. However, the Z-score and MAD-based Z-score predictions performed better using the LOESS GC-correction. For chromosome 21, the weighted bin GC correction performed best, regardless of the other methods used. The data sets used made no difference to the performance of either GC-correction method.

### Effect of chi-squared-based variation reduction

**Figure 4:**
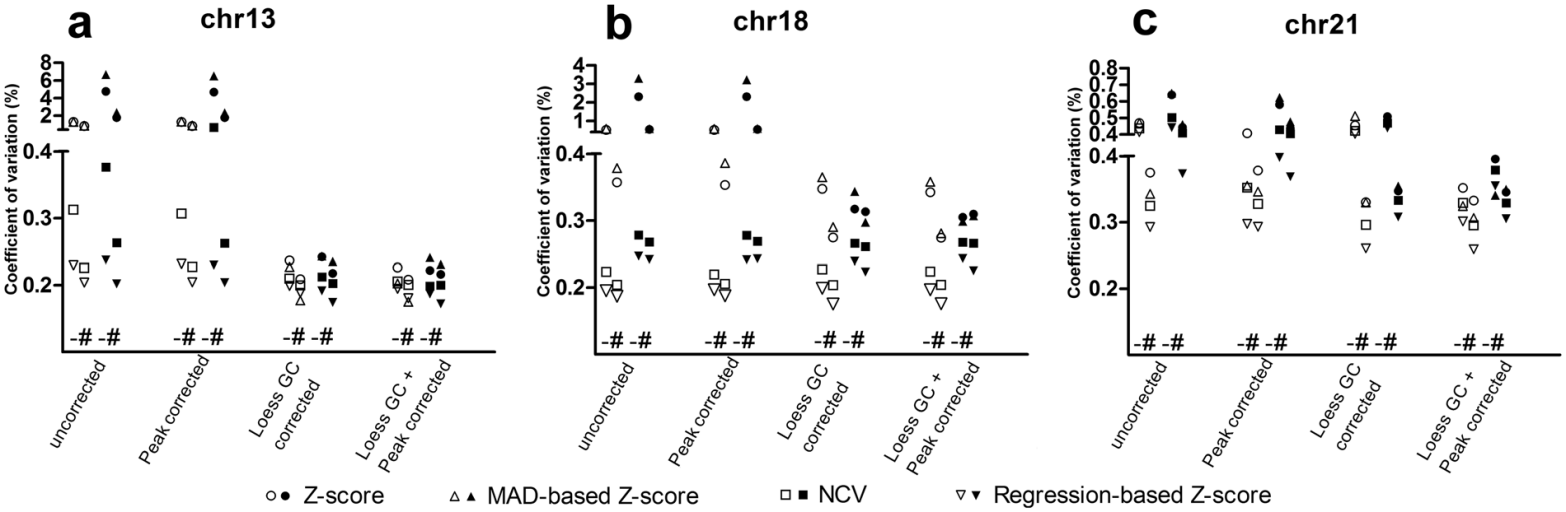
Effect of chi-squared-based variation reduction on the CV of control samples. SOLiD (white) and Illumina data (black) with no other correction, or with a peak correction, or LOESS GC correction or both LOESS GC and peak correction. For each type of correction the CVs of four prediction algorithms (standard Z-score, MAD-based Z-score, Normalized Chromosome Value and regression-based Z-score) are shown for (**a**) chromosome 13, (**b**) chromosome 18 and (**c**) chromosome 21. ^–^not chi-squared corrected; ^#^chi-squared corrected.

To examine the performance of the χ^2^VR, we compared the control group CV using all other variation and prediction methods, with and without the χ^2^VR (Fig. 4). The χ^2^VR resulted in a lower CV in most analysis strategies for all chromosomes. The effect was most striking in chromosome 21, regardless of the other methods used.

### Effect of trisomy prediction algorithms

**Figure 5:**
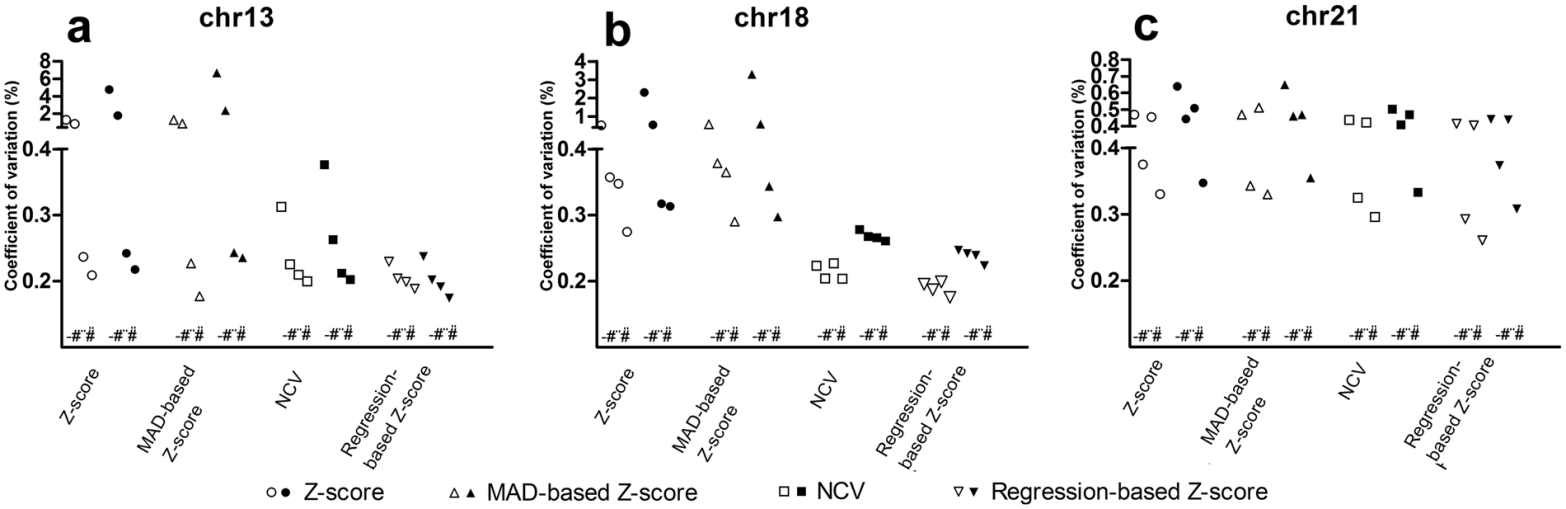
Effect of the different prediction algorithms on the CV of control samples. SOLiD data (white) and Illumina data (black). Results from the four different prediction algorithms (standard Z-score, MAD-based Z-score, Normalized Chromosome Value and regression-based Z-score) are shown for (**a**) chromosome 13, (**b**) chromosome 18 and (**c**) chromosome 21. ^–^Variation was not reduced, ^#^chi-squared corrected, “ LOESS GC corrected, #” both LOESS GC and chi-squared corrected before prediction.

To examine the effect of the prediction algorithms (standard Z-score, MAD-based Z-score, NCV and RBZ), we compared the CV using uncorrected, χ^2^VR, LOESS GC, and combined χ^2^VR and LOESS GC corrected data. Since the peak correction provides no added value to the χ^2^VR, it was not used for comparison. The RBZ produced the lowest CV for all variation reduction methods except the SOLiD combined LOESS GC and χ^2^VR corrected data, in which the MAD-based Z-score for chromosome 13 produced an even lower CV (Fig. 5). The variation using the NCV is higher than that using the RBZ, but the CV is still much lower than the CVs of the methods that used all autosomal chromosomes. The standard Z-score had the highest coefficient of variation in all models.

A lower CV yields a more extreme Z-score, which means that in the case of a trisomy, the Z-score is more likely to be higher than the threshold, resulting in a higher sensitivity. The Z-scores of the trisomy samples of the four prediction algorithms for the uncorrected, χ^2^VR, LOESS GC, and combined χ^2^VR and LOESS GC corrected data are listed in Supplement 7. False-negative and false-positive results were determined for all the above combinations of variation reduction algorithms and prediction algorithms, based on a 99.7% confidence interval (Z-score threshold of three) (Supplement 8).

Of the 50 trisomic samples, a false-negative result was found in two trisomy 13 and three trisomy 18 samples for the Z-score or the MAD-based Z-score when no variation reduction was done. One confirmed trisomy 18 sample did not give a positive result with any combination of algorithms, possibly due to a low fetal percentage. No false-negatives were found for chromosome 21. For all true-positive results, all four RBZ models showed a Z-score higher than three.

**Figure 6:**
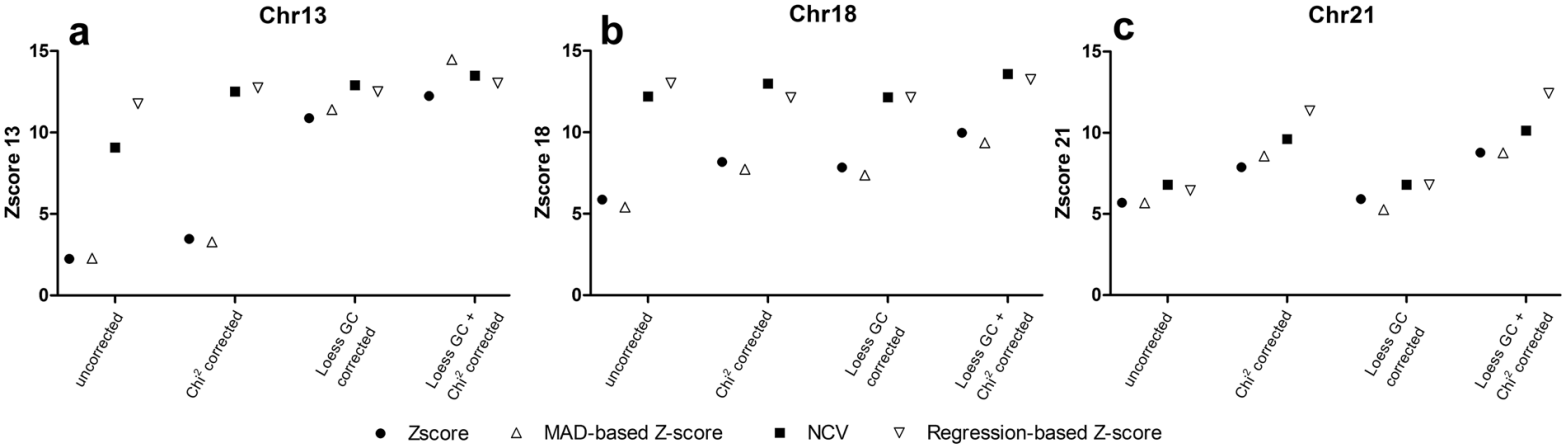
Z-scores for three trisomies using different combinations of variation reduction and prediction algorithms. All three examples are based on SOLiD data. Results from the four different prediction algorithms (standard Z-score, MAD-based Z-score, Normalized Chromosome Value, and regression-based Z-score), in combination with uncorrected, chi-squared corrected, LOESS GC corrected, and both LOESS GC and chi-squared corrected are shown for (**a**) chromosome 13, (**b**) chromosome 18 and **(c)** chromosome 21.

To better show the effect of the different variation reduction and prediction algorithms on the Z-score, we selected three samples, sequenced on the SOLiD platform, each having a trisomy 13, 18 or 21 (Fig. 6). Based on the Z-scores and CVs, each sample had an estimated fetal percentage of 5–6%. The NCV and RBZ consistently yielded higher Z-scores than the standard Z-score and the MAD-based Z-score. The effect of the GC-correction is reflected in the results of the standard Z-score and the MAD-based Z-score for chromosome 13 and the effect of the χ^2^VR shows in the chromosome 21 results.

Of the 270 non-trisomy samples, four samples showed a false-positive result for more than one prediction algorithm. For one sample, all four prediction methods showed a result higher than three. The more sensitive NCV and RBZ prediction methods resulted in more false-positive results than the standard Z-score or MAD-based Z-score because more parameters are estimated, which leads to some overfitting and therefore underestimation of the prediction accuracy for new samples. This effect will be reduced when larger control groups are used. Three other false-positive results were only seen in one of the variation reduction methods, one for NCV and three for RBZ. In all these cases, Z-scores were just above three. In all cases adding or removing a variation reduction step, resulted in a negative call. For samples having a false-positive RBZ result, at least one of the additional RBZ predictions resulted in a negative prediction, except for the sample having a Z-score higher than three in all prediction methods.

### Match QC score

**Figure 7:**
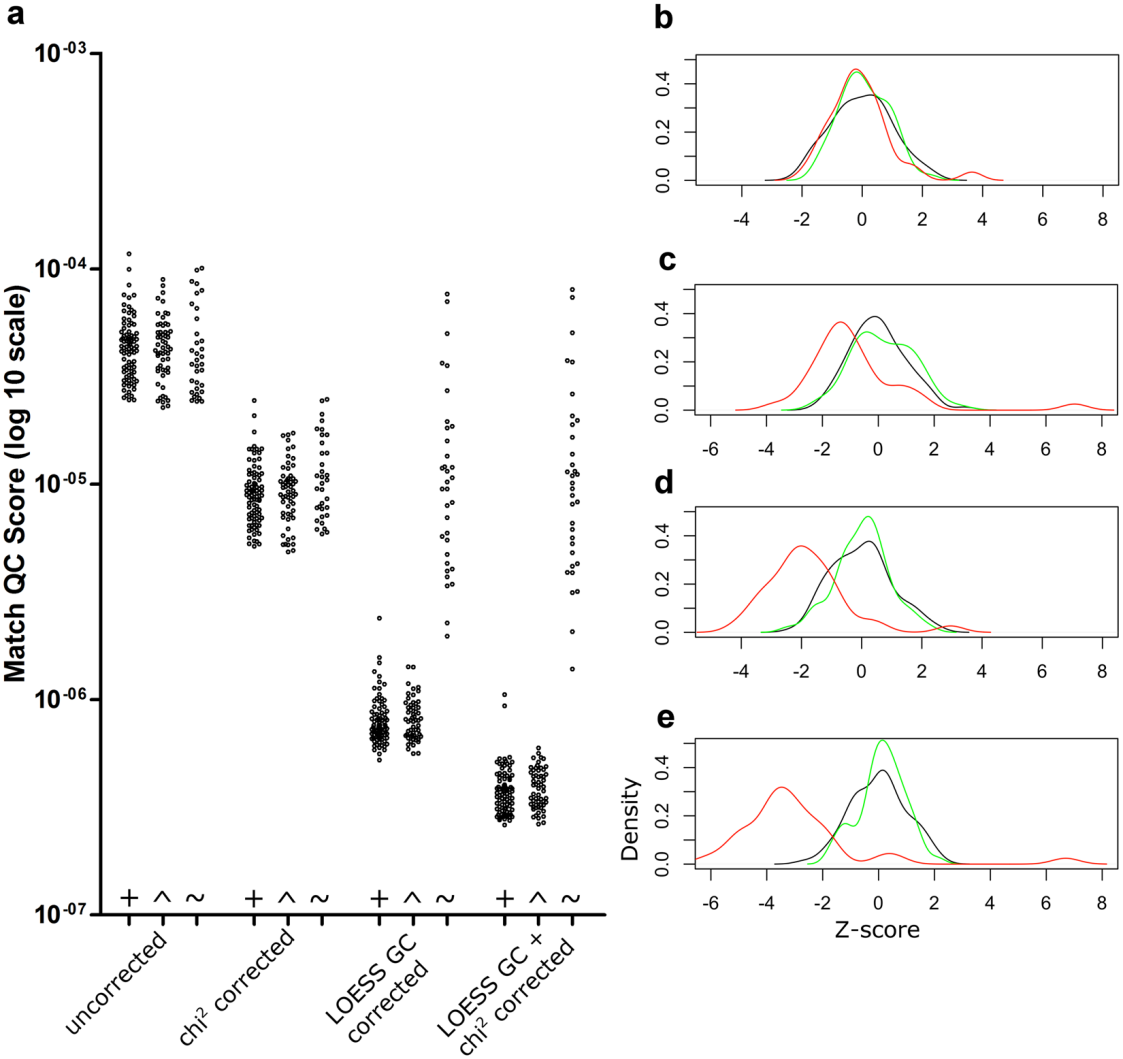
Match QC scores and Z-scores for matching and non-matching samples. (**a**) Match QC scores per sample for uncorrected, chi-squared corrected, LOESS GC corrected and both LOESS GC and chi-squared corrected data for the control group, matching samples, and non-matching samples. Chromosome 21 Z-scores for (**b**) uncorrected data, (**c**) chi-squared corrected data, (**d**) LOESS GC corrected data and (**e**) both LOESS GC and chi-squared corrected data. + and black line, control group samples; ^ and green line, samples that underwent the same sample preparation procedure; ~ and red line, single centrifugation plasma samples.

To examine whether the Match QC score could accurately predict whether a sample fits within a control group, we calculated the Match QC scores and all the Z-scores for a training set, a test set of samples that had been prepared in the same manner as the training set, and a third set of samples originating from single centrifuged plasma. For all three sets, we used uncorrected, χ^2^VR, LOESS GC and combined χ^2^VR- and LOESS GC-corrected data (Fig. 7). Test set samples had Match QC scores in the same range as the training set samples and Z-scores that fell within three SD of the mean for all types of corrected data. Single centrifuged samples, however, showed Match QC scores in the same range as the control group samples for uncorrected and χ^2^VR corrected data, but above the three-SD threshold for LOESS GC corrected data and combined LOESS GC- and χ^2^VR-corrected data.

Z-score distributions for the training set samples and the test set samples were indistinguishable for all correction methods, but Z-scores based on uncorrected or χ^2^VR corrected data were not normally distributed for chromosomes 13 and 18. For the single centrifuged samples, Z-scores did not deviate from the normal distribution for the uncorrected data of chromosome 21. Match QC scores for all the samples analyzed, thresholds and Z-score distributions for chromosomes 13, 18 and 21 are shown in Supplement 9.

## Discussion

We show that both the χ^2^VR and the RBZ reduced the variability of the NIPT result and thus increased its sensitivity in both Illumina and SOLiD data. Furthermore, we show that a Match QC exceeding a three-SD threshold, determined using control samples, identified those samples for which the controls were not representative. Although the algorithms described in this study are designed to improve analysis of NIPT data, they may also be of use in similar types of analyses that need high sensitivity such as copy number variation detection in liquid biopsy data^16, 17^.

The lower variability between samples decreases the percentage of fetal DNA needed for NIPT. A low percentage of fetal DNA is an important contributor to false negative or inconclusive results^18^. Moreover, the average percentage of fetal DNA is lower in trisomy 13 and trisomy 18 pregnancies than in non-trisomy pregnancies^19, 20^. A low variability is therefore even more important for these pregnancies for the test to have a high sensitivity. Moreover, our novel algorithms produce a lower variability for a given number of reads, resulting in the need for fewer reads and lowering sequencing costs. Alternatively, only DNA-fragments originating from regions of interest could be selected^21–23^. However, such a selection requires additional amplification during sample preparation, which could also create additional variation due to increased over-dispersion^24, 25^. We therefore chose to reduce variation by correcting for bias in read counts before analysis, leading to a more comparable distribution of reads over the chromosomes between samples. Other studies have shown that variability can be introduced by bias present in the data, such as GC-bias^3, 5–9^, or peaks of extreme coverage, probably caused by repeats^9^. However, due to a higher number of available reads, better results were obtained using a non-repeat-masked reference genome^5, 7^. For this reason, we did not mask any regions based on mappability tracks or blacklisted regions in our comparison.

In our comparison the lowest CVs for chromosomes 13, 18 and 21 were produced using the combination of the weighted-bin-based GC-correction method and the χ^2^VR with the RBZ. However, each variation reduction algorithm we tested reduced the variability when used alone. The effect of the peak variation reduction was small when combined with the χ^2^VR. This shows that the χ^2^VR corrects bias caused by regions of extreme coverage. Moreover, since the χ^2^VR focuses on variation present in each specific bin, and not on chromosomal averages, it can correct for variation that is too subtle for peak correction. And since no assumptions are made about the origin of the bias, no prior knowledge is needed for correction. However, when using the χ^2^VR on the X-chromosome, variability should be determined using only data from pregnancies of a female fetus to prevent variability in the fetal percentage adding to the total variability on that chromosome. After application of GC-correction, χ^2^VR reduced variation even further, suggesting that χ^2^VR corrects for sources of bias other than that from GC. Since up to 50% of the human genome is repetitive^26^, we suggest that part of the extra corrected bias is due to repeat structures. It has also been suggested that biological factors play a role in bias in NIPT^27, 28^, so part of the corrected bias might have a biological origin.

Where peak correction and χ^2^VR only remove reads to reduce variation, GC-correction removes reads in bins having a GC-percentage containing more reads than average, but it adds virtual reads in bins with a GC-percentage containing fewer reads than average. Although, after GC correction, more reads seem to be present for several chromosomes, dispersion is still based on the original number of reads aligned to those chromosomes.

We demonstrated that the prediction method used can also reduce variability and increase sensitivity. The RBZ resulted in the lowest variability and decreased the need for GC-correction because this method takes this kind of systematic bias into account. However, there may be some pitfalls. Similar to the NCV, prediction is based on a limited number of predictor chromosomes. The effect of an aberration in one of the predictor chromosomes on the prediction is larger for the RBZ and NCV than for the standard Z-score, which uses all autosomes for prediction. To limit the effect of possible aberrations, we recommend comparing four independent predictor sets for the RBZ. Conflicting results of different models are a warning of possible false-positive results. In our data, all 49 trisomies detected were predicted independently by the four RBZ prediction sets. Only one false-positive call was made by all four sets. This call was also made by all the other prediction methods, suggesting that there may indeed be a higher fraction of reads of the called chromosome present in the data. Since the NCV can be based on only one denominator chromosome, we suggest multiple predictions using different denominators should also be used for NCV.

Our results show that a Match QC score below the three-SD threshold does not guarantee that the control group is representative for a sample, but a score exceeding the threshold does indicate that the analysis is not accurate. The main assumption in NIPT analysis is that the control set is representative of the sample analyzed. A non-representative control set leads to an inaccurate prediction and possibly to false-positive or false-negative results. It is therefore important that all samples undergo the same preparation, sequencing procedure and bioinformatics analysis. However, even when standard procedures are used, bias can vary between sequencing runs^29^. Prediction methods with a higher sensitivity are more vulnerable to the effects of unaccounted biological variation because deviations in the expected chromosomal fractions will more rapidly lead to false-positive results. Sample quality metrics are therefore essential for reliable analysis.

Our study shows that both the χ^2^VR and the RBZ increase the sensitivity of NIPT compared to previously published methods. Furthermore, we show that the Match QC score identifies samples for which the non-trisomy control set was not informative. Moreover, these algorithms may have a broader applicability than NIPT analysis, for instance in analysis of copy number variations in liquid biopsy data. We recommend our novel algorithms, as included in the NIPTeR package, as a useful addition to the NIPT analysis toolbox, resulting in a higher sensitivity, in theory making it possible to detect trisomies in blood with a fetal DNA amount as low as 2%.

## Electronic supplementary material

Supplementary material
