# Supplementary material for: Novel Algorithms for Improved Sensitivity in Non-Invasive Prenatal Testing
Source: Sci Rep. 2017 May 12;7:1838. doi: 10.1038/s41598-017-02031-5 (PMC5431782; doi:10.1038/s41598-017-02031-5)

Supplementary material

Novel Algorithms for Improved Sensitivity in Non-Invasive Prenatal Testing

L. F. Johansson[1,2†*], E. N. de Boer[1†*], H. A. de Weerd[1,2], F. van Dijk[1,2], M. G. Elferink[3], G.H. Schuring-Blom[3], R. F. Suijkerbuijk[1], R. J. Sinke[1], G. J. te Meerman[1], R. H. Sijmons[1], M. A. Swertz[1,2] and B. Sikkema-Raddatz[1]

[1] University of Groningen, University Medical Centre Groningen, Department of Genetics, Groningen, the Netherlands

[2] University of Groningen, University Medical Centre Groningen, Genomics Coordination Centre, Groningen, the Netherlands

[3] University Medical Centre Utrecht, Department of Genetics, Utrecht, the Netherlands

[†]Contributed equally

*Correspondence should be addressed to L.F.J. (l.johansson@umcg.nl) or E.N.d.B. (e.n.de.boer@umcg.nl)

Lennart Johansson & Eddy de Boer

Department of Genetics, University Medical Centre Groningen

Hanzeplein 1

9713 GZ Groningen

The Netherlands

Contents

Supplement 1: Example of chi-squared based variation reduction for chromosome 21 3

Supplement 2: Consistent over- or underrepresentation of forward or reverse reads for specific chromosomes 7

Supplement 3: Example of regression model for chromosome 13 24

Supplement 4: Relation between coefficient of variation, percentage cell free fetal DNA and sensitivity 28

Supplement 5: Coefficients of variation for all combinations of variation reduction and trisomy prediction methods and their effect on sensitivity and specificity.. 30

Supplement 6: Percentage cell-free fetal DNA trisomy samples... 53

Supplement 7: Z-scores trisomy samples 54

Supplement 8: False positive and false negative results 56

Supplement 9: Match QC values and statistics... 60

Supplement 1: Example of chi-squared based variation reduction for chromosome 21

This supplement contains a series of graphs to visualize the effect of the chi-squared based variation reduction (χ^2VR). The input of the χ^2VR are sample and control group, bin-counts of uncorrected data or data corrected using different variation reduction methods, such as GC correction **(Figure S1.1)**. The examples are based upon the 142 Illumina control samples. In some images read counts of a single sample are shown. For these images a random sample was selected from the control group.

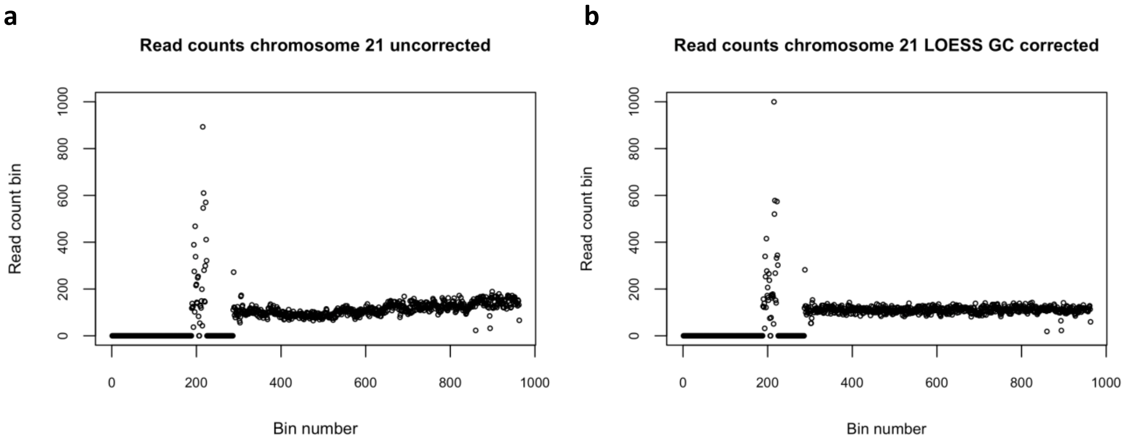

Figure S1.1: Read counts bins chromosome 21 without χ^2VR of one of the Illumina control group samples (a) uncorrected data. **(b)** LOESS GC corrected data.

First the data is normalized by dividing the mean read count of the bin by the mean read count of all autosomal bins. After this normalization sample read counts can be compared. In some of the bins the normalized read count is consistent between samples, resulting in a low coefficient of variation (CV). Other bins have a higher variability between samples, resulting in a higher CV **(Figure S1.2)**. A GC correction can correct part of the variation. However, even after GC correction some bins still show a high variation.

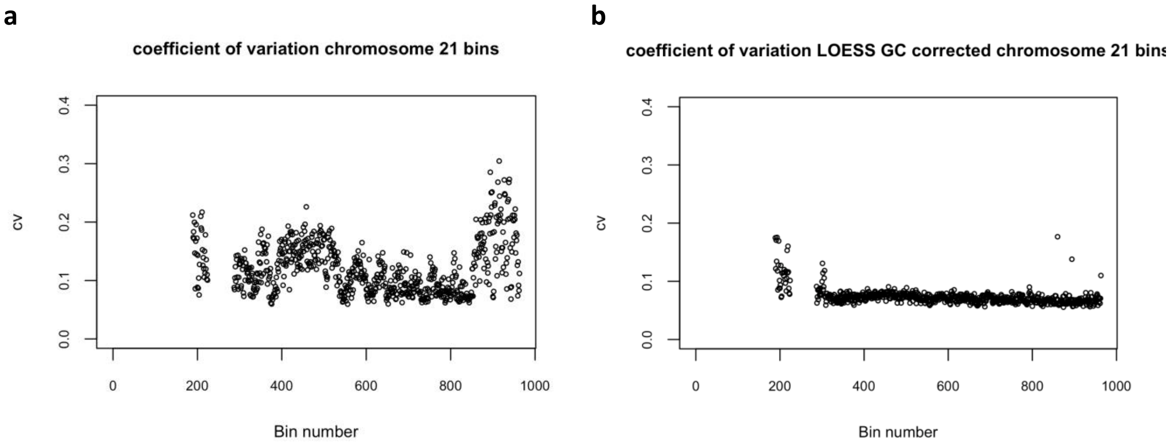

Figure S1.2: Coefficient of variation bins chromosome 21 without χ^2VR of the Illumina control group samples (a) uncorrected data. **(b)** LOESS GC corrected data.

After normalization for each bin the sum chi-squared value is calculated, using the control samples, and transformed to a standard normal distribution, resulting in a Z-score for each bin (**Figure S1.3)**.

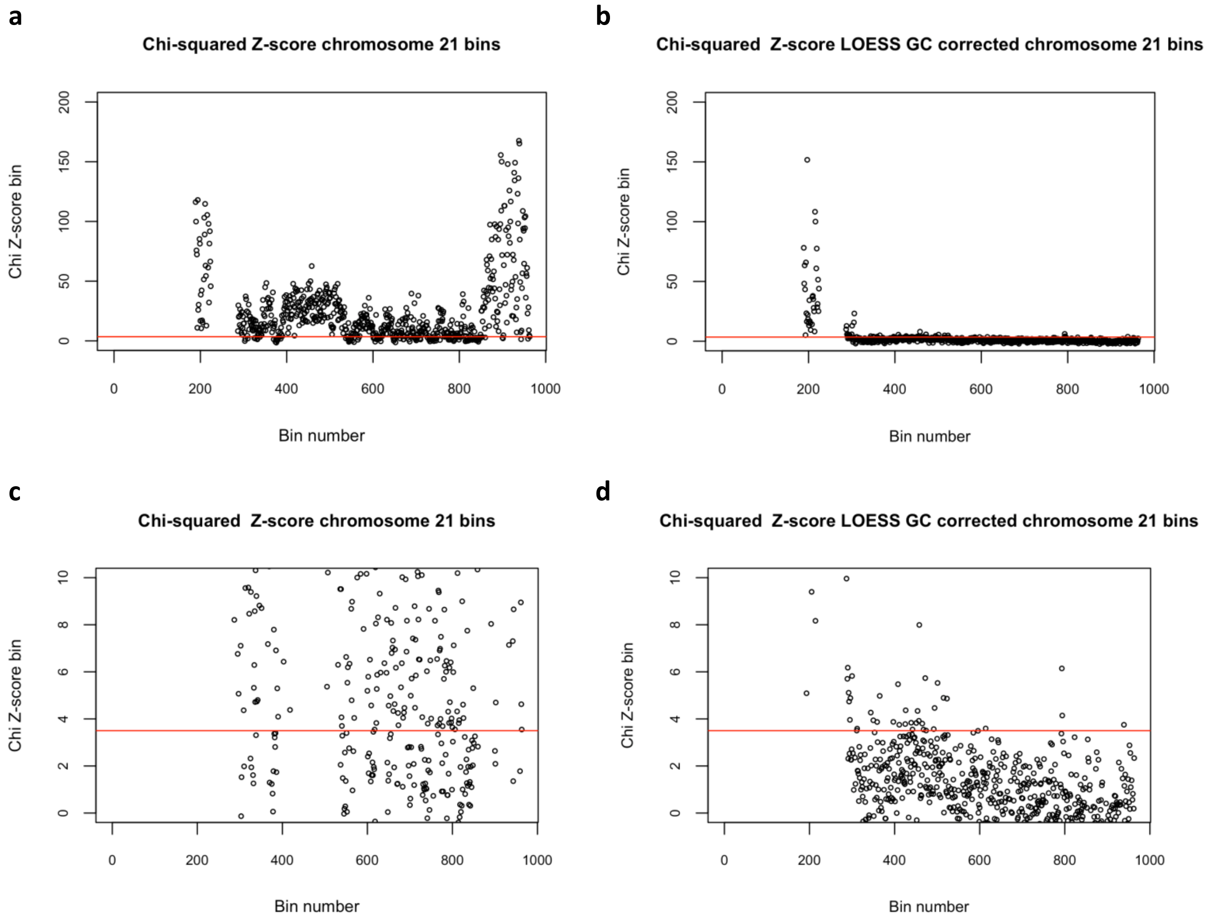

Figure S1.3: Z-score sum chi-squared value after transformation to normal distribution for all bins chromosome 21 based upon the Illumina control group samples (a) uncorrected data, total range. **(b)** LOESS GC corrected data, total range. **(c)** uncorrected data, plotted until a maximum Z-score of 10. **(d)** LOESS GC corrected data, plotted until a maximum Z-score of 10.

A threshold was set at a Z-score of 3.5. In the case all the variation was introduced by chance 99.9998% of the bins show a Z-score below 3.5. The variation in bins having a Z-score greater than 3.5 (overdispersed bins) is thus very unlikely to result from random variation and these bins have a higher variability than expected. The χ^2VR is based upon the assumption that there is still information present in the overdispersed bins. Instead of ignoring those bins, those exceeding the threshold will be weighted by dividing them by a correction factor **(Figure S1.4, Figure S1.5)**. The correction factor consists of the sum chi-squared value divided by the degrees of freedom.

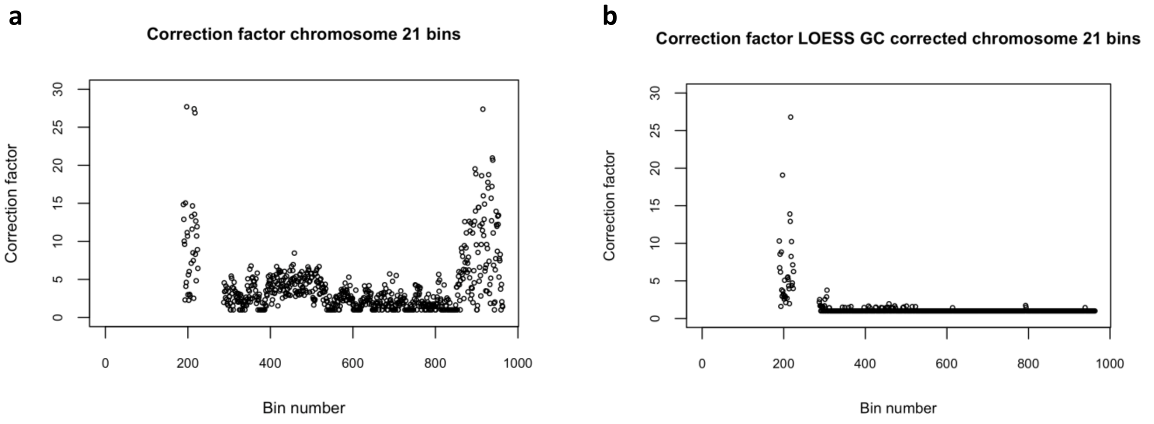

Figure S1.4: χ^2VR correction factor bins chromosome 21 based upon Illumina control group (a) uncorrected data. **(b)** LOESS GC corrected data.

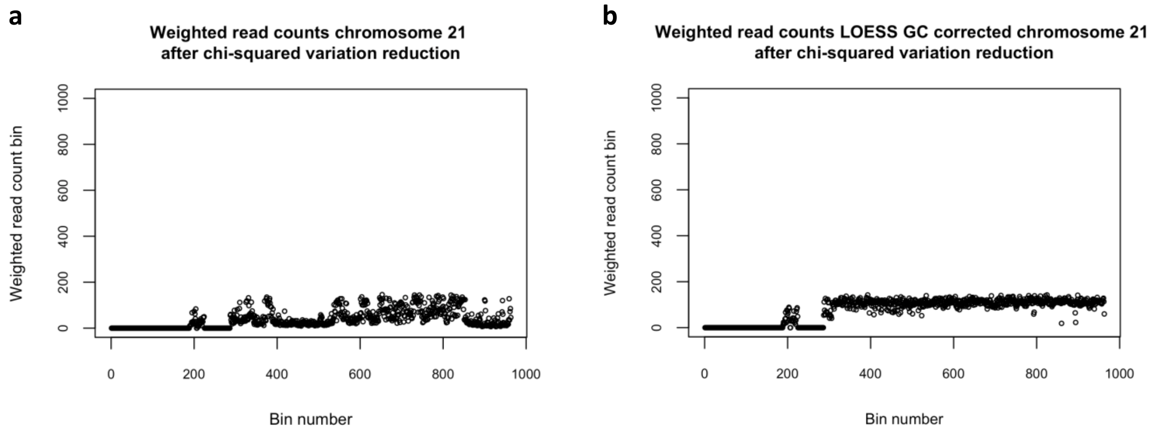

Figure S1.5: Weighted read counts bins chromosome 21 for one of the Illumina control group samples (a) uncorrected data. **(b)** LOESS GC corrected data.

Note that weighting read counts of overdispersed bins does not change the CV of those bins. Variability between samples is not affected at bin level. However, variability between chromosomal fractions is decreased after χ^2VR (**Figure S1.6)**. The chromosomal fractions are defined as the number of (weighted) read counts on chromosome 21 divided by the (weighted) read count of all autosomes. In figure S1.6 the fractions of chromosome 21 are normalized by dividing the fraction of each sample by the mean fraction of its control group.

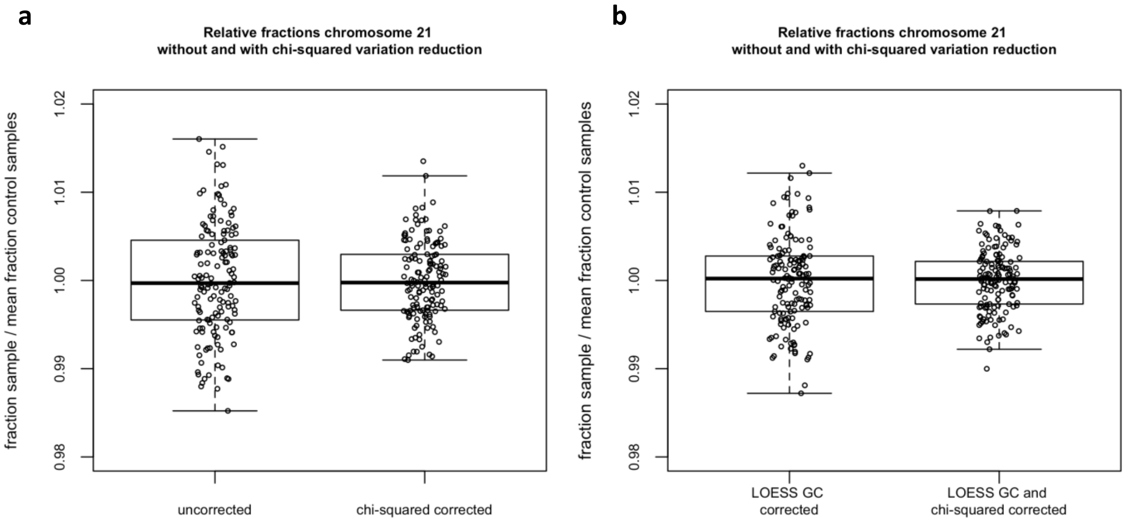

Figure S1.6: Relative fractions chromosome 21 before and after χ^2VR of Illumina control group samples (a) uncorrected data. **(b)** LOESS GC corrected data.

Supplement 2: Consistent over- or underrepresentation of forward or reverse reads for specific chromosomes

This supplement contains statistics and graphs that show that the fraction of reads mapping to the forward strand and the fraction of reads mapping to the reverse strand are not equal for all chromosomes. If the fraction of reads mapping to the forward strand was equal to that mapping to the reverse strand, the expected intercept of a regression model of the fraction of reads aligned to the reverse strand on the fraction of reads aligned to the forward strand of a specific chromosome would be expected to be equal to zero and the slope equal to one. This is indeed the case for some of the chromosomes **(Table S2.1)**, such as chromosome 19.

	Illumina		SOLiD	
	Intercept	Slope	Intercept	Slope
chr1	0.0011	0.9747	0.0011	0.9761
chr2	0.0016	0.9628	0.0125	0.7278
chr3	0.0006	0.9822	0.0021	0.9477
chr4	-0.0002	1.0059	0.0002	0.9979
chr5	0.0001	0.9971	0.0010	0.9707
chr6	0.0000	1.0011	0.0030	0.9092
chr7	0.0008	0.9717	0.0015	0.9465
chr8	0.0009	0.9655	0.0035	0.8747
chr9	0.0015	0.9320	0.0074	0.6417
chr10	0.0007	0.9728	0.0023	0.9081
chr11	0.0010	0.9607	-0.0001	1.0051
chr12	0.0033	0.8605	0.0089	0.6409
chr13	-0.0001	1.0044	0.0007	0.9630
chr14	0.0035	0.7854	0.0144	0.1365
chr15	0.0000	0.9986	0.0004	0.9748
chr16	0.0002	0.9889	-0.0001	1.0065
chr17	0.0000	0.9976	0.0001	0.9921
chr18	0.0002	0.9842	0.0014	0.9099
chr19	0.0000	0.9996	0.0000	1.0059
chr20	-0.0001	1.0049	0.0003	0.9737
chr21	0.0011	0.8453	0.0045	0.2991
chr22	0.0000	0.9966	0.0001	0.9804

Table S2.1 Intercept and slope of regression models of the fraction of reads aligned to the reverse strand on the fraction of reads aligned to the forward strand for all chromosomes of both the Illumina and the SOLiD control group.

However, not all chromosomes have equal numbers of reads aligned to the forward and the reverse strand. In some cases, especially in the SOLiD data, there is a consistent overrepresentation of reads aligned to the reverse strand (for instance in chromosome 4). Other chromosomes, for instance chromosome 14, show a low correlation between forward and reverse strand read counts. Regression plots are shown in **Figure S2.1**, in which the black line represents the regression line and the green line represents equal fractions of reads aligned to the forward and the reverse strand.

a1

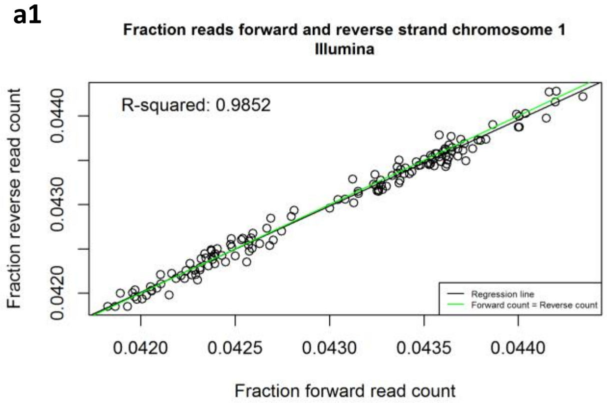

a2

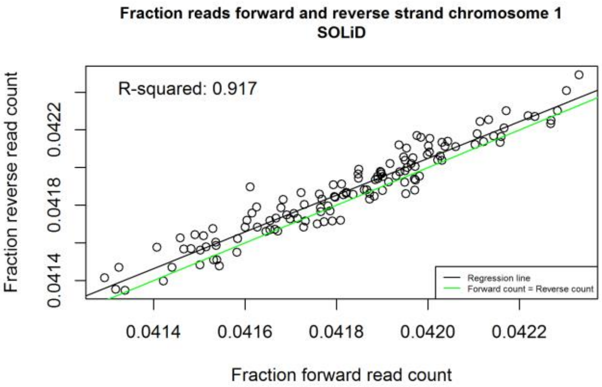

b1

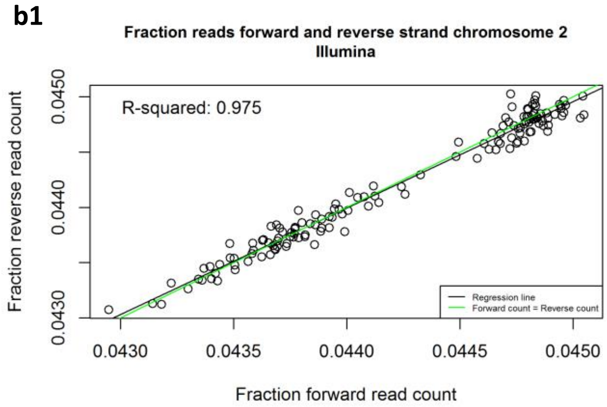

b2

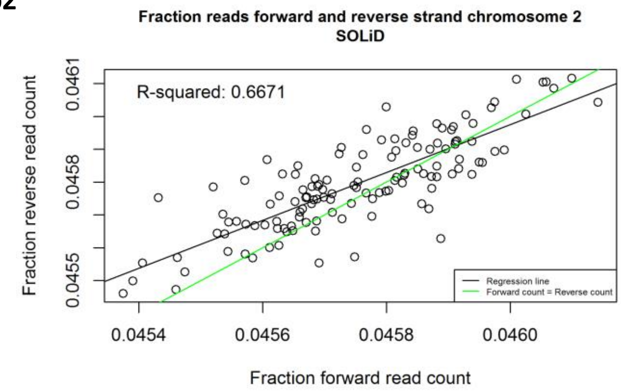

c1

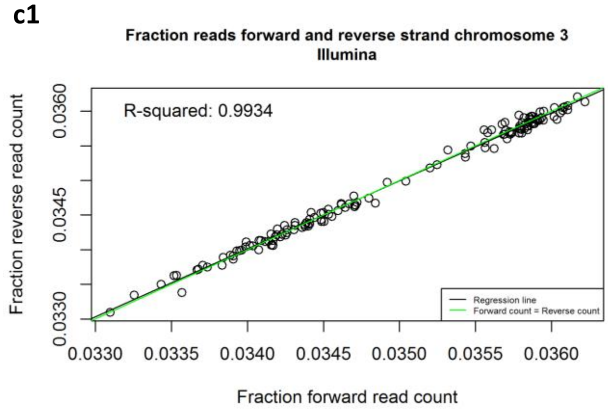

c2

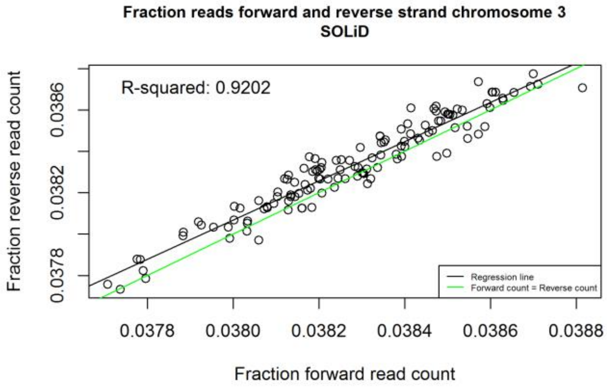

d1

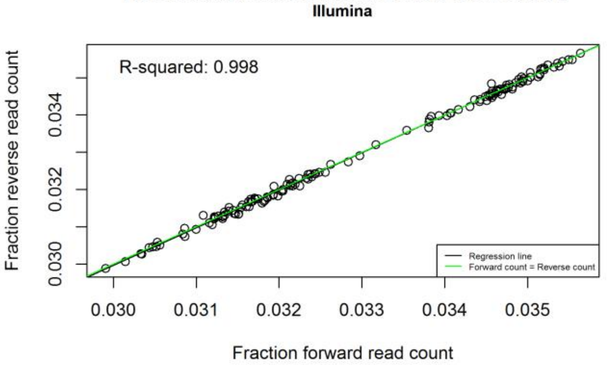

d2

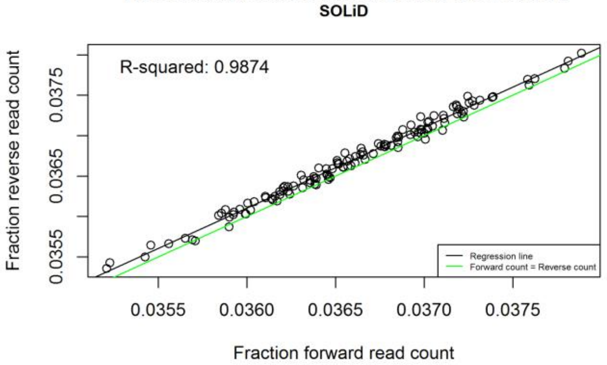

e1

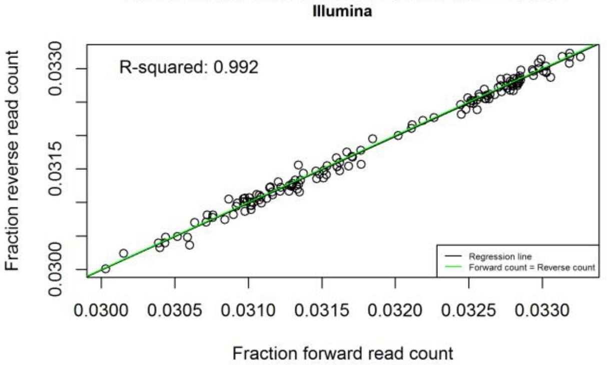

e2

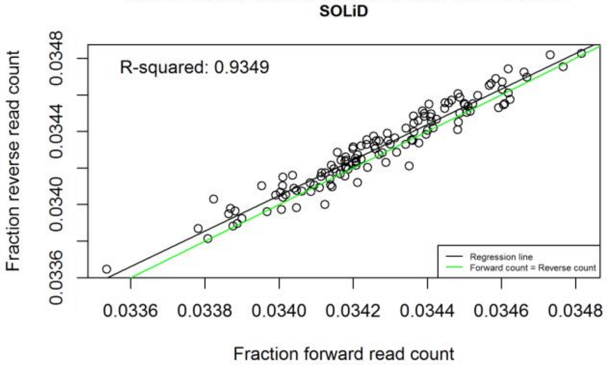

f1

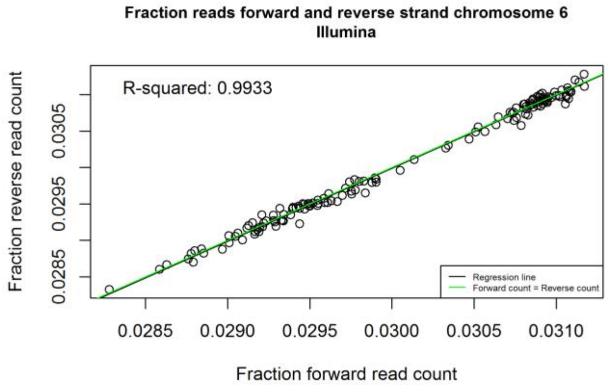

f2

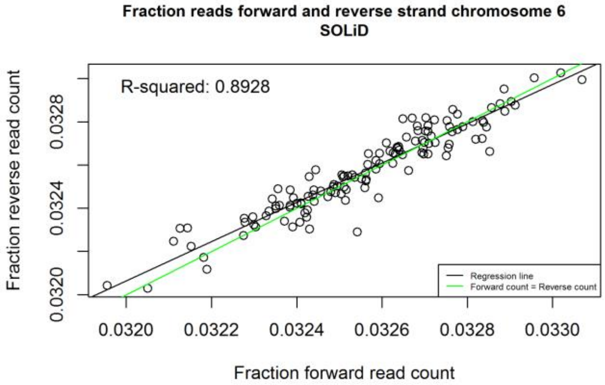

g1

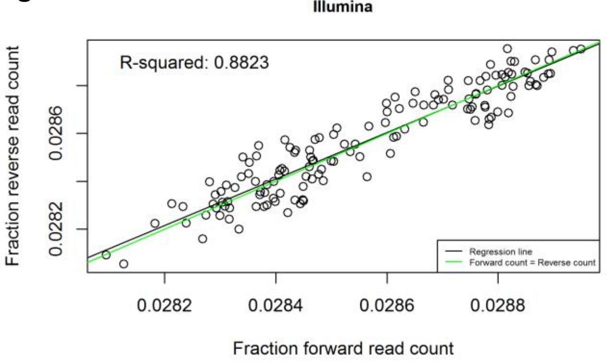

g2

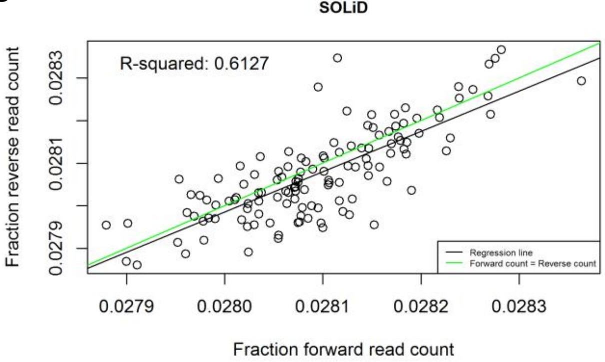

h1

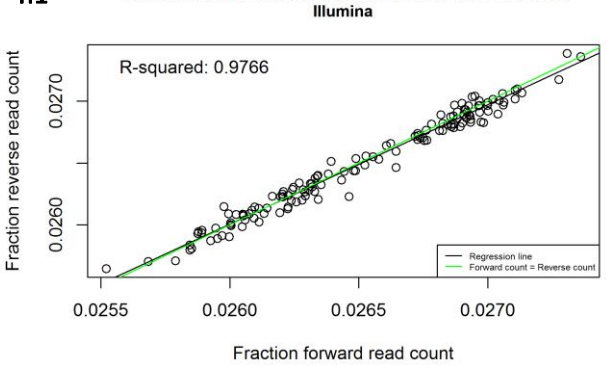

h2

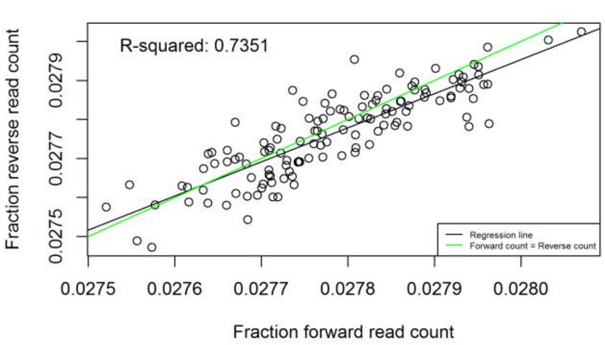

i1

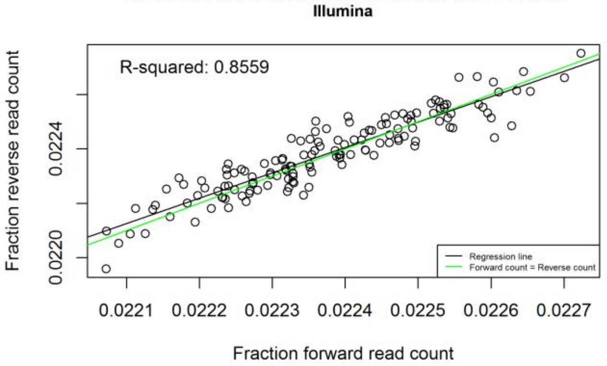

i2

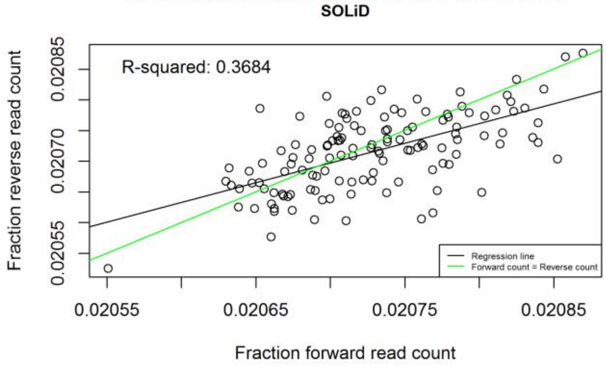

j1

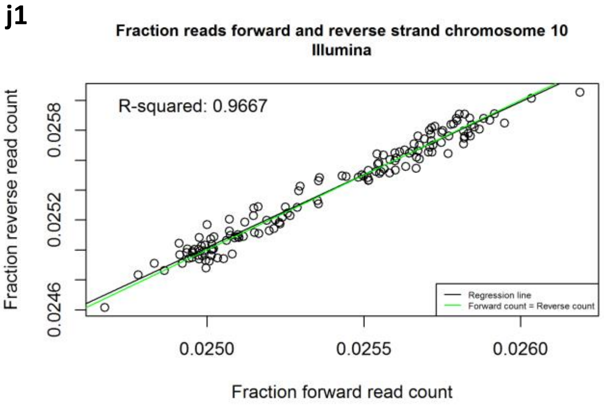

j2

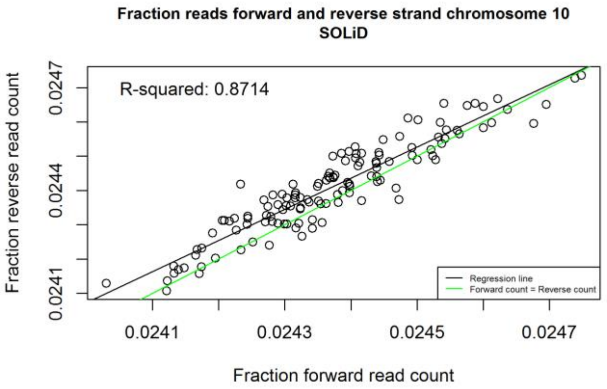

k1

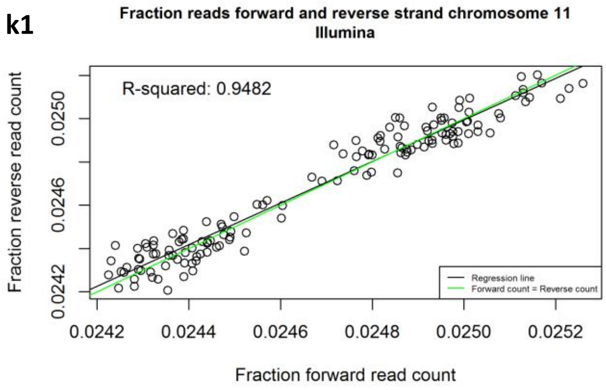

k2

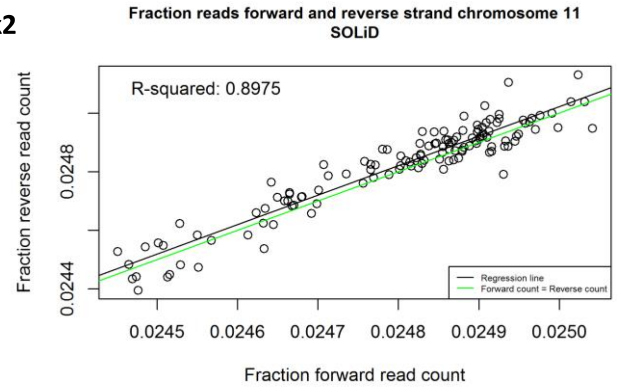

l1

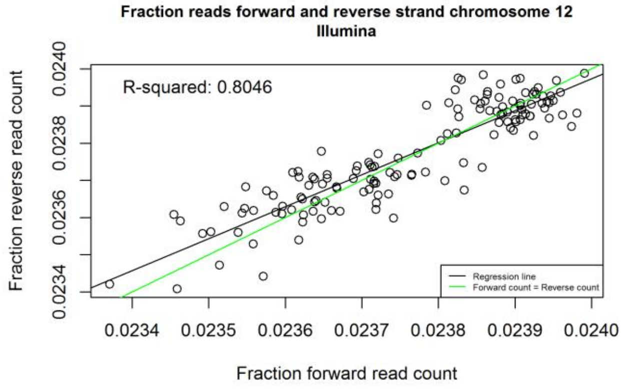

l2

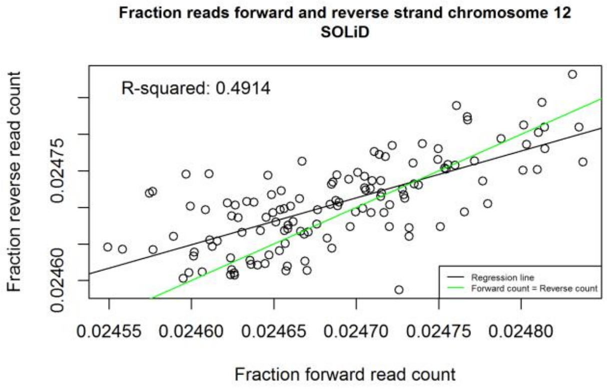

m1

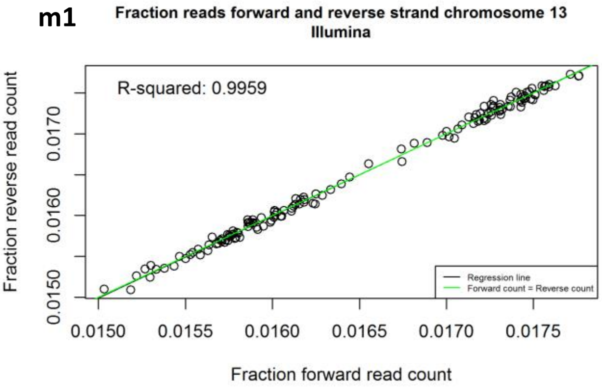

m2

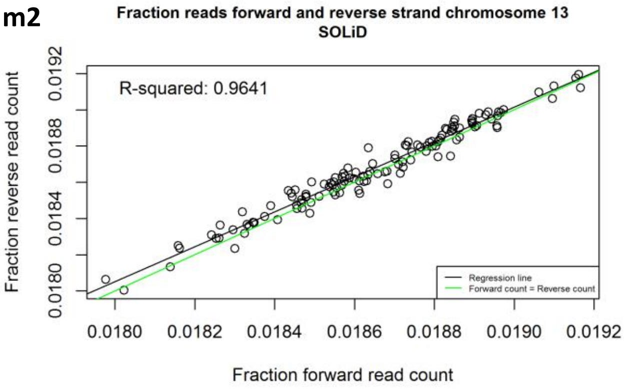

n1

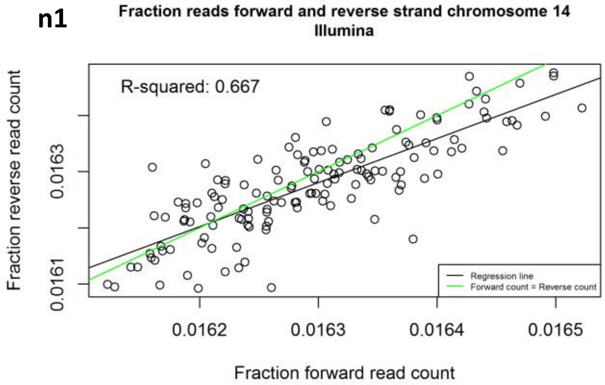

n2

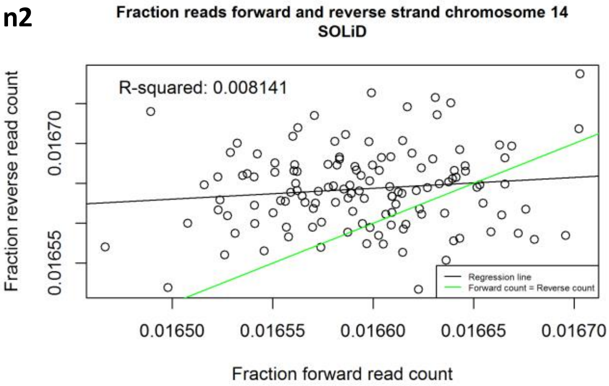

o1

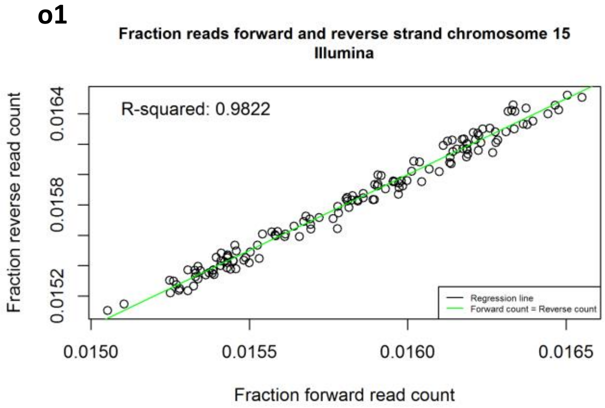

o2

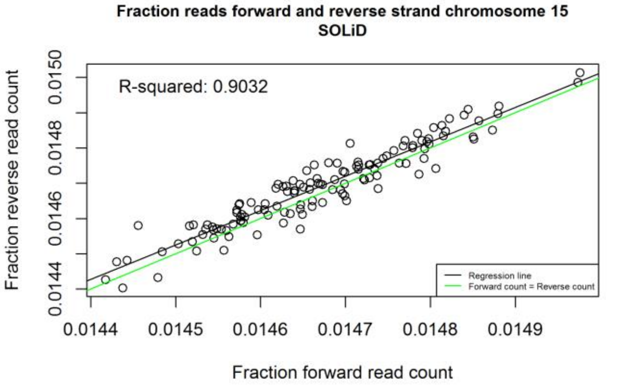

p1

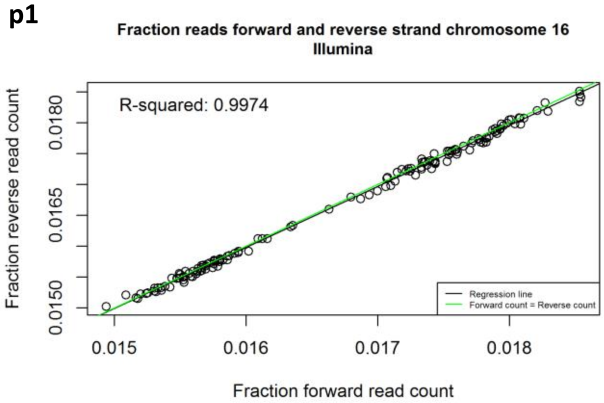

p2

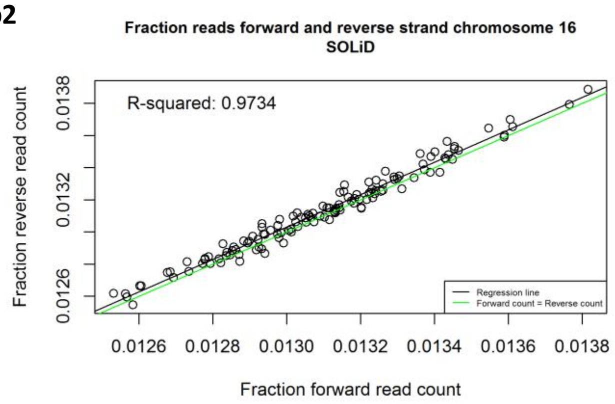

q1

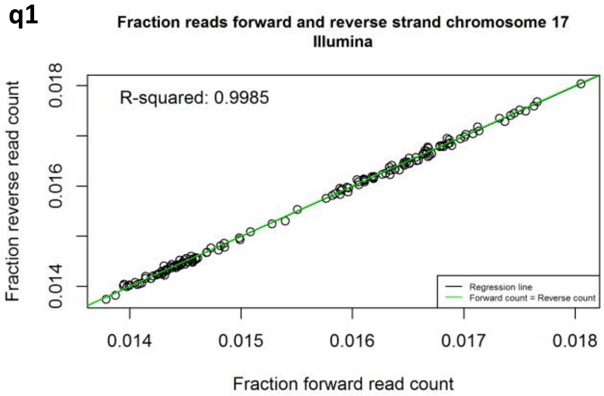

q2

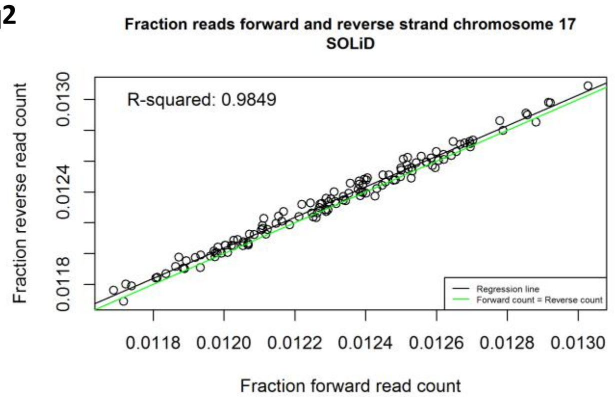

r1

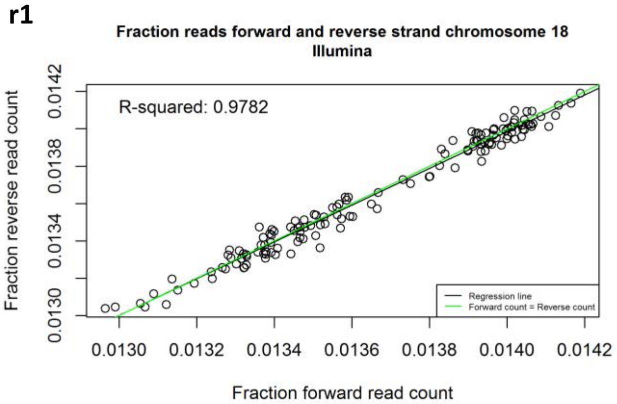

r2

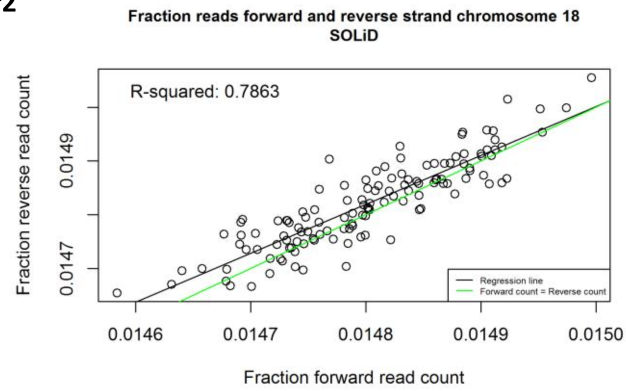

s1

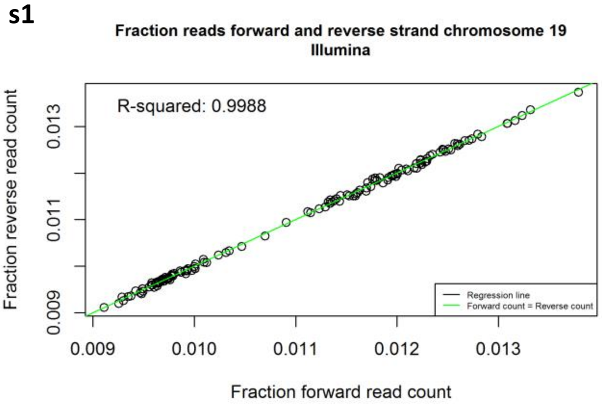

s2

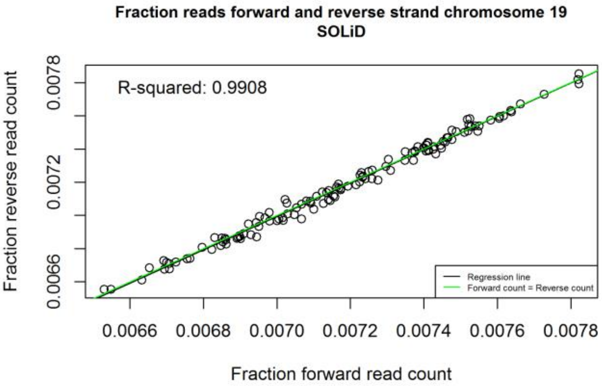

t1

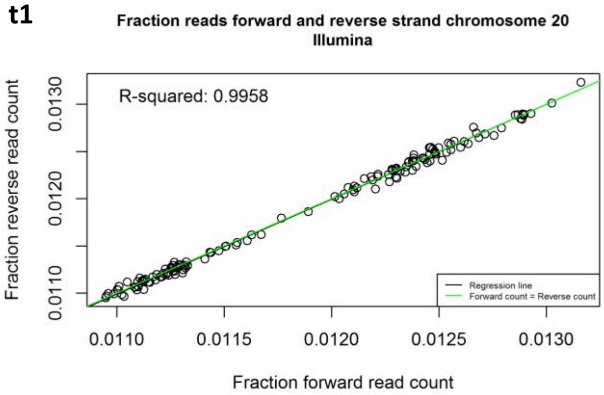

t2

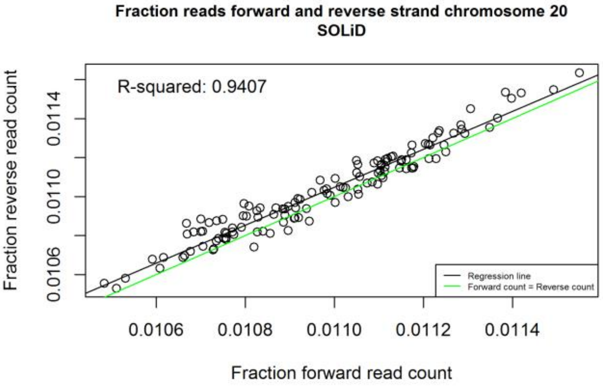

u1

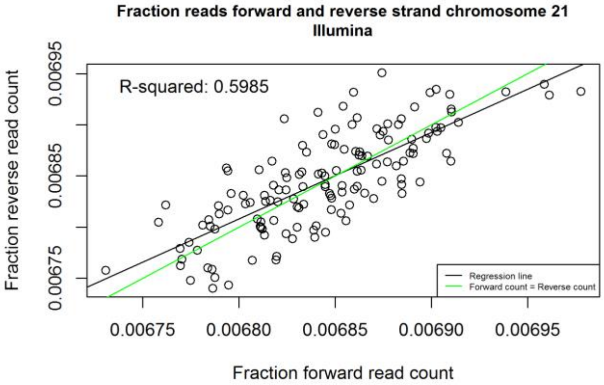

u2

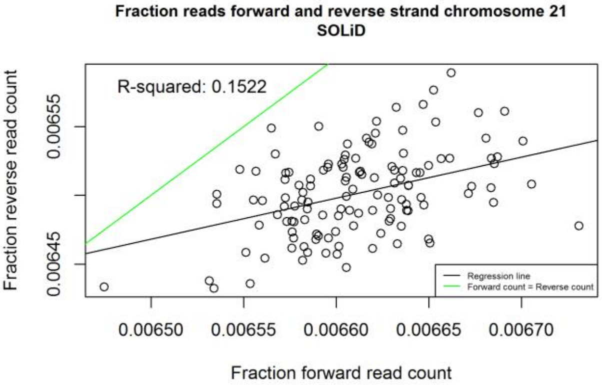

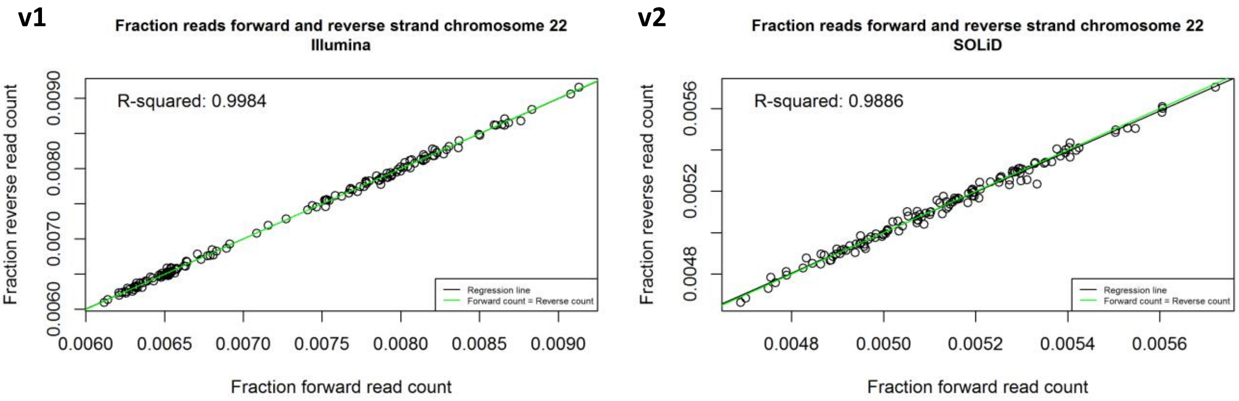

Figure S2.1: Regression models of the fraction of uncorrected read counts aligned to the reverse strand on the fraction aligned to the forward strand for all chromosomes of both the 142 Illumina control samples (1) and the 128 SOLiD control samples (2). (a-v) chromosomes 1-22.

Consistent over- or under-representation of reads aligned to the forward or to the reverse strand leads to skewing of the difference in read counts towards one of the strands. Statistics of the distribution of the differences are shown in **Table S2.2** and plots of these distributions for all chromosomes are shown in **Figure S2.2.**

	Illumina				SOLiD			
			differences F - R				differences F - R	
	total F > R (%)	total R > F (%)	Shapiro Wilk p-value	skewness	total F > R (%)	total R > F (%)	Shapiro Wilk p-value	skewness
chr1	38.0	62.0	0.97	0.11	78.9	21.1	0.37	0.26
chr2	40.8	59.2	0.38	0.26	68.8	31.3	0.37	0.00
chr3	43.0	57.0	0.36	0.18	78.1	21.9	0.20	-0.30
chr4	42.3	57.7	0.02	0.51	96.1	3.9	0.95	0.04
chr5	40.1	59.9	0.97	0.03	77.3	22.7	0.37	-0.29
chr6	46.5	53.5	0.09	-0.39	59.4	40.6	0.03	-0.45
chr7	54.2	45.8	0.44	-0.03	39.1	60.9	0.05	0.30
chr8	42.3	57.7	0.50	-0.21	36.7	63.3	0.69	0.20
chr9	54.9	45.1	0.66	-0.14	40.6	59.4	0.33	-0.27
chr10	48.6	51.4	0.51	0.15	75.0	25.0	0.64	-0.09
chr11	49.3	50.7	0.28	0.16	71.9	28.1	0.55	-0.18
chr12	53.5	46.5	0.60	0.04	61.7	38.3	0.23	0.14
chr13	48.6	51.4	0.30	0.22	72.7	27.3	0.53	-0.19
chr14	29.6	70.4	0.38	-0.12	79.7	20.3	0.56	-0.08
chr15	47.9	52.1	0.75	0.03	72.7	27.3	0.16	-0.21
chr16	28.2	71.8	0.65	-0.21	78.1	21.9	0.87	0.07
chr17	37.3	62.7	0.29	0.25	78.9	21.1	0.35	0.09
chr18	41.5	58.5	0.76	-0.07	72.7	27.3	0.74	0.13
chr19	50.7	49.3	0.58	0.11	41.4	58.6	0.98	0.02
chr20	46.5	53.5	0.89	-0.01	79.7	20.3	0.59	0.17
chr21	48.6	51.4	0.03	0.31	0.0	100.0	0.02	-0.54
chr22	48.6	51.4	0.86	0.01	50.8	49.2	0.01	-0.56

Table S2.2 Statistics of the differences in read numbers aligned to the forward and to the reverse strand for all chromosomes of both the Illumina and the SOLiD control group.

a1

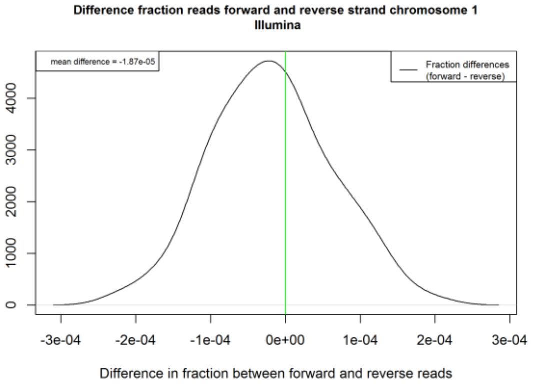

a2

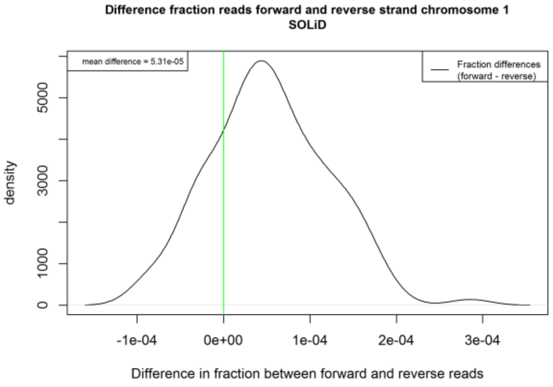

b1

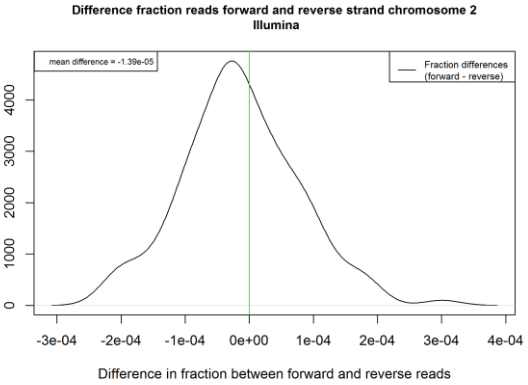

b2

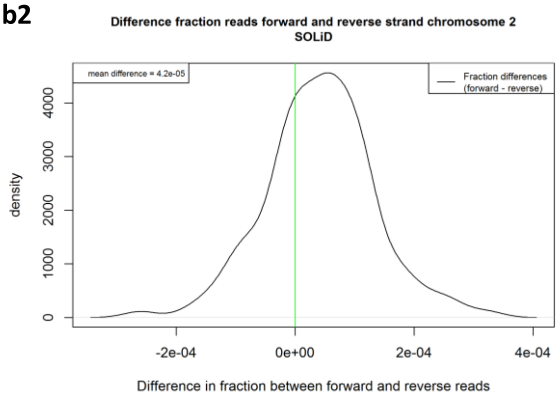

c1

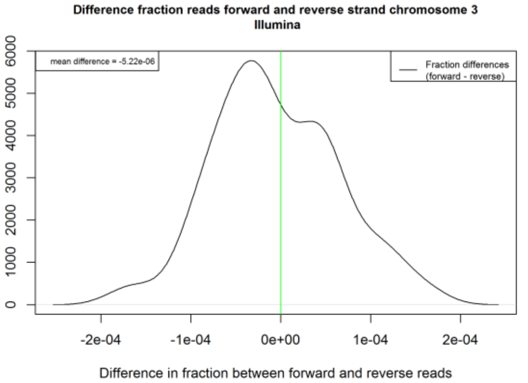

c2

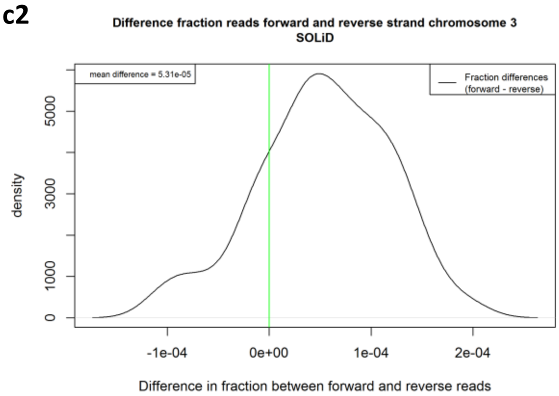

d1

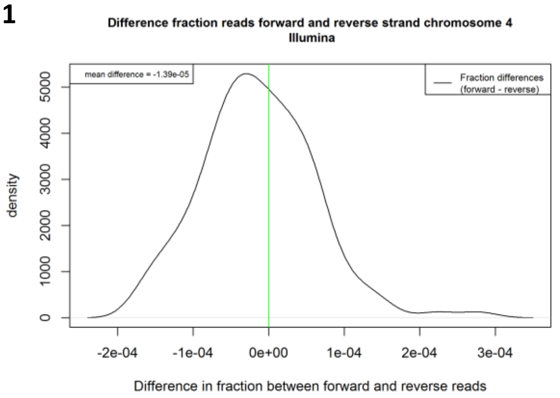

d2

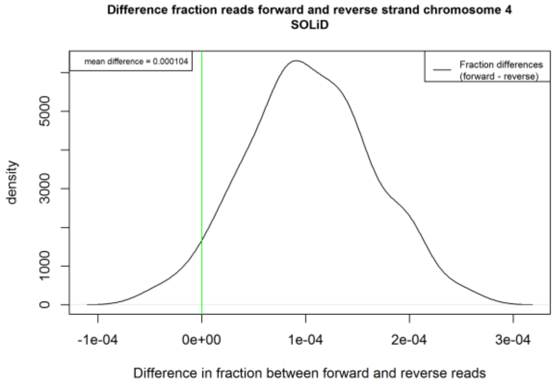

e1

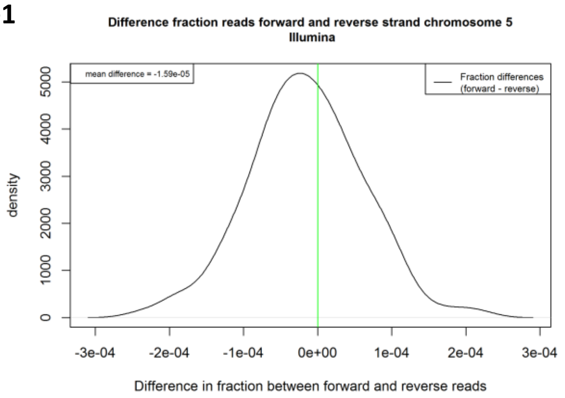

e2

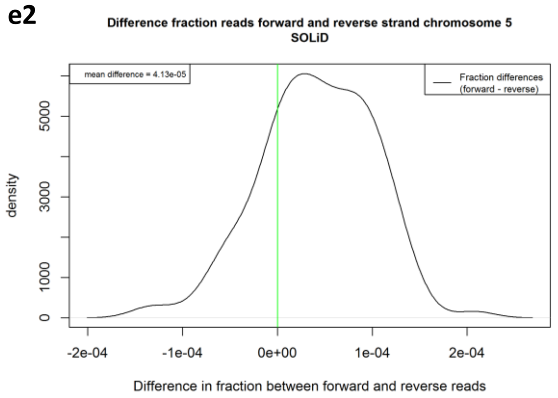

f1

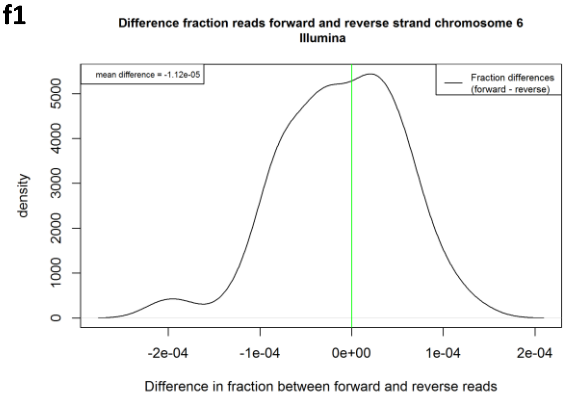

f2

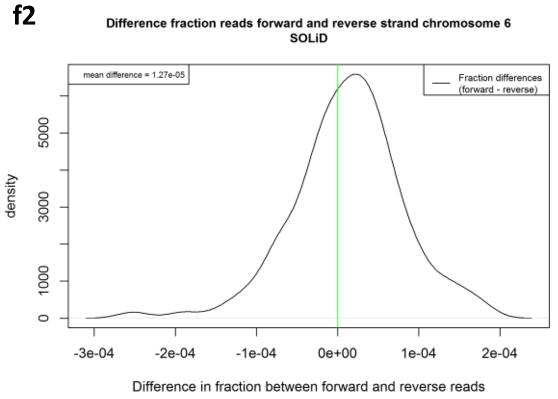

g1

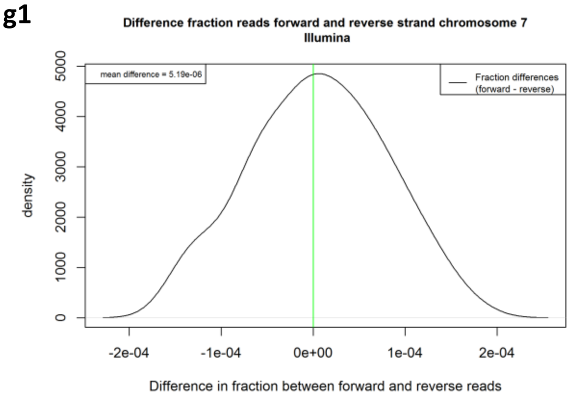

g2

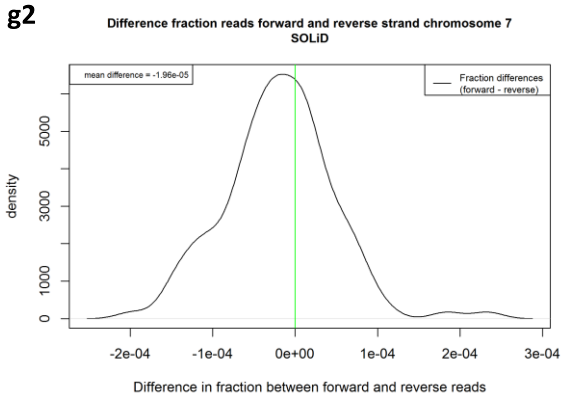

h1

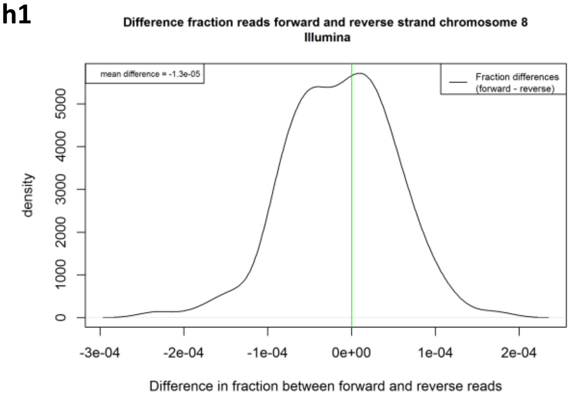

h2

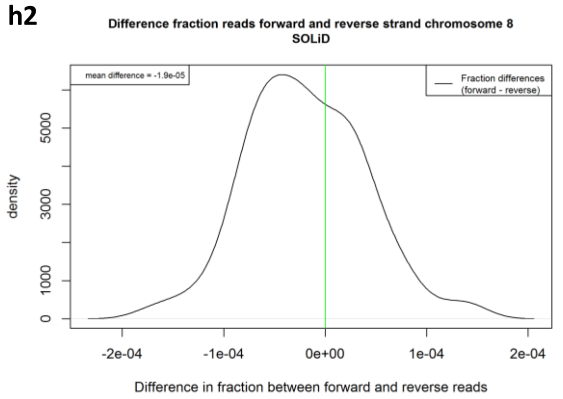

i1

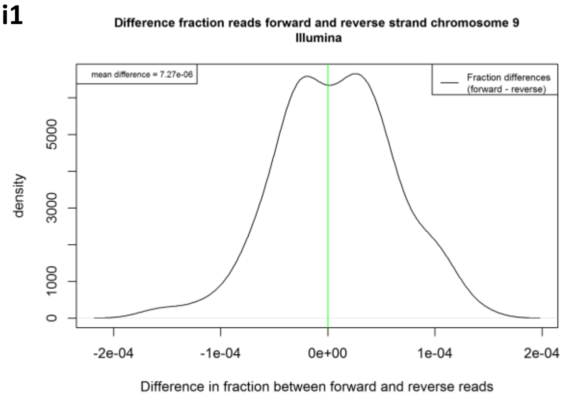

i2

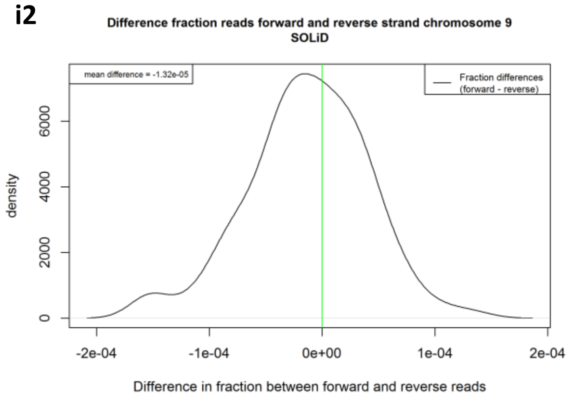

j1

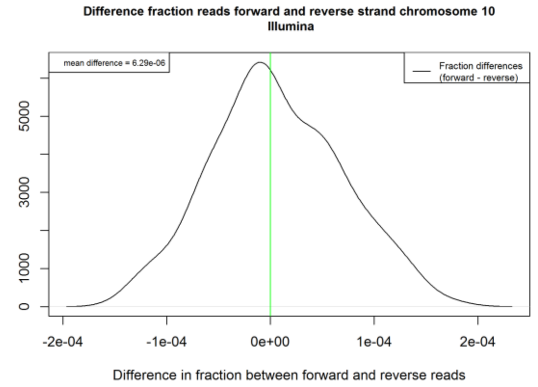

j2

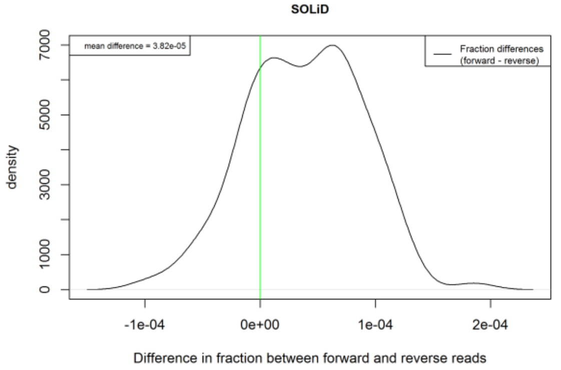

k1

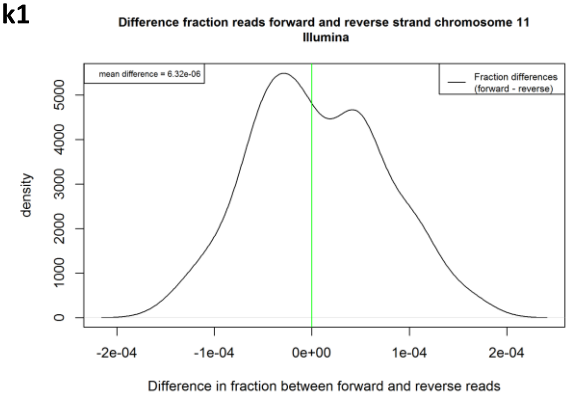

k2

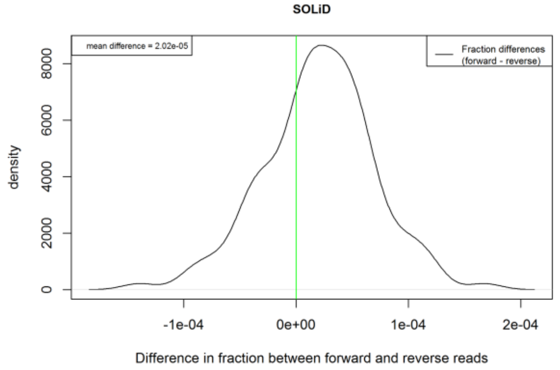

l1

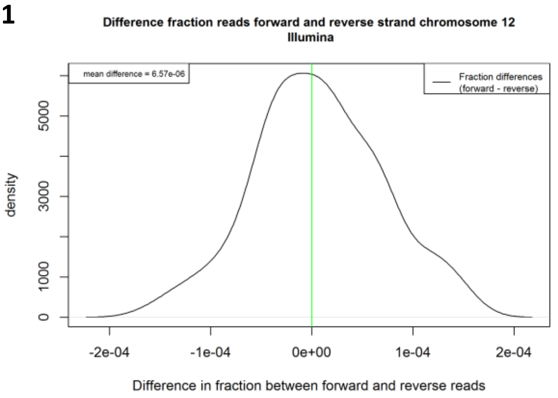

l2

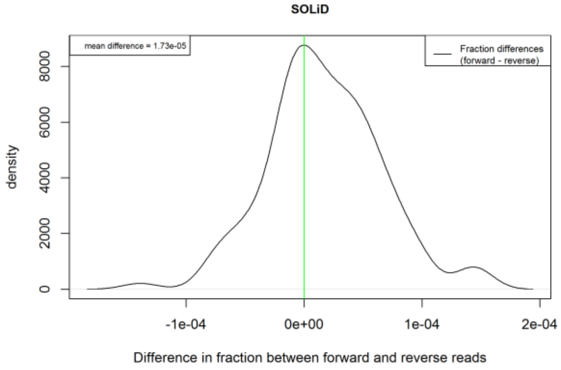

m1

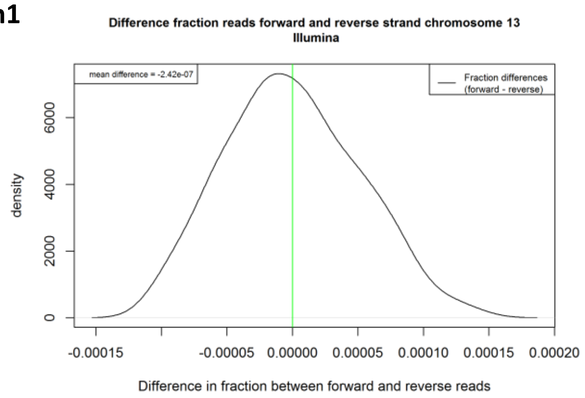

m2

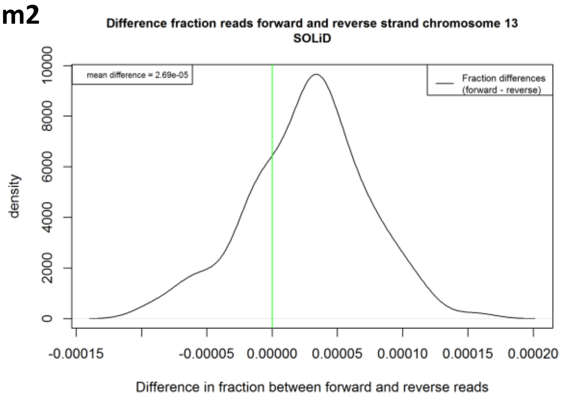

n1

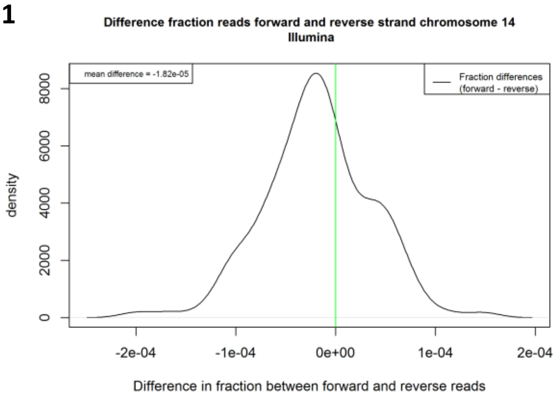

n2

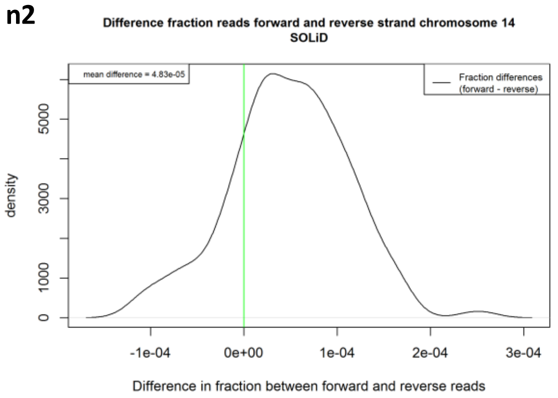

o1

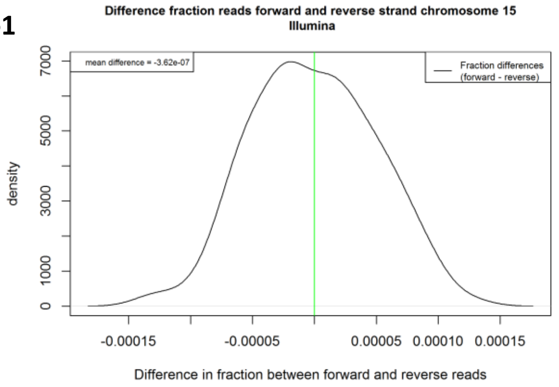

o2

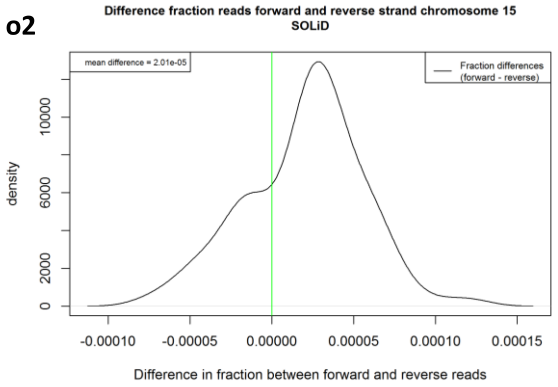

p1

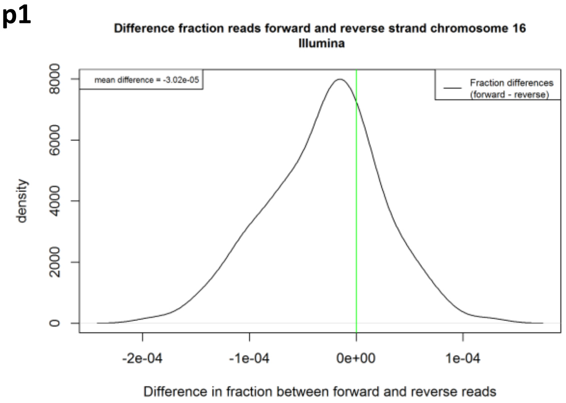

p2

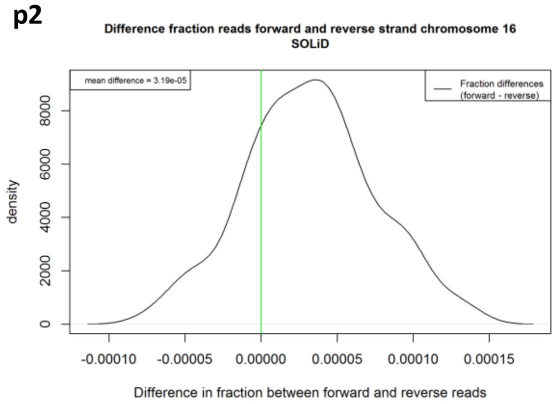

q1

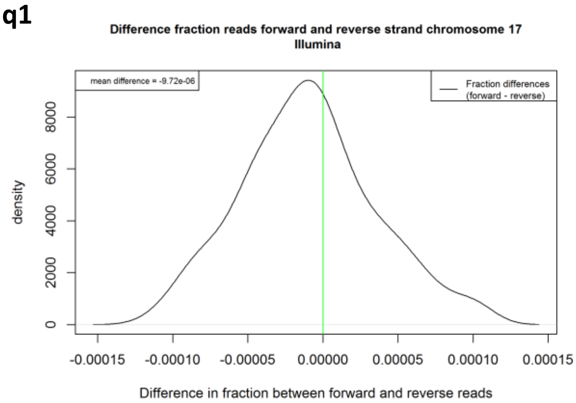

q2

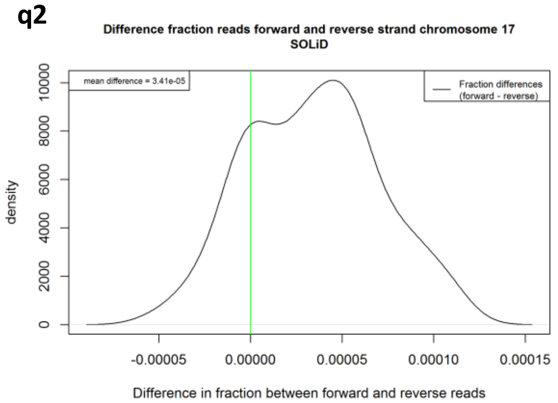

r1

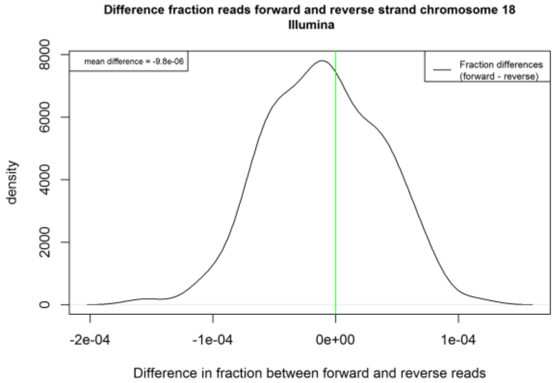

r2

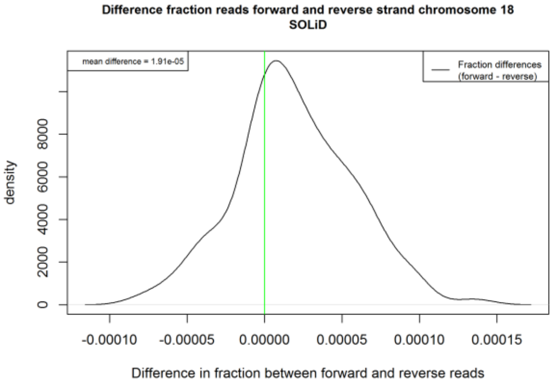

s1

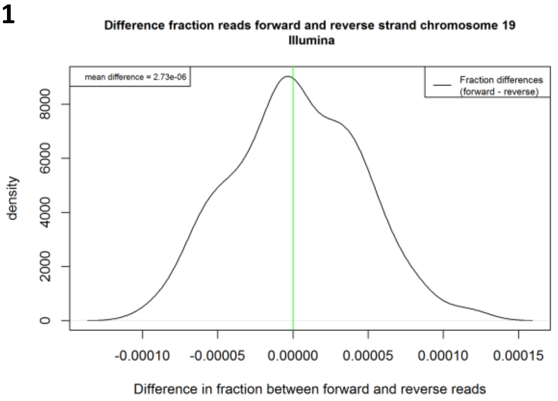

s2

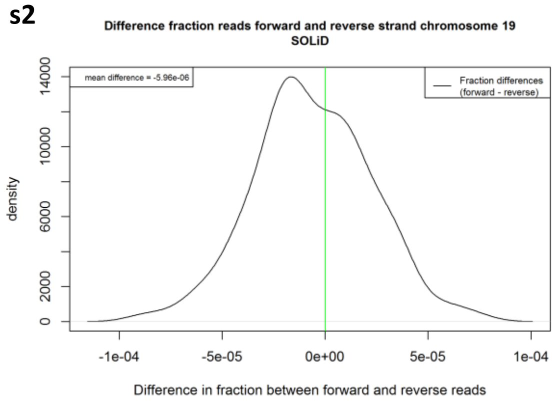

t1

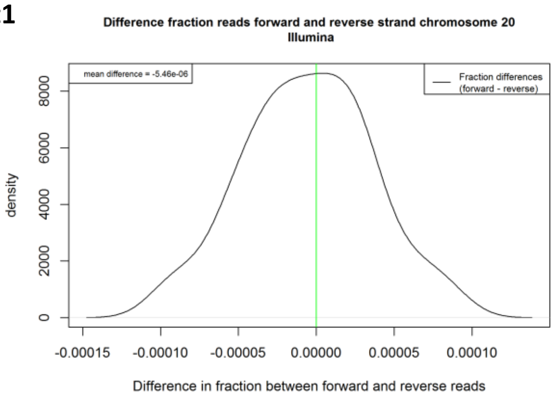

t2

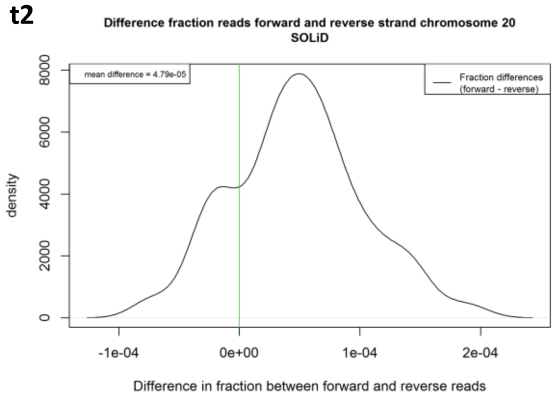

u1

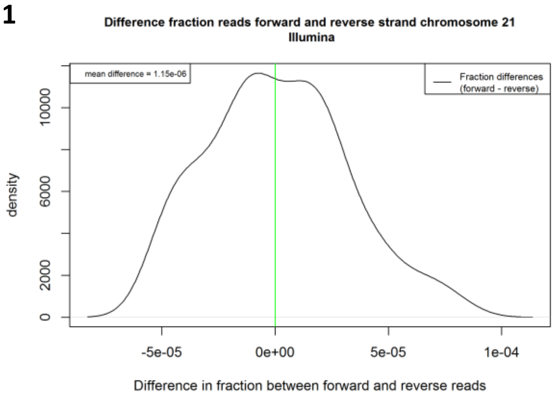

u2

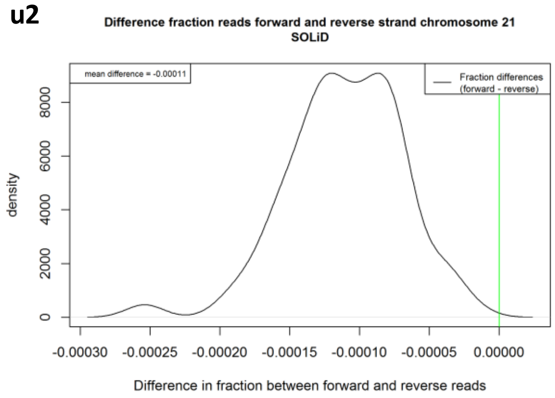

v1

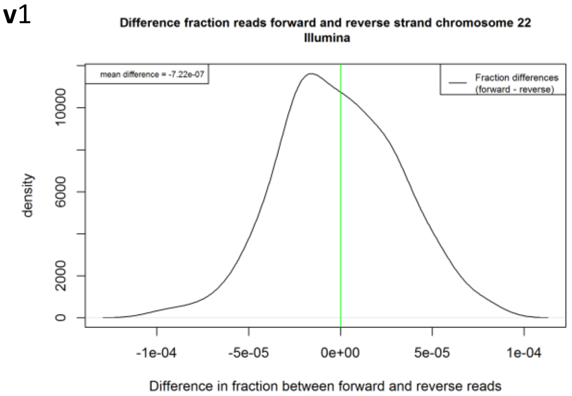

v2

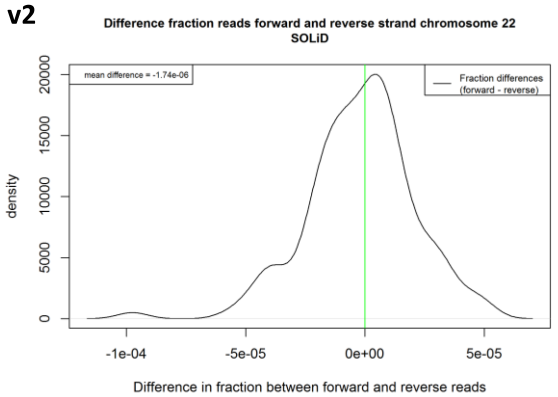

Figure S2.2: Density plots of differences in fractions of uncorrected read counts aligned to the forward and to the reverse strand for all chromosomes of both the 142 Illumina control samples (1) and the 128 sample SOLiD control samples (2). (a-v) chromosomes 1-22.

Supplement 3: Example of regression model for chromosome 13

This supplement contains a series of graphs to visualize an example of a model upon which the regression based Z-score (RBZ) is based. The input of the RBZ model are the chromosomal fractions of the control group samples. Chromosomal fractions of reads aligned to the forward strand and reads aligned to the reverse strand are considered as separate predictors, since there are consistent differences between those fractions (Supplement S2. However, reads aligned to the forward or reverse strand are considered together for the chromosome of interest, because this yields the lowest CV. **Table S3.1** and **figure S3.1** show a regression model using four predictors to predict the expected chromosomal fraction of chromosome 13 based upon the 142 Illumina control samples, without any variation correction.

Coefficients:					
	Estimate	Std. Error	t	value	Pr(>|t|)
Intercept	0.018236	0.004737	3.85	0.00018	***
4F	0.527854	0.056882	9.28	3.36E-16	***
6F	0.391124	0.086029	4.546	1.19E-05	***
16F	-0.20697	0.04596	-4.503	1.42E-05	***
1F	-0.25465	0.067397	-3.778	0.000235	***
*** significance <0.001					

Table S3.1 Coefficients of regression model chromosome 13 Illumina

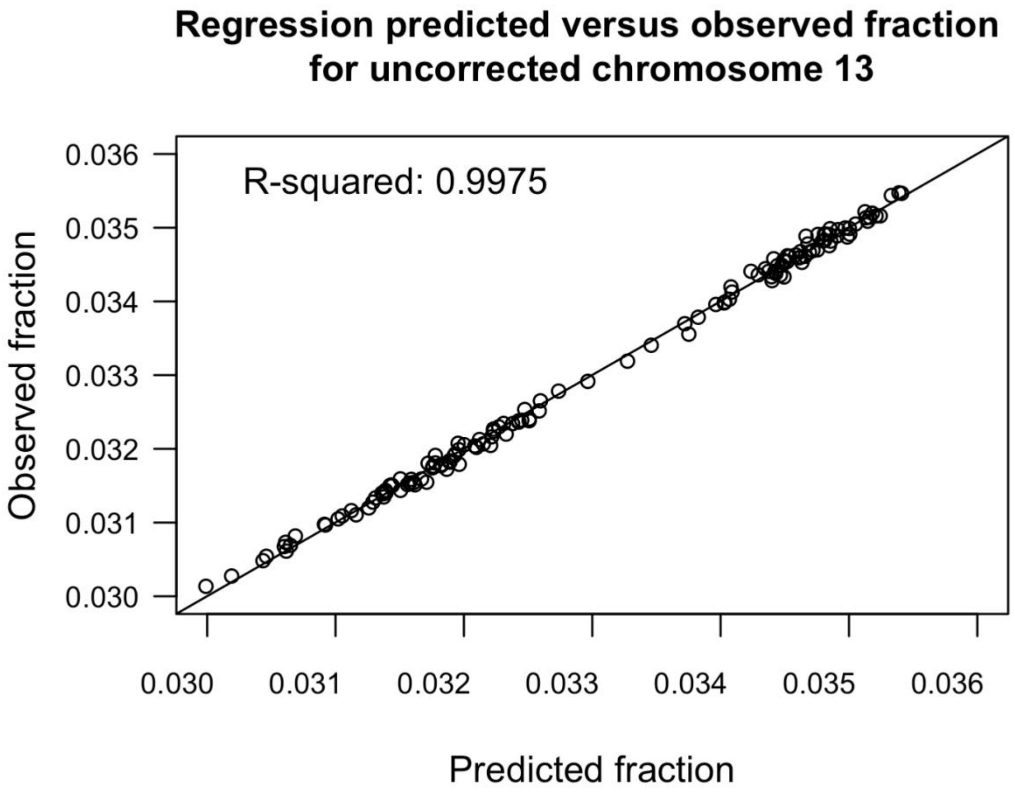

Figure S3.1: Regression model for prediction of expected read count for chromosome 13 based upon uncorrected Illumina control group samples

The four predictors in the regression model are selected using stepwise regression with forward selection. Which predictors are selected depends on the control group. For the 142 Illumina control samples, the best predictors were reads aligned to the forward strands of chromosomes 1, 4, 6 and 16. The reads aligned to chromosomes 4 and 6 showed a positive correlation with the number of reads on chromosome 13, while the reads aligned to chromosomes 1 and 16 showed a negative correlation **(Figure S3.2).**The read counts in the graphs are normalized by dividing them by the mean read count of the sample and multiplying them by the average mean read count of all control samples.

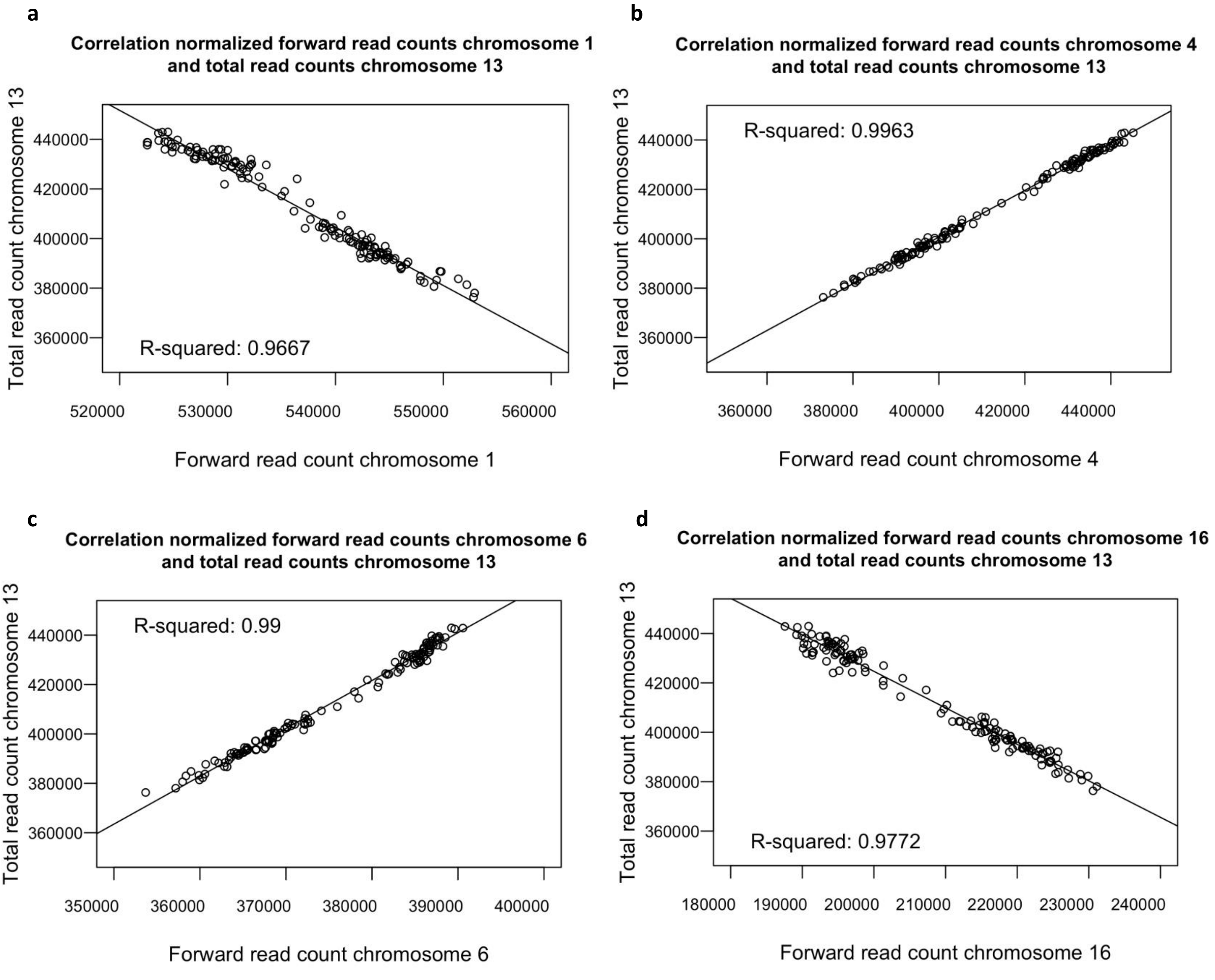

Figure S3.2: Correlation between normalized read counts of predictor chromosomes and normalized read counts on chromosome 13 for 142 Illumina control samples. (a) Chromosome 1, **(b)** chromosome 4, **(c)** chromosome 6 and **(d)** chromosome 16.

The predicted chromosomal fraction is equal to the expected chromosomal fraction in a non-trisomy situation (*ef*). For each sample a ratio between predicted and observed chromosomal fraction is calculated, resulting in a ratio observed/predicted fraction (*of/ef*) **(Figure S3.3a)**. Using

these values a Z-score can be calculated for each sample **(Figure S3.3b)**. The general structure of the formula is equal to the standard Z-score formula:

$$Z = \frac{x - \mu}{\sigma}$$

Because the mean of the control group after regression is one, the coefficient of variation of the control group has the same value as the SD. Using the same structure, the RBZ can be formulated as:

$$RBZ = \frac{{}^{of_s}/_{ef_s} - 1}{\sqrt{\sum_{j=1}^{n}\left(of_j/ef_j - \overline{of/ef}\,\right)^2 \Big/ n-1}}$$

Where s is the sample of interest, j is an individual control sample and n is the total number of control samples. The regression model for trisomy prediction for chromosome 13 in uncorrected Illumina data, described in table S3.1, resulted in a mean fraction of 1.0000 and a CV of 0.0024 (0.24%).

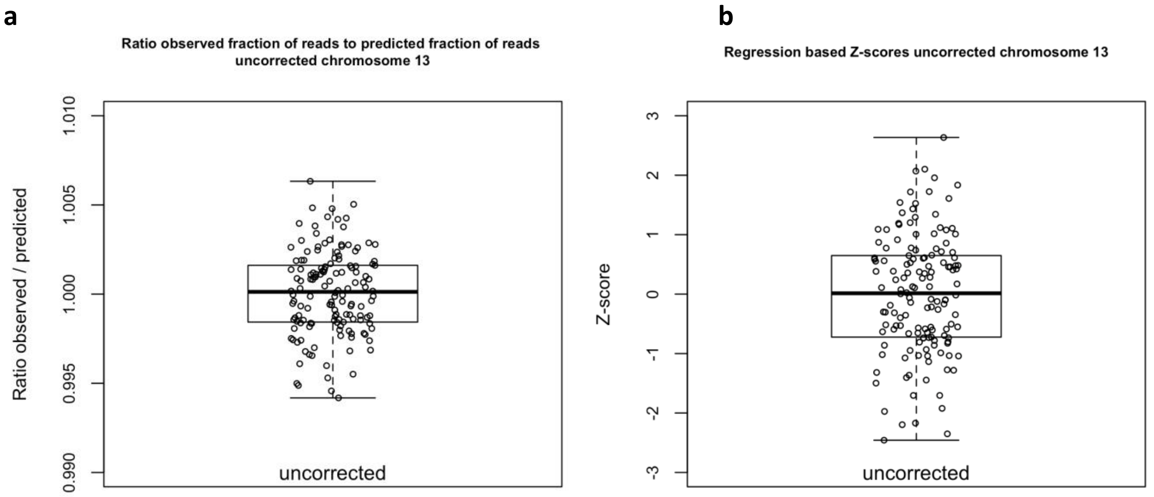

Figure S3.3: Ratios observed / predicted and Z-scores for chromosome 13 for 142 uncorrected Illumina control samples. (a) ratios observed / predicted **(b)** Z-scores.

The number of predictors used in the RBZ can be as low as one or as high as all autosomes. However, we advise using a minimum of four predictor chromosomes, since an aberration in one of the other chromosomes (in mother or child) could influence the prediction. The effect of such an aberration is larger when fewer predictors are used. For the same reason we advise not using both the reads aligned to the forward strand and reads aligned to the reverse strand of the same chromosome in the same model.

Different independent RBZ models can be created for each analysis. We advise creating four different models, because reads originating from the same chromosome can be included in a maximum of two different models. Results affected by an aberration in one of the predictor chromosomes can be identified using the additional models.

Supplement 4: Relation between coefficient of variation, percentage cell free fetal DNA and sensitivity

This supplement shows the relation between the coefficient of variation (CV), the percentage of cell free fetal DNA (cffDNA) and the sensitivity.

Together the CV and the percentage cffDNA determine the expected Z-score for a specific sample. The expected Z-score can be calculated using the following formula:

$$Zexp = \frac{0.5 * cffDNA}{CV}$$

In the formula cffDNA and CV are either both fractions or both percentages (fraction * 100). Using this formula it is easy to see that a higher percentage cffDNA or a lower CV result in a higher expected Z-score in the case of a trisomy. This expected Z-score is the mean of a normal distribution, which means that at a Z-expected of three, half of the analyses have a Z-score lower than three. A robust analysis in which most positive samples are expected to be detected, requires an expected Z-score at least three standard deviations (SD) above the threshold. Thus, at a threshold of three, a Z-expected of six is required for a 99.9% sensitivity. **Table S4.1** shows a series of combinations of percentages cffDNA and CVs and the resulting expected Z-scores and expected sensitivities.

CV (%)	cffDNA %	Zexp	Sensitivity (%) (threshold Z=3)
0.150	2	6.7	100
0.150	3	10.0	100
0.150	4	13.3	100
0.175	2	5.7	99.7
0.175	3	8.6	100
0.175	4	11.4	100
0.200	2	5.0	97.7
0.200	3	7.5	100
0.200	4	10.0	100
0.225	2	4.4	92.5
0.225	3	6.7	100
0.225	4	8.9	100
0.250	2	4.0	84.1
0.250	3	6.0	99.9
0.250	4	8.0	100

CV (%)	cffDNA %	Zexp	Sensitivity (%) (threshold Z=3)
0.300	2	3.3	62.9
0.300	3	5.0	97.7
0.300	4	6.7	100
0.350	2	2.9	44.4
0.350	3	4.3	90.1
0.350	4	5.7	99.7
0.400	2	2.5	30.9
0.400	3	3.8	77.3
0.400	4	5.0	97.7
0.450	2	2.2	21.8
0.450	3	3.3	62.9
0.450	4	4.4	92.5
0.500	2	2.0	15.9
0.500	3	3.0	50.0
0.500	4	4.0	84.1

Table S4.1 Relation between coefficient of variation, percentage cffDNA, expected Z-score and theoretical sensitivity, based on a threshold for trisomy calling of Z=3.

The minimum theoretical possible CV is equal to the square root of the number of independent reads present on the chromosome of interest. The minimum theoretical possible CV's for the Illumina and SOLiD samples analyzed are listed in **table S4.2.**

	bp	fraction	read count		theoretical CV (%)	
			Illumina	SOLiD	Illumina	SOLiD
total autosomes	2,881,033,286	1	12500000	20200000		
13	115,169,878	0.040	499690	807499	0.14	0.11
18	78,077,248	0.027	338755.4	547428.7	0.17	0.14
21	48,129,895	0.017	208822.2	337456.7	0.22	0.17

Table S4.2 theoretical possible coefficient of variation for chromosomes 13, 18 and 21 based on the mean autosomal read count of the analyzed Illumina and SOLiD samples.

Due to overdispersion (the presence of a greater variability than would be expected by chance) the practical CV will always be higher than the minimum theoretical possible CV.

Supplement 5: Coefficients of variation for all combinations of variation reduction and trisomy prediction methods and their effect on sensitivity and specificity

CV(%)		Chr13				Chr18				Chr21			
		Z-score	MAD	NCV	RBZ	Z-score	MAD	NCV	RBZ	Z-score	MAD	NCV	RBZ
SOLiD	Weighted bin GC and chi corrected	0.21	0.18	0.20	0.19	0.28	0.31	0.21	0.18	0.33	0.32	0.29	0.26
	Weighted bin GC corrected	0.24	0.23	0.21	0.20	0.35	0.38	0.23	0.20	0.45	0.46	0.42	0.40
	LOESS GC and chi corrected	0.21	0.18	0.20	0.19	0.27	0.29	0.20	0.18	0.33	0.33	0.30	0.26
	LOESS GC corrected	0.24	0.23	0.21	0.20	0.35	0.36	0.23	0.20	0.46	0.51	0.42	0.40
	Peak and chi corrected	0.82	0.87	0.23	0.20	0.35	0.39	0.21	0.19	0.38	0.35	0.33	0.29
	Peak corrected	1.28	1.32	0.31	0.23	0.52	0.57	0.22	0.20	0.41	0.35	0.35	0.30
	Peak, weighted bin GC and chi corrected	0.21	0.19	0.20	0.18	0.28	0.30	0.20	0.18	0.33	0.31	0.29	0.26
	Peak and weighted bin GC corrected	0.23	0.20	0.21	0.20	0.35	0.35	0.22	0.19	0.35	0.29	0.32	0.30
	Peak, LOESS GC and chi corrected	0.21	0.18	0.20	0.18	0.27	0.28	0.20	0.18	0.33	0.31	0.30	0.26
	Peak and LOESS GC corrected	0.23	0.20	0.21	0.19	0.34	0.36	0.22	0.20	0.35	0.32	0.33	0.30
	Chi corrected	0.81	0.86	0.23	0.20	0.36	0.38	0.20	0.19	0.38	0.34	0.32	0.29
	Uncorrected	1.28	1.30	0.31	0.23	0.53	0.57	0.22	0.20	0.47	0.47	0.44	0.42
Illumina	Weighted bin GC and chi corrected	0.22	0.24	0.21	0.18	0.31	0.29	0.26	0.22	0.35	0.36	0.33	0.31
	Weighted bin GC corrected	0.25	0.23	0.22	0.20	0.30	0.31	0.26	0.24	0.50	0.45	0.47	0.43
	LOESS GC and chi corrected	0.22	0.24	0.20	0.17	0.31	0.30	0.26	0.22	0.35	0.35	0.33	0.31
	LOESS GC corrected	0.24	0.24	0.21	0.19	0.32	0.34	0.27	0.24	0.51	0.47	0.47	0.44
	Peak and chi corrected	1.77	2.33	0.26	0.20	0.55	0.60	0.27	0.24	0.44	0.48	0.40	0.37
	Peak corrected	4.69	6.51	0.67	0.23	2.31	3.22	0.28	0.24	0.58	0.62	0.43	0.40
	Peak, weighted bin GC and chi corrected	0.22	0.24	0.21	0.19	0.31	0.30	0.26	0.23	0.34	0.34	0.33	0.30
	Peak and weighted bin GC corrected	0.23	0.25	0.20	0.19	0.30	0.30	0.26	0.24	0.39	0.35	0.38	0.35
	Peak, LOESS GC and chi corrected	0.22	0.23	0.20	0.17	0.31	0.31	0.27	0.23	0.35	0.35	0.33	0.31
	Peak and LOESS GC corrected	0.22	0.24	0.20	0.19	0.31	0.30	0.27	0.24	0.40	0.34	0.38	0.35
	Chi corrected	1.79	2.36	0.26	0.20	0.55	0.59	0.27	0.24	0.44	0.46	0.41	0.37
	Uncorrected	4.76	6.69	0.38	0.24	2.31	3.29	0.28	0.25	0.64	0.65	0.50	0.44

Table S5.1 Coefficients of variation in percentage (CV(%)) for all combinations of variation reduction and trisomy prediction methods.

Using the coefficients of variation described in table S5.1 the theoretical sensitivity was calculated for samples having a percentage of cell free fetal DNA of 2%, 3%, 4% and 5% based on thresholds of Z-score 2, 3 and 4, reflecting specificities of 97,72%, 99,87% and 100% assuming normal distributions. Based on the formula described in supplement 4, using the percentage of cell free fetal DNA and the CV of the specific method the expected Z-scores were calculated. Assuming a normal distribution around the expected Z-scores the sensitivity was calculated by using the distances between the expected Z-scores and the used thresholds. For instance an expected Z-score of 5 and a threshold of Z = 2, results in a difference of three standard-deviations, resulting in a sensitivity of 99,87% and an expected Z-score of 1 would lead to a difference of -1 standard deviation compared to the threshold, resulting in a sensitivity of 15,87%. These values are shown in **tables s5.2 to S5.13**.

sensitivity at 2% cffDNA<br>Threshold Z=2		Chr13				Chr18				Chr21			
		Z-score	MAD	NCV	RBZ	Z-score	MAD	NCV	RBZ	Z-score	MAD	NCV	RBZ
SOLiD	Weighted bin GC and chi corrected	0.9974	0.9998	0.9987	0.9995	0.9452	0.8849	0.9974	0.9998	0.8159	0.8643	0.9192	0.9641
	Weighted bin GC corrected	0.9861	0.9893	0.9974	0.9987	0.8159	0.7257	0.9893	0.9987	0.5793	0.5793	0.6554	0.6915
	LOESS GC and chi corrected	0.9974	0.9998	0.9987	0.9995	0.9554	0.9192	0.9987	0.9998	0.8159	0.8159	0.9032	0.9641
	LOESS GC corrected	0.9861	0.9893	0.9974	0.9987	0.8159	0.7881	0.9893	0.9987	0.5793	0.5000	0.6554	0.6915
	Peak and chi corrected	0.2119	0.1841	0.9893	0.9987	0.8159	0.7257	0.9974	0.9995	0.7257	0.8159	0.8159	0.9192
	Peak corrected	0.1151	0.1151	0.8849	0.9893	0.4602	0.4207	0.9938	0.9987	0.6554	0.8159	0.8159	0.9032
	Peak, weighted bin GC and chi corrected	0.9974	0.9995	0.9987	0.9998	0.9452	0.9032	0.9987	0.9998	0.8159	0.8849	0.9192	0.9641
	Peak and weighted bin GC corrected	0.9893	0.9987	0.9974	0.9987	0.8159	0.8159	0.9938	0.9995	0.8159	0.9192	0.8643	0.9032
	Peak, LOESS GC and chi corrected	0.9974	0.9998	0.9987	0.9998	0.9554	0.9452	0.9987	0.9998	0.8159	0.8849	0.9032	0.9641
	Peak and LOESS GC corrected	0.9893	0.9987	0.9974	0.9995	0.8159	0.7881	0.9938	0.9987	0.8159	0.8643	0.8159	0.9032
	Chi corrected	0.2119	0.2119	0.9893	0.9987	0.7881	0.7257	0.9987	0.9995	0.7257	0.8159	0.8643	0.9192
	Uncorrected	0.1151	0.1151	0.8849	0.9893	0.4602	0.4207	0.9938	0.9987	0.5398	0.5398	0.6179	0.6554
Illumina	Weighted bin GC and chi corrected	0.9938	0.9861	0.9974	0.9998	0.8849	0.9192	0.9641	0.9938	0.8159	0.7881	0.8159	0.8849
	Weighted bin GC corrected	0.9772	0.9893	0.9938	0.9987	0.9032	0.8849	0.9641	0.9861	0.5000	0.5793	0.5398	0.6179
	LOESS GC and chi corrected	0.9938	0.9861	0.9987	1.0000	0.8849	0.9032	0.9641	0.9938	0.8159	0.8159	0.8159	0.8849
	LOESS GC corrected	0.9861	0.9861	0.9974	0.9995	0.8643	0.8159	0.9554	0.9861	0.5000	0.5398	0.5398	0.6179
	Peak and chi corrected	0.0808	0.0548	0.9641	0.9987	0.4207	0.3821	0.9554	0.9861	0.6179	0.5398	0.6915	0.758
	Peak corrected	0.0359	0.0359	0.3085	0.9893	0.0548	0.0446	0.9452	0.9861	0.3821	0.3446	0.6179	0.6915
	Peak, weighted bin GC and chi corrected	0.9938	0.9861	0.9974	0.9995	0.8849	0.9032	0.9641	0.9893	0.8159	0.8159	0.8159	0.9032
	Peak and weighted bin GC corrected	0.9893	0.9772	0.9987	0.9995	0.9032	0.9032	0.9641	0.9861	0.7257	0.8159	0.7257	0.8159
	Peak, LOESS GC and chi corrected	0.9938	0.9893	0.9987	1.0000	0.8849	0.8849	0.9554	0.9893	0.8159	0.8159	0.8159	0.8849
	Peak and LOESS GC corrected	0.9938	0.9861	0.9987	0.9995	0.8849	0.9032	0.9554	0.9861	0.6915	0.8159	0.7257	0.8159
	Chi corrected	0.0808	0.0548	0.9641	0.9987	0.4207	0.3821	0.9554	0.9861	0.6179	0.5793	0.6554	0.758
	Uncorrected	0.0359	0.0287	0.7257	0.9861	0.0548	0.0446	0.9452	0.9772	0.3446	0.3085	0.5000	0.6179

Table S5. 2 Theoretical sensitivities for trisomy detection for all combinations of variation reduction and prediction algorithms at a threshold of Z=2 (specificity = 97,72%) in case of a percentage of fetal DNA of 2%.

sensitivity at 2% cffDNA<br>Threshold Z=3		Chr13				Chr18				Chr21			
		Z-score	MAD	NCV	RBZ	Z-score	MAD	NCV	RBZ	Z-score	MAD	NCV	RBZ
SOLiD	Weighted bin GC and chi corrected	0.9641	0.9953	0.9772	0.9893	0.7257	0.5793	0.9641	0.9953	0.5000	0.5398	0.6554	0.7881
	Weighted bin GC corrected	0.8849	0.9032	0.9641	0.9772	0.4602	0.3446	0.9032	0.9772	0.2119	0.2119	0.2743	0.3085
	LOESS GC and chi corrected	0.9641	0.9953	0.9772	0.9893	0.758	0.6554	0.9772	0.9953	0.5000	0.5000	0.6179	0.7881
	LOESS GC corrected	0.8849	0.9032	0.9641	0.9772	0.4602	0.4207	0.9032	0.9772	0.2119	0.1587	0.2743	0.3085
	Peak and chi corrected	0.0359	0.0287	0.9032	0.9772	0.4602	0.3446	0.9641	0.9893	0.3446	0.4602	0.5000	0.6554
	Peak corrected	0.0139	0.0139	0.5793	0.9032	0.1357	0.1151	0.9332	0.9772	0.2743	0.4602	0.4602	0.6179
	Peak, weighted bin GC and chi corrected	0.9641	0.9893	0.9772	0.9953	0.7257	0.6179	0.9772	0.9953	0.5000	0.5793	0.6554	0.7881
	Peak and weighted bin GC corrected	0.9032	0.9772	0.9641	0.9772	0.4602	0.4602	0.9332	0.9893	0.4602	0.6554	0.5398	0.6179
	Peak, LOESS GC and chi corrected	0.9641	0.9953	0.9772	0.9953	0.758	0.7257	0.9772	0.9953	0.5000	0.5793	0.6179	0.7881
	Peak and LOESS GC corrected	0.9032	0.9772	0.9641	0.9893	0.4602	0.4207	0.9332	0.9772	0.4602	0.5398	0.5000	0.6179
	Chi corrected	0.0359	0.0359	0.9032	0.9772	0.4207	0.3446	0.9772	0.9893	0.3446	0.4602	0.5398	0.6554
	Uncorrected	0.0139	0.0139	0.5793	0.9032	0.1357	0.1151	0.9332	0.9772	0.1841	0.1841	0.242	0.2743
Illumina	Weighted bin GC and chi corrected	0.9332	0.8849	0.9641	0.9953	0.5793	0.6554	0.7881	0.9332	0.4602	0.4207	0.5000	0.5793
	Weighted bin GC corrected	0.8159	0.9032	0.9332	0.9772	0.6179	0.5793	0.7881	0.8849	0.1587	0.2119	0.1841	0.242
	LOESS GC and chi corrected	0.9332	0.8849	0.9772	0.9981	0.5793	0.6179	0.7881	0.9332	0.4602	0.4602	0.5000	0.5793
	LOESS GC corrected	0.8849	0.8849	0.9641	0.9893	0.5398	0.4602	0.758	0.8849	0.1587	0.1841	0.1841	0.242
	Peak and chi corrected	0.0082	0.0047	0.7881	0.9772	0.1151	0.0968	0.758	0.8849	0.242	0.1841	0.3085	0.3821
	Peak corrected	0.0026	0.0026	0.0668	0.9032	0.0047	0.0035	0.7257	0.8849	0.0968	0.0808	0.242	0.3085
	Peak, weighted bin GC and chi corrected	0.9332	0.8849	0.9641	0.9893	0.5793	0.6179	0.7881	0.9032	0.4602	0.4602	0.5000	0.6179
	Peak and weighted bin GC corrected	0.9032	0.8159	0.9772	0.9893	0.6179	0.6179	0.7881	0.8849	0.3446	0.4602	0.3446	0.4602
	Peak, LOESS GC and chi corrected	0.9332	0.9032	0.9772	0.9981	0.5793	0.5793	0.758	0.9032	0.4602	0.4602	0.5000	0.5793
	Peak and LOESS GC corrected	0.9332	0.8849	0.9772	0.9893	0.5793	0.6179	0.758	0.8849	0.3085	0.4602	0.3446	0.4602
	Chi corrected	0.0082	0.0047	0.7881	0.9772	0.1151	0.0968	0.758	0.8849	0.242	0.2119	0.2743	0.3821
	Uncorrected	0.0026	0.0019	0.3446	0.8849	0.0047	0.0035	0.7257	0.8159	0.0808	0.0668	0.1587	0.242

Table S5.3: Theoretical sensitivities for trisomy detection for all combinations of variation reduction and prediction algorithms at a threshold of Z=3 (specificity = 99,87%) in case of a percentage of fetal DNA of 2%.

sensitivity at 2% cffDNA<br>Threshold Z=4		Chr13				Chr18				Chr21			
		Z-score	MAD	NCV	RBZ	Z-score	MAD	NCV	RBZ	Z-score	MAD	NCV	RBZ
SOLiD	Weighted bin GC and chi corrected	0.7881	0.9452	0.8159	0.9032	0.3446	0.2119	0.7881	0.9452	0.1587	0.1841	0.2743	0.4207
	Weighted bin GC corrected	0.5793	0.6179	0.7881	0.8159	0.1357	0.0808	0.6179	0.8159	0.0359	0.0359	0.0548	0.0668
	LOESS GC and chi corrected	0.7881	0.9452	0.8159	0.9032	0.3821	0.2743	0.8159	0.9452	0.1587	0.1587	0.242	0.4207
	LOESS GC corrected	0.5793	0.6179	0.7881	0.8159	0.1357	0.1151	0.6179	0.8159	0.0359	0.0228	0.0548	0.0668
	Peak and chi corrected	0.0026	0.0019	0.6179	0.8159	0.1357	0.0808	0.7881	0.9032	0.0808	0.1357	0.1587	0.2743
	Peak corrected	0.0007	0.0007	0.2119	0.6179	0.0179	0.0139	0.6915	0.8159	0.0548	0.1357	0.1357	0.242
	Peak, weighted bin GC and chi corrected	0.7881	0.9032	0.8159	0.9452	0.3446	0.242	0.8159	0.9452	0.1587	0.2119	0.2743	0.4207
	Peak and weighted bin GC corrected	0.6179	0.8159	0.7881	0.8159	0.1357	0.1357	0.6915	0.9032	0.1357	0.2743	0.1841	0.242
	Peak, LOESS GC and chi corrected	0.7881	0.9452	0.8159	0.9452	0.3821	0.3446	0.8159	0.9452	0.1587	0.2119	0.242	0.4207
	Peak and LOESS GC corrected	0.6179	0.8159	0.7881	0.9032	0.1357	0.1151	0.6915	0.8159	0.1357	0.1841	0.1587	0.242
	Chi corrected	0.0026	0.0026	0.6179	0.8159	0.1151	0.0808	0.8159	0.9032	0.0808	0.1357	0.1841	0.2743
	Uncorrected	0.0007	0.0007	0.2119	0.6179	0.0179	0.0139	0.6915	0.8159	0.0287	0.0287	0.0446	0.0548
Illumina	Weighted bin GC and chi corrected	0.6915	0.5793	0.7881	0.9452	0.2119	0.2743	0.4207	0.6915	0.1357	0.1151	0.1587	0.2119
	Weighted bin GC corrected	0.5000	0.6179	0.6915	0.8159	0.242	0.2119	0.4207	0.5793	0.0228	0.0359	0.0287	0.0446
	LOESS GC and chi corrected	0.6915	0.5793	0.8159	0.9713	0.2119	0.242	0.4207	0.6915	0.1357	0.1357	0.1587	0.2119
	LOESS GC corrected	0.5793	0.5793	0.7881	0.9032	0.1841	0.1357	0.3821	0.5793	0.0228	0.0287	0.0287	0.0446
	Peak and chi corrected	0.0003	0.0002	0.4207	0.8159	0.0139	0.0107	0.3821	0.5793	0.0446	0.0287	0.0668	0.0968
	Peak corrected	0.0001	0.0001	0.0062	0.6179	0.0002	0.0001	0.3446	0.5793	0.0107	0.0082	0.0446	0.0668
	Peak, weighted bin GC and chi corrected	0.6915	0.5793	0.7881	0.9032	0.2119	0.242	0.4207	0.6179	0.1357	0.1357	0.1587	0.242
	Peak and weighted bin GC corrected	0.6179	0.5000	0.8159	0.9032	0.242	0.242	0.4207	0.5793	0.0808	0.1357	0.0808	0.1357
	Peak, LOESS GC and chi corrected	0.6915	0.6179	0.8159	0.9713	0.2119	0.2119	0.3821	0.6179	0.1357	0.1357	0.1587	0.2119
	Peak and LOESS GC corrected	0.6915	0.5793	0.8159	0.9032	0.2119	0.242	0.3821	0.5793	0.0668	0.1357	0.0808	0.1357
	Chi corrected	0.0003	0.0002	0.4207	0.8159	0.0139	0.0107	0.3821	0.5793	0.0446	0.0359	0.0548	0.0968
	Uncorrected	0.0001	0.0000	0.0808	0.5793	0.0002	0.0001	0.3446	0.5000	0.0082	0.0062	0.0228	0.0446

Table S5.4: Theoretical sensitivities for trisomy detection for all combinations of variation reduction and prediction algorithms at a threshold of Z=4 (specificity ~ 100%) in case of a percentage of fetal DNA of 2%.

sensitivity at 3% cffDNA<br>Threshold Z=2		Chr13				Chr18				Chr21			
		Z-score	MAD	NCV	RBZ	Z-score	MAD	NCV	RBZ	Z-score	MAD	NCV	RBZ
SOLiD	Weighted bin GC and chi corrected	1.0000	1.0000	1.0000	1.0000	0.9997	0.9974	1.0000	1.0000	0.9938	0.9965	0.9993	0.9999
	Weighted bin GC corrected	1.0000	1.0000	1.0000	1.0000	0.9893	0.9713	1.0000	1.0000	0.9032	0.9032	0.9452	0.9641
	LOESS GC and chi corrected	1.0000	1.0000	1.0000	1.0000	0.9998	0.9993	1.0000	1.0000	0.9938	0.9938	0.9987	0.9999
	LOESS GC corrected	1.0000	1.0000	1.0000	1.0000	0.9893	0.9861	1.0000	1.0000	0.9032	0.8159	0.9452	0.9641
	Peak and chi corrected	0.4207	0.3821	1.0000	1.0000	0.9893	0.9641	1.0000	1.0000	0.9713	0.9893	0.9938	0.9993
	Peak corrected	0.2119	0.1841	0.9974	1.0000	0.8159	0.7257	1.0000	1.0000	0.9554	0.9893	0.9893	0.9987
	Peak, weighted bin GC and chi corrected	1.0000	1.0000	1.0000	1.0000	0.9997	0.9987	1.0000	1.0000	0.9938	0.9974	0.9993	0.9999
	Peak and weighted bin GC corrected	1.0000	1.0000	1.0000	1.0000	0.9893	0.9893	1.0000	1.0000	0.9893	0.9993	0.9965	0.9987
	Peak, LOESS GC and chi corrected	1.0000	1.0000	1.0000	1.0000	0.9998	0.9997	1.0000	1.0000	0.9938	0.9974	0.9987	0.9999
	Peak and LOESS GC corrected	1.0000	1.0000	1.0000	1.0000	0.9918	0.9861	1.0000	1.0000	0.9893	0.9965	0.9938	0.9987
	Chi corrected	0.4602	0.3821	1.0000	1.0000	0.9861	0.9713	1.0000	1.0000	0.9713	0.9918	0.9965	0.9993
	Uncorrected	0.2119	0.2119	0.9974	1.0000	0.7881	0.7257	1.0000	1.0000	0.8849	0.8849	0.9192	0.9452
Illumina	Weighted bin GC and chi corrected	1.0000	1.0000	1.0000	1.0000	0.9974	0.9993	0.9999	1.0000	0.9893	0.9861	0.9938	0.9974
	Weighted bin GC corrected	1.0000	1.0000	1.0000	1.0000	0.9987	0.9974	0.9999	1.0000	0.8159	0.9032	0.8849	0.9332
	LOESS GC and chi corrected	1.0000	1.0000	1.0000	1.0000	0.9974	0.9987	0.9999	1.0000	0.9893	0.9893	0.9938	0.9974
	LOESS GC corrected	1.0000	1.0000	1.0000	1.0000	0.9965	0.9918	0.9998	1.0000	0.8159	0.8849	0.8849	0.9192
	Peak and chi corrected	0.1151	0.0808	0.9999	1.0000	0.758	0.6915	0.9998	1.0000	0.9192	0.8643	0.9641	0.9821
	Peak corrected	0.0446	0.0359	0.5793	1.0000	0.0808	0.0668	0.9997	1.0000	0.7257	0.6554	0.9332	0.9641
	Peak, weighted bin GC and chi corrected	1.0000	1.0000	1.0000	1.0000	0.9974	0.9987	0.9999	1.0000	0.9918	0.9918	0.9938	0.9987
	Peak and weighted bin GC corrected	1.0000	1.0000	1.0000	1.0000	0.9987	0.9987	0.9999	1.0000	0.9641	0.9893	0.9713	0.9893
	Peak, LOESS GC and chi corrected	1.0000	1.0000	1.0000	1.0000	0.9974	0.9974	0.9998	1.0000	0.9893	0.9893	0.9938	0.9974
	Peak and LOESS GC corrected	1.0000	1.0000	1.0000	1.0000	0.9974	0.9987	0.9998	1.0000	0.9641	0.9918	0.9713	0.9893
	Chi corrected	0.1151	0.0808	0.9999	1.0000	0.758	0.6915	0.9998	1.0000	0.9192	0.9032	0.9554	0.9821
	Uncorrected	0.0446	0.0359	0.9713	1.0000	0.0808	0.0668	0.9997	1.0000	0.6179	0.6179	0.8159	0.9192

Table S5. 5: Theoretical sensitivities for trisomy detection for all combinations of variation reduction and prediction algorithms at a threshold of Z=2 (specificity = 97,72%) in case of a percentage of fetal DNA of 3%.

sensitivity at 3% cffDNA<br><br>Threshold Z=3		Chr13				Chr18				Chr21			
		Z-score	MAD	NCV	RBZ	Z-score	MAD	NCV	RBZ	Z-score	MAD	NCV	RBZ
SOLiD	Weighted bin GC and chi corrected	1.0000	1.0000	1.0000	1.0000	0.9918	0.9641	1.0000	1.0000	0.9332	0.9554	0.9861	0.9974
	Weighted bin GC corrected	0.9995	0.9998	1.0000	1.0000	0.9032	0.8159	0.9998	1.0000	0.6179	0.6179	0.7257	0.7881
	LOESS GC and chi corrected	1.0000	1.0000	1.0000	1.0000	0.9953	0.9861	1.0000	1.0000	0.9332	0.9332	0.9772	0.9974
	LOESS GC corrected	0.9995	0.9998	1.0000	1.0000	0.9032	0.8849	0.9998	1.0000	0.6179	0.4602	0.7257	0.7881
	Peak and chi corrected	0.1151	0.0968	0.9998	1.0000	0.9032	0.7881	1.0000	1.0000	0.8159	0.9032	0.9332	0.9861
	Peak corrected	0.0359	0.0287	0.9641	0.9998	0.4602	0.3446	0.9999	1.0000	0.758	0.9032	0.9032	0.9772
	Peak, weighted bin GC and chi corrected	1.0000	1.0000	1.0000	1.0000	0.9918	0.9772	1.0000	1.0000	0.9332	0.9641	0.9861	0.9974
	Peak and weighted bin GC corrected	0.9998	1.0000	1.0000	1.0000	0.9032	0.9032	0.9999	1.0000	0.9032	0.9861	0.9554	0.9772
	Peak, LOESS GC and chi corrected	1.0000	1.0000	1.0000	1.0000	0.9953	0.9918	1.0000	1.0000	0.9332	0.9641	0.9772	0.9974
	Peak and LOESS GC corrected	0.9998	1.0000	1.0000	1.0000	0.9192	0.8849	0.9999	1.0000	0.9032	0.9554	0.9332	0.9772
	Chi corrected	0.1357	0.0968	0.9998	1.0000	0.8849	0.8159	1.0000	1.0000	0.8159	0.9192	0.9554	0.9861
	Uncorrected	0.0359	0.0359	0.9641	0.9998	0.4207	0.3446	0.9999	1.0000	0.5793	0.5793	0.6554	0.7257
Illumina	Weighted bin GC and chi corrected	0.9999	0.9995	1.0000	1.0000	0.9641	0.9861	0.9974	0.9999	0.9032	0.8849	0.9332	0.9641
	Weighted bin GC corrected	0.9987	0.9998	0.9999	1.0000	0.9772	0.9641	0.9974	0.9995	0.5000	0.6179	0.5793	0.6915
	LOESS GC and chi corrected	0.9999	0.9995	1.0000	1.0000	0.9641	0.9772	0.9974	0.9999	0.9032	0.9032	0.9332	0.9641
	LOESS GC corrected	0.9995	0.9995	1.0000	1.0000	0.9554	0.9192	0.9953	0.9995	0.4602	0.5793	0.5793	0.6554
	Peak and chi corrected	0.0139	0.0082	0.9974	1.0000	0.3821	0.3085	0.9953	0.9995	0.6554	0.5398	0.7881	0.8643
	Peak corrected	0.0035	0.0026	0.2119	0.9998	0.0082	0.0062	0.9918	0.9995	0.3446	0.2743	0.6915	0.7881
	Peak, weighted bin GC and chi corrected	0.9999	0.9995	1.0000	1.0000	0.9641	0.9772	0.9974	0.9998	0.9192	0.9192	0.9332	0.9772
	Peak and weighted bin GC corrected	0.9998	0.9987	1.0000	1.0000	0.9772	0.9772	0.9974	0.9995	0.7881	0.9032	0.8159	0.9032
	Peak, LOESS GC and chi corrected	0.9999	0.9998	1.0000	1.0000	0.9641	0.9641	0.9953	0.9998	0.9032	0.9032	0.9332	0.9641
	Peak and LOESS GC corrected	0.9999	0.9995	1.0000	1.0000	0.9641	0.9772	0.9953	0.9995	0.7881	0.9192	0.8159	0.9032
	Chi corrected	0.0139	0.0082	0.9974	1.0000	0.3821	0.3085	0.9953	0.9995	0.6554	0.6179	0.758	0.8643
	Uncorrected	0.0035	0.0026	0.8159	0.9995	0.0082	0.0062	0.9918	0.9987	0.242	0.242	0.5000	0.6554

Table S5.6: Theoretical sensitivities for trisomy detection for all combinations of variation reduction and prediction algorithms at a threshold of Z=3 (specificity = 99,87%) in case of a percentage of fetal DNA of 3%.

sensitivity at 3% cffDNA<br><br>Threshold Z=4		Chr13				Chr18				Chr21			
		Z-score	MAD	NCV	RBZ	Z-score	MAD	NCV	RBZ	Z-score	MAD	NCV	RBZ
SOLiD	Weighted bin GC and chi corrected	0.9990	1.0000	0.9998	1.0000	0.9192	0.7881	0.9990	1.0000	0.6915	0.758	0.8849	0.9641
	Weighted bin GC corrected	0.9893	0.9938	0.9990	0.9998	0.6179	0.4602	0.9938	0.9998	0.242	0.242	0.3446	0.4207
	LOESS GC and chi corrected	0.9990	1.0000	0.9998	1.0000	0.9452	0.8849	0.9998	1.0000	0.6915	0.6915	0.8159	0.9641
	LOESS GC corrected	0.9893	0.9938	0.9990	0.9998	0.6179	0.5793	0.9938	0.9998	0.242	0.1357	0.3446	0.4207
	Peak and chi corrected	0.0139	0.0107	0.9938	0.9998	0.6179	0.4207	0.9990	1.0000	0.4602	0.6179	0.6915	0.8849
	Peak corrected	0.0026	0.0019	0.7881	0.9938	0.1357	0.0808	0.9974	0.9998	0.3821	0.6179	0.6179	0.8159
	Peak, weighted bin GC and chi corrected	0.9990	1.0000	0.9998	1.0000	0.9192	0.8159	0.9998	1.0000	0.6915	0.7881	0.8849	0.9641
	Peak and weighted bin GC corrected	0.9938	0.9998	0.9990	0.9998	0.6179	0.6179	0.9974	1.0000	0.6179	0.8849	0.758	0.8159
	Peak, LOESS GC and chi corrected	0.9990	1.0000	0.9998	1.0000	0.9452	0.9192	0.9998	1.0000	0.6915	0.7881	0.8159	0.9641
	Peak and LOESS GC corrected	0.9938	0.9998	0.9990	1.0000	0.6554	0.5793	0.9974	0.9998	0.6179	0.758	0.6915	0.8159
	Chi corrected	0.0179	0.0107	0.9938	0.9998	0.5793	0.4602	0.9998	1.0000	0.4602	0.6554	0.758	0.8849
	Uncorrected	0.0026	0.0026	0.7881	0.9938	0.1151	0.0808	0.9974	0.9998	0.2119	0.2119	0.2743	0.3446
Illumina	Weighted bin GC and chi corrected	0.9974	0.9893	0.9990	1.0000	0.7881	0.8849	0.9641	0.9974	0.6179	0.5793	0.6915	0.7881
	Weighted bin GC corrected	0.9772	0.9938	0.9974	0.9998	0.8159	0.7881	0.9641	0.9893	0.1587	0.242	0.2119	0.3085
	LOESS GC and chi corrected	0.9974	0.9893	0.9998	1.0000	0.7881	0.8159	0.9641	0.9974	0.6179	0.6179	0.6915	0.7881
	LOESS GC corrected	0.9893	0.9893	0.9990	1.0000	0.758	0.6554	0.9452	0.9893	0.1357	0.2119	0.2119	0.2743
	Peak and chi corrected	0.0007	0.0003	0.9641	0.9998	0.0968	0.0668	0.9452	0.9893	0.2743	0.1841	0.4207	0.5398
	Peak corrected	0.0001	0.0001	0.0359	0.9938	0.0003	0.0002	0.9192	0.9893	0.0808	0.0548	0.3085	0.4207
	Peak, weighted bin GC and chi corrected	0.9974	0.9893	0.9990	1.0000	0.7881	0.8159	0.9641	0.9938	0.6554	0.6554	0.6915	0.8159
	Peak and weighted bin GC corrected	0.9938	0.9772	0.9998	1.0000	0.8159	0.8159	0.9641	0.9893	0.4207	0.6179	0.4602	0.6179
	Peak, LOESS GC and chi corrected	0.9974	0.9938	0.9998	1.0000	0.7881	0.7881	0.9452	0.9938	0.6179	0.6179	0.6915	0.7881
	Peak and LOESS GC corrected	0.9974	0.9893	0.9998	1.0000	0.7881	0.8159	0.9452	0.9893	0.4207	0.6554	0.4602	0.6179
	Chi corrected	0.0007	0.0003	0.9641	0.9998	0.0968	0.0668	0.9452	0.9893	0.2743	0.242	0.3821	0.5398
	Uncorrected	0.0001	0.0001	0.4602	0.9893	0.0003	0.0002	0.9192	0.9772	0.0446	0.0446	0.1587	0.2743

Table S5. 7: Theoretical sensitivities for trisomy detection for all combinations of variation reduction and prediction algorithms at a threshold of Z=4 (specificity ~ 100%) in case of a percentage of fetal DNA of 3%.

sensitivity at 4% cffDNA<br>Threshold Z=2		Chr13				Chr18				Chr21			
		Z-score	MAD	NCV	RBZ	Z-score	MAD	NCV	RBZ	Z-score	MAD	NCV	RBZ
SOLiD	Weighted bin GC and chi corrected	1.0000	1.0000	1.0000	1.0000	1.0000	1.0000	1.0000	1.0000	1.0000	1.0000	1.0000	1.0000
	Weighted bin GC corrected	1.0000	1.0000	1.0000	1.0000	0.9999	0.9995	1.0000	1.0000	0.9918	0.9893	0.9974	0.9987
	LOESS GC and chi corrected	1.0000	1.0000	1.0000	1.0000	1.0000	1.0000	1.0000	1.0000	1.0000	1.0000	1.0000	1.0000
	LOESS GC corrected	1.0000	1.0000	1.0000	1.0000	0.9999	0.9998	1.0000	1.0000	0.9893	0.9713	0.9974	0.9987
	Peak and chi corrected	0.6554	0.6179	1.0000	1.0000	0.9999	0.9990	1.0000	1.0000	0.9995	0.9999	1.0000	1.0000
	Peak corrected	0.3446	0.3085	1.0000	1.0000	0.9641	0.9332	1.0000	1.0000	0.9981	0.9999	0.9999	1.0000
	Peak, weighted bin GC and chi corrected	1.0000	1.0000	1.0000	1.0000	1.0000	1.0000	1.0000	1.0000	1.0000	1.0000	1.0000	1.0000
	Peak and weighted bin GC corrected	1.0000	1.0000	1.0000	1.0000	0.9999	0.9999	1.0000	1.0000	0.9999	1.0000	1.0000	1.0000
	Peak, LOESS GC and chi corrected	1.0000	1.0000	1.0000	1.0000	1.0000	1.0000	1.0000	1.0000	1.0000	1.0000	1.0000	1.0000
	Peak and LOESS GC corrected	1.0000	1.0000	1.0000	1.0000	1.0000	0.9998	1.0000	1.0000	0.9999	1.0000	1.0000	1.0000
	Chi corrected	0.6915	0.6179	1.0000	1.0000	0.9998	0.9995	1.0000	1.0000	0.9995	1.0000	1.0000	1.0000
	Uncorrected	0.3446	0.3085	1.0000	1.0000	0.9641	0.9332	1.0000	1.0000	0.9893	0.9893	0.9938	0.9974
Illumina	Weighted bin GC and chi corrected	1.0000	1.0000	1.0000	1.0000	1.0000	1.0000	1.0000	1.0000	0.9999	0.9998	1.0000	1.0000
	Weighted bin GC corrected	1.0000	1.0000	1.0000	1.0000	1.0000	1.0000	1.0000	1.0000	0.9772	0.9918	0.9893	0.9965
	LOESS GC and chi corrected	1.0000	1.0000	1.0000	1.0000	1.0000	1.0000	1.0000	1.0000	0.9999	0.9999	1.0000	1.0000
	LOESS GC corrected	1.0000	1.0000	1.0000	1.0000	1.0000	1.0000	1.0000	1.0000	0.9713	0.9893	0.9893	0.9938
	Peak and chi corrected	0.1841	0.1357	1.0000	1.0000	0.9452	0.9032	1.0000	1.0000	0.9938	0.9861	0.9987	0.9997
	Peak corrected	0.0548	0.0446	0.8159	1.0000	0.1357	0.0808	1.0000	1.0000	0.9192	0.8849	0.9965	0.9987
	Peak, weighted bin GC and chi corrected	1.0000	1.0000	1.0000	1.0000	1.0000	1.0000	1.0000	1.0000	1.0000	1.0000	1.0000	1.0000
	Peak and weighted bin GC corrected	1.0000	1.0000	1.0000	1.0000	1.0000	1.0000	1.0000	1.0000	0.9990	0.9999	0.9995	0.9999
	Peak, LOESS GC and chi corrected	1.0000	1.0000	1.0000	1.0000	1.0000	1.0000	1.0000	1.0000	0.9999	0.9999	1.0000	1.0000
	Peak and LOESS GC corrected	1.0000	1.0000	1.0000	1.0000	1.0000	1.0000	1.0000	1.0000	0.9987	1.0000	0.9995	0.9999
	Chi corrected	0.1841	0.1151	1.0000	1.0000	0.9452	0.9192	1.0000	1.0000	0.9938	0.9893	0.9981	0.9997
	Uncorrected	0.0548	0.0446	0.9995	1.0000	0.1357	0.0808	1.0000	1.0000	0.8643	0.8643	0.9772	0.9938

Table S5.8: Theoretical sensitivities for trisomy detection for all combinations of variation reduction and prediction algorithms at a threshold of Z=2 (specificity = 97,72%) in case of a percentage of fetal DNA of 4%.

sensitivity at 4% cffDNA<br><br>Threshold Z=3		Chr13				Chr18				Chr21			
		Z-score	MAD	NCV	RBZ	Z-score	MAD	NCV	RBZ	Z-score	MAD	NCV	RBZ
SOLiD	Weighted bin GC and chi corrected	1.0000	1.0000	1.0000	1.0000	1.0000	0.9998	1.0000	1.0000	0.9990	0.9995	1.0000	1.0000
	Weighted bin GC corrected	1.0000	1.0000	1.0000	1.0000	0.9965	0.9893	1.0000	1.0000	0.9192	0.9032	0.9641	0.9772
	LOESS GC and chi corrected	1.0000	1.0000	1.0000	1.0000	1.0000	1.0000	1.0000	1.0000	0.9990	0.9990	0.9999	1.0000
	LOESS GC corrected	1.0000	1.0000	1.0000	1.0000	0.9965	0.9953	1.0000	1.0000	0.9032	0.8159	0.9641	0.9772
	Peak and chi corrected	0.2743	0.242	1.0000	1.0000	0.9965	0.9821	1.0000	1.0000	0.9893	0.9965	0.9990	1.0000
	Peak corrected	0.0808	0.0668	0.9998	1.0000	0.7881	0.6915	1.0000	1.0000	0.9713	0.9965	0.9965	0.9999
	Peak, weighted bin GC and chi corrected	1.0000	1.0000	1.0000	1.0000	1.0000	0.9999	1.0000	1.0000	0.9990	0.9998	1.0000	1.0000
	Peak and weighted bin GC corrected	1.0000	1.0000	1.0000	1.0000	0.9965	0.9965	1.0000	1.0000	0.9965	1.0000	0.9995	0.9999
	Peak, LOESS GC and chi corrected	1.0000	1.0000	1.0000	1.0000	1.0000	1.0000	1.0000	1.0000	0.9990	0.9998	0.9999	1.0000
	Peak and LOESS GC corrected	1.0000	1.0000	1.0000	1.0000	0.9981	0.9953	1.0000	1.0000	0.9965	0.9995	0.9990	0.9999
	Chi corrected	0.3085	0.242	1.0000	1.0000	0.9953	0.9893	1.0000	1.0000	0.9893	0.9981	0.9995	1.0000
	Uncorrected	0.0808	0.0668	0.9998	1.0000	0.7881	0.6915	1.0000	1.0000	0.9032	0.9032	0.9332	0.9641
Illumina	Weighted bin GC and chi corrected	1.0000	1.0000	1.0000	1.0000	0.9998	1.0000	1.0000	1.0000	0.9965	0.9953	0.9990	0.9998
	Weighted bin GC corrected	1.0000	1.0000	1.0000	1.0000	0.9999	0.9998	1.0000	1.0000	0.8159	0.9192	0.9032	0.9554
	LOESS GC and chi corrected	1.0000	1.0000	1.0000	1.0000	0.9998	0.9999	1.0000	1.0000	0.9965	0.9965	0.9990	0.9998
	LOESS GC corrected	1.0000	1.0000	1.0000	1.0000	0.9995	0.9981	1.0000	1.0000	0.8159	0.9032	0.9032	0.9332
	Peak and chi corrected	0.0287	0.0179	1.0000	1.0000	0.7257	0.6179	1.0000	1.0000	0.9332	0.8849	0.9772	0.9918
	Peak corrected	0.0047	0.0035	0.5000	1.0000	0.0179	0.0082	1.0000	1.0000	0.6554	0.5793	0.9554	0.9772
	Peak, weighted bin GC and chi corrected	1.0000	1.0000	1.0000	1.0000	0.9998	0.9999	1.0000	1.0000	0.9981	0.9981	0.9990	0.9999
	Peak and weighted bin GC corrected	1.0000	1.0000	1.0000	1.0000	0.9999	0.9999	1.0000	1.0000	0.9821	0.9965	0.9893	0.9965
	Peak, LOESS GC and chi corrected	1.0000	1.0000	1.0000	1.0000	0.9998	0.9998	1.0000	1.0000	0.9965	0.9965	0.9990	0.9998
	Peak and LOESS GC corrected	1.0000	1.0000	1.0000	1.0000	0.9998	0.9999	1.0000	1.0000	0.9772	0.9981	0.9893	0.9965
	Chi corrected	0.0287	0.0139	1.0000	1.0000	0.7257	0.6554	1.0000	1.0000	0.9332	0.9032	0.9713	0.9918
	Uncorrected	0.0047	0.0035	0.9893	1.0000	0.0179	0.0082	1.0000	1.0000	0.5398	0.5398	0.8159	0.9332

Table S5. 9: Theoretical sensitivities for trisomy detection for all combinations of variation reduction and prediction algorithms at a threshold of Z=3 (specificity = 99,87%) in case of a percentage of fetal DNA of 4%.

sensitivity at 4% cffDNA<br>Threshold Z=4		Chr13				Chr18				Chr21			
		Z-score	MAD	NCV	RBZ	Z-score	MAD	NCV	RBZ	Z-score	MAD	NCV	RBZ
SOLiD	Weighted bin GC and chi corrected	1.0000	1.0000	1.0000	1.0000	0.9990	0.9938	1.0000	1.0000	0.9821	0.9893	0.9981	0.9999
	Weighted bin GC corrected	1.0000	1.0000	1.0000	1.0000	0.9554	0.9032	1.0000	1.0000	0.6554	0.6179	0.7881	0.8159
	LOESS GC and chi corrected	1.0000	1.0000	1.0000	1.0000	0.9997	0.9981	1.0000	1.0000	0.9821	0.9821	0.9965	0.9999
	LOESS GC corrected	1.0000	1.0000	1.0000	1.0000	0.9554	0.9452	1.0000	1.0000	0.6179	0.4602	0.7881	0.8159
	Peak and chi corrected	0.0548	0.0446	1.0000	1.0000	0.9554	0.8643	1.0000	1.0000	0.9032	0.9554	0.9821	0.9981
	Peak corrected	0.0082	0.0062	0.9938	1.0000	0.4207	0.3085	1.0000	1.0000	0.8159	0.9554	0.9554	0.9965
	Peak, weighted bin GC and chi corrected	1.0000	1.0000	1.0000	1.0000	0.9990	0.9965	1.0000	1.0000	0.9821	0.9938	0.9981	0.9999
	Peak and weighted bin GC corrected	1.0000	1.0000	1.0000	1.0000	0.9554	0.9554	1.0000	1.0000	0.9554	0.9981	0.9893	0.9965
	Peak, LOESS GC and chi corrected	1.0000	1.0000	1.0000	1.0000	0.9997	0.9990	1.0000	1.0000	0.9821	0.9938	0.9965	0.9999
	Peak and LOESS GC corrected	1.0000	1.0000	1.0000	1.0000	0.9713	0.9452	1.0000	1.0000	0.9554	0.9893	0.9821	0.9965
	Chi corrected	0.0668	0.0446	1.0000	1.0000	0.9452	0.9032	1.0000	1.0000	0.9032	0.9713	0.9893	0.9981
	Uncorrected	0.0082	0.0062	0.9938	1.0000	0.4207	0.3085	1.0000	1.0000	0.6179	0.6179	0.6915	0.7881
Illumina	Weighted bin GC and chi corrected	1.0000	1.0000	1.0000	1.0000	0.9938	0.9981	0.9999	1.0000	0.9554	0.9452	0.9821	0.9938
	Weighted bin GC corrected	1.0000	1.0000	1.0000	1.0000	0.9965	0.9938	0.9999	1.0000	0.5000	0.6554	0.6179	0.758
	LOESS GC and chi corrected	1.0000	1.0000	1.0000	1.0000	0.9938	0.9965	0.9999	1.0000	0.9554	0.9554	0.9821	0.9938
	LOESS GC corrected	1.0000	1.0000	1.0000	1.0000	0.9893	0.9713	0.9997	1.0000	0.4602	0.6179	0.6179	0.6915
	Peak and chi corrected	0.0019	0.0010	0.9999	1.0000	0.3446	0.242	0.9997	1.0000	0.6915	0.5793	0.8159	0.9192
	Peak corrected	0.0002	0.0001	0.1587	1.0000	0.0010	0.0003	0.9990	1.0000	0.2743	0.2119	0.758	0.8159
	Peak, weighted bin GC and chi corrected	1.0000	1.0000	1.0000	1.0000	0.9938	0.9965	0.9999	1.0000	0.9713	0.9713	0.9821	0.9965
	Peak and weighted bin GC corrected	1.0000	1.0000	1.0000	1.0000	0.9965	0.9965	0.9999	1.0000	0.8643	0.9554	0.9032	0.9554
	Peak, LOESS GC and chi corrected	1.0000	1.0000	1.0000	1.0000	0.9938	0.9938	0.9997	1.0000	0.9554	0.9554	0.9821	0.9938
	Peak and LOESS GC corrected	1.0000	1.0000	1.0000	1.0000	0.9938	0.9965	0.9997	1.0000	0.8159	0.9713	0.9032	0.9554
	Chi corrected	0.0019	0.0007	0.9999	1.0000	0.3446	0.2743	0.9997	1.0000	0.6915	0.6179	0.8159	0.9192
	Uncorrected	0.0002	0.0001	0.9032	1.0000	0.0010	0.0003	0.9990	1.0000	0.1841	0.1841	0.5000	0.6915

Table S5.10: Theoretical sensitivities for trisomy detection for all combinations of variation reduction and prediction algorithms at a threshold of Z=4 (specificity ~ 100%) in case of a percentage of fetal DNA of 4%.

sensitivity at 5% cffDNA<br><br>Threshold Z=2		Chr13				Chr18				Chr21			
		Z-score	MAD	NCV	RBZ	Z-score	MAD	NCV	RBZ	Z-score	MAD	NCV	RBZ
SOLiD	Weighted bin GC and chi corrected	1.0000	1.0000	1.0000	1.0000	1.0000	1.0000	1.0000	1.0000	1.0000	1.0000	1.0000	1.0000
	Weighted bin GC corrected	1.0000	1.0000	1.0000	1.0000	1.0000	1.0000	1.0000	1.0000	0.9998	0.9997	1.0000	1.0000
	LOESS GC and chi corrected	1.0000	1.0000	1.0000	1.0000	1.0000	1.0000	1.0000	1.0000	1.0000	1.0000	1.0000	1.0000
	LOESS GC corrected	1.0000	1.0000	1.0000	1.0000	1.0000	1.0000	1.0000	1.0000	0.9997	0.9981	1.0000	1.0000
	Peak and chi corrected	0.8413	0.8159	1.0000	1.0000	1.0000	1.0000	1.0000	1.0000	1.0000	1.0000	1.0000	1.0000
	Peak corrected	0.5398	0.4602	1.0000	1.0000	0.9974	0.9918	1.0000	1.0000	1.0000	1.0000	1.0000	1.0000
	Peak, weighted bin GC and chi corrected	1.0000	1.0000	1.0000	1.0000	1.0000	1.0000	1.0000	1.0000	1.0000	1.0000	1.0000	1.0000
	Peak and weighted bin GC corrected	1.0000	1.0000	1.0000	1.0000	1.0000	1.0000	1.0000	1.0000	1.0000	1.0000	1.0000	1.0000
	Peak, LOESS GC and chi corrected	1.0000	1.0000	1.0000	1.0000	1.0000	1.0000	1.0000	1.0000	1.0000	1.0000	1.0000	1.0000
	Peak and LOESS GC corrected	1.0000	1.0000	1.0000	1.0000	1.0000	1.0000	1.0000	1.0000	1.0000	1.0000	1.0000	1.0000
	Chi corrected	0.8643	0.8159	1.0000	1.0000	1.0000	1.0000	1.0000	1.0000	1.0000	1.0000	1.0000	1.0000
	Uncorrected	0.5398	0.4602	1.0000	1.0000	0.9965	0.9918	1.0000	1.0000	0.9995	0.9995	0.9999	1.0000
Illumina	Weighted bin GC and chi corrected	1.0000	1.0000	1.0000	1.0000	1.0000	1.0000	1.0000	1.0000	1.0000	1.0000	1.0000	1.0000
	Weighted bin GC corrected	1.0000	1.0000	1.0000	1.0000	1.0000	1.0000	1.0000	1.0000	0.9987	0.9998	0.9995	0.9999
	LOESS GC and chi corrected	1.0000	1.0000	1.0000	1.0000	1.0000	1.0000	1.0000	1.0000	1.0000	1.0000	1.0000	1.0000
	LOESS GC corrected	1.0000	1.0000	1.0000	1.0000	1.0000	1.0000	1.0000	1.0000	0.9981	0.9995	0.9995	0.9999
	Peak and chi corrected	0.2743	0.1841	1.0000	1.0000	0.9938	0.9861	1.0000	1.0000	0.9999	0.9993	1.0000	1.0000
	Peak corrected	0.0668	0.0548	0.9554	1.0000	0.1841	0.1151	1.0000	1.0000	0.9893	0.9772	0.9999	1.0000
	Peak, weighted bin GC and chi corrected	1.0000	1.0000	1.0000	1.0000	1.0000	1.0000	1.0000	1.0000	1.0000	1.0000	1.0000	1.0000
	Peak and weighted bin GC corrected	1.0000	1.0000	1.0000	1.0000	1.0000	1.0000	1.0000	1.0000	1.0000	1.0000	1.0000	1.0000
	Peak, LOESS GC and chi corrected	1.0000	1.0000	1.0000	1.0000	1.0000	1.0000	1.0000	1.0000	1.0000	1.0000	1.0000	1.0000
	Peak and LOESS GC corrected	1.0000	1.0000	1.0000	1.0000	1.0000	1.0000	1.0000	1.0000	1.0000	1.0000	1.0000	1.0000
	Chi corrected	0.2743	0.1841	1.0000	1.0000	0.9938	0.9861	1.0000	1.0000	0.9999	0.9997	1.0000	1.0000
	Uncorrected	0.0668	0.0548	1.0000	1.0000	0.1841	0.1151	1.0000	1.0000	0.9713	0.9641	0.9987	0.9999

Table S5.11: Theoretical sensitivities for trisomy detection for all combinations of variation reduction and prediction algorithms at a threshold of Z=2 (specificity = 97,72%) in case of a percentage of fetal DNA of 5%.

sensitivity at 5% cffDNA<br>Threshold Z=3		Chr13				Chr18				Chr21			
		Z-score	MAD	NCV	RBZ	Z-score	MAD	NCV	RBZ	Z-score	MAD	NCV	RBZ
SOLiD	Weighted bin GC and chi corrected	1.0000	1.0000	1.0000	1.0000	1.0000	1.0000	1.0000	1.0000	1.0000	1.0000	1.0000	1.0000
	Weighted bin GC corrected	1.0000	1.0000	1.0000	1.0000	1.0000	0.9998	1.0000	1.0000	0.9953	0.9918	0.9987	0.9995
	LOESS GC and chi corrected	1.0000	1.0000	1.0000	1.0000	1.0000	1.0000	1.0000	1.0000	1.0000	1.0000	1.0000	1.0000
	LOESS GC corrected	1.0000	1.0000	1.0000	1.0000	1.0000	1.0000	1.0000	1.0000	0.9918	0.9713	0.9987	0.9995
	Peak and chi corrected	0.5000	0.4602	1.0000	1.0000	1.0000	0.9997	1.0000	1.0000	0.9998	1.0000	1.0000	1.0000
	Peak corrected	0.1587	0.1357	1.0000	1.0000	0.9641	0.9192	1.0000	1.0000	0.9990	1.0000	1.0000	1.0000
	Peak, weighted bin GC and chi corrected	1.0000	1.0000	1.0000	1.0000	1.0000	1.0000	1.0000	1.0000	1.0000	1.0000	1.0000	1.0000
	Peak and weighted bin GC corrected	1.0000	1.0000	1.0000	1.0000	1.0000	1.0000	1.0000	1.0000	1.0000	1.0000	1.0000	1.0000
	Peak, LOESS GC and chi corrected	1.0000	1.0000	1.0000	1.0000	1.0000	1.0000	1.0000	1.0000	1.0000	1.0000	1.0000	1.0000
	Peak and LOESS GC corrected	1.0000	1.0000	1.0000	1.0000	1.0000	1.0000	1.0000	1.0000	1.0000	1.0000	1.0000	1.0000
	Chi corrected	0.5398	0.4602	1.0000	1.0000	1.0000	0.9998	1.0000	1.0000	0.9998	1.0000	1.0000	1.0000
	Uncorrected	0.1587	0.1357	1.0000	1.0000	0.9554	0.9192	1.0000	1.0000	0.9893	0.9893	0.9965	0.9987
Illumina	Weighted bin GC and chi corrected	1.0000	1.0000	1.0000	1.0000	1.0000	1.0000	1.0000	1.0000	1.0000	1.0000	1.0000	1.0000
	Weighted bin GC corrected	1.0000	1.0000	1.0000	1.0000	1.0000	1.0000	1.0000	1.0000	0.9772	0.9953	0.9893	0.9974
	LOESS GC and chi corrected	1.0000	1.0000	1.0000	1.0000	1.0000	1.0000	1.0000	1.0000	1.0000	1.0000	1.0000	1.0000
	LOESS GC corrected	1.0000	1.0000	1.0000	1.0000	1.0000	1.0000	1.0000	1.0000	0.9713	0.9893	0.9893	0.9965
	Peak and chi corrected	0.0548	0.0287	1.0000	1.0000	0.9332	0.8849	1.0000	1.0000	0.9965	0.9861	0.9995	0.9999
	Peak corrected	0.0062	0.0047	0.758	1.0000	0.0287	0.0139	1.0000	1.0000	0.9032	0.8159	0.9974	0.9995
	Peak, weighted bin GC and chi corrected	1.0000	1.0000	1.0000	1.0000	1.0000	1.0000	1.0000	1.0000	1.0000	1.0000	1.0000	1.0000
	Peak and weighted bin GC corrected	1.0000	1.0000	1.0000	1.0000	1.0000	1.0000	1.0000	1.0000	0.9997	1.0000	0.9998	1.0000
	Peak, LOESS GC and chi corrected	1.0000	1.0000	1.0000	1.0000	1.0000	1.0000	1.0000	1.0000	1.0000	1.0000	1.0000	1.0000
	Peak and LOESS GC corrected	1.0000	1.0000	1.0000	1.0000	1.0000	1.0000	1.0000	1.0000	0.9995	1.0000	0.9998	1.0000
	Chi corrected	0.0548	0.0287	1.0000	1.0000	0.9332	0.8849	1.0000	1.0000	0.9965	0.9918	0.9990	0.9999
	Uncorrected	0.0062	0.0047	0.9998	1.0000	0.0287	0.0139	1.0000	1.0000	0.8159	0.7881	0.9772	0.9965

Table S5.12: Theoretical sensitivities for trisomy detection for all combinations of variation reduction and prediction algorithms at a threshold of Z=3 (specificity = 99,87%) in case of a percentage of fetal DNA of 5%.

sensitivity at 5% cffDNA<br>Threshold Z=4		Chr13				Chr18				Chr21			
		Z-score	MAD	NCV	RBZ	Z-score	MAD	NCV	RBZ	Z-score	MAD	NCV	RBZ
SOLiD	Weighted bin GC and chi corrected	1.0000	1.0000	1.0000	1.0000	1.0000	1.0000	1.0000	1.0000	0.9998	0.9999	1.0000	1.0000
	Weighted bin GC corrected	1.0000	1.0000	1.0000	1.0000	0.9990	0.9953	1.0000	1.0000	0.9452	0.9192	0.9772	0.9893
	LOESS GC and chi corrected	1.0000	1.0000	1.0000	1.0000	1.0000	1.0000	1.0000	1.0000	0.9998	0.9998	1.0000	1.0000
	LOESS GC corrected	1.0000	1.0000	1.0000	1.0000	0.9990	0.9981	1.0000	1.0000	0.9192	0.8159	0.9772	0.9893
	Peak and chi corrected	0.1587	0.1357	1.0000	1.0000	0.9990	0.9918	1.0000	1.0000	0.9953	0.9990	0.9998	1.0000
	Peak corrected	0.0228	0.0179	1.0000	1.0000	0.7881	0.6554	1.0000	1.0000	0.9821	0.9990	0.9990	1.0000
	Peak, weighted bin GC and chi corrected	1.0000	1.0000	1.0000	1.0000	1.0000	1.0000	1.0000	1.0000	0.9998	1.0000	1.0000	1.0000
	Peak and weighted bin GC corrected	1.0000	1.0000	1.0000	1.0000	0.9990	0.9990	1.0000	1.0000	0.9990	1.0000	0.9999	1.0000
	Peak, LOESS GC and chi corrected	1.0000	1.0000	1.0000	1.0000	1.0000	1.0000	1.0000	1.0000	0.9998	1.0000	1.0000	1.0000
	Peak and LOESS GC corrected	1.0000	1.0000	1.0000	1.0000	0.9997	0.9981	1.0000	1.0000	0.9990	0.9999	0.9998	1.0000
	Chi corrected	0.1841	0.1357	1.0000	1.0000	0.9981	0.9953	1.0000	1.0000	0.9953	0.9997	0.9999	1.0000
	Uncorrected	0.0228	0.0179	1.0000	1.0000	0.758	0.6554	1.0000	1.0000	0.9032	0.9032	0.9554	0.9772
Illumina	Weighted bin GC and chi corrected	1.0000	1.0000	1.0000	1.0000	1.0000	1.0000	1.0000	1.0000	0.9990	0.9981	0.9998	1.0000
	Weighted bin GC corrected	1.0000	1.0000	1.0000	1.0000	1.0000	1.0000	1.0000	1.0000	0.8159	0.9452	0.9032	0.9641
	LOESS GC and chi corrected	1.0000	1.0000	1.0000	1.0000	1.0000	1.0000	1.0000	1.0000	0.9990	0.9990	0.9998	1.0000
	LOESS GC corrected	1.0000	1.0000	1.0000	1.0000	0.9999	0.9997	1.0000	1.0000	0.8159	0.9032	0.9032	0.9554
	Peak and chi corrected	0.0047	0.0019	1.0000	1.0000	0.6915	0.5793	1.0000	1.0000	0.9554	0.8849	0.9893	0.9974
	Peak corrected	0.0002	0.0002	0.3821	1.0000	0.0019	0.0007	1.0000	1.0000	0.6179	0.5000	0.9641	0.9893
	Peak, weighted bin GC and chi corrected	1.0000	1.0000	1.0000	1.0000	1.0000	1.0000	1.0000	1.0000	0.9997	0.9997	0.9998	1.0000
	Peak and weighted bin GC corrected	1.0000	1.0000	1.0000	1.0000	1.0000	1.0000	1.0000	1.0000	0.9918	0.9990	0.9953	0.9990
	Peak, LOESS GC and chi corrected	1.0000	1.0000	1.0000	1.0000	1.0000	1.0000	1.0000	1.0000	0.9990	0.9990	0.9998	1.0000
	Peak and LOESS GC corrected	1.0000	1.0000	1.0000	1.0000	1.0000	1.0000	1.0000	1.0000	0.9893	0.9997	0.9953	0.9990
	Chi corrected	0.0047	0.0019	1.0000	1.0000	0.6915	0.5793	1.0000	1.0000	0.9554	0.9192	0.9821	0.9974
	Uncorrected	0.0002	0.0002	0.9953	1.0000	0.0019	0.0007	1.0000	1.0000	0.4602	0.4207	0.8159	0.9554

Table S5. 13: Theoretical sensitivities for trisomy detection for all combinations of variation reduction and prediction algorithms at a threshold of Z=4 (specificity ~100%) in case of a percentage of fetal DNA of 5%.

Based on tables **S5.2 to S5.13** for the uncorrected, the χ^2VR corrected, the LOESS GC corrected and the both LOESS GC and χ^2VR corrected data an ROC curve was created for thresholds Z=2, Z=3 and Z=3 for 2%, 3% and 4% cffDNA for chromosomes 13, 18 and 21 for both SOLiD and Illumina. These curves are shown in **Figure S5.1**.

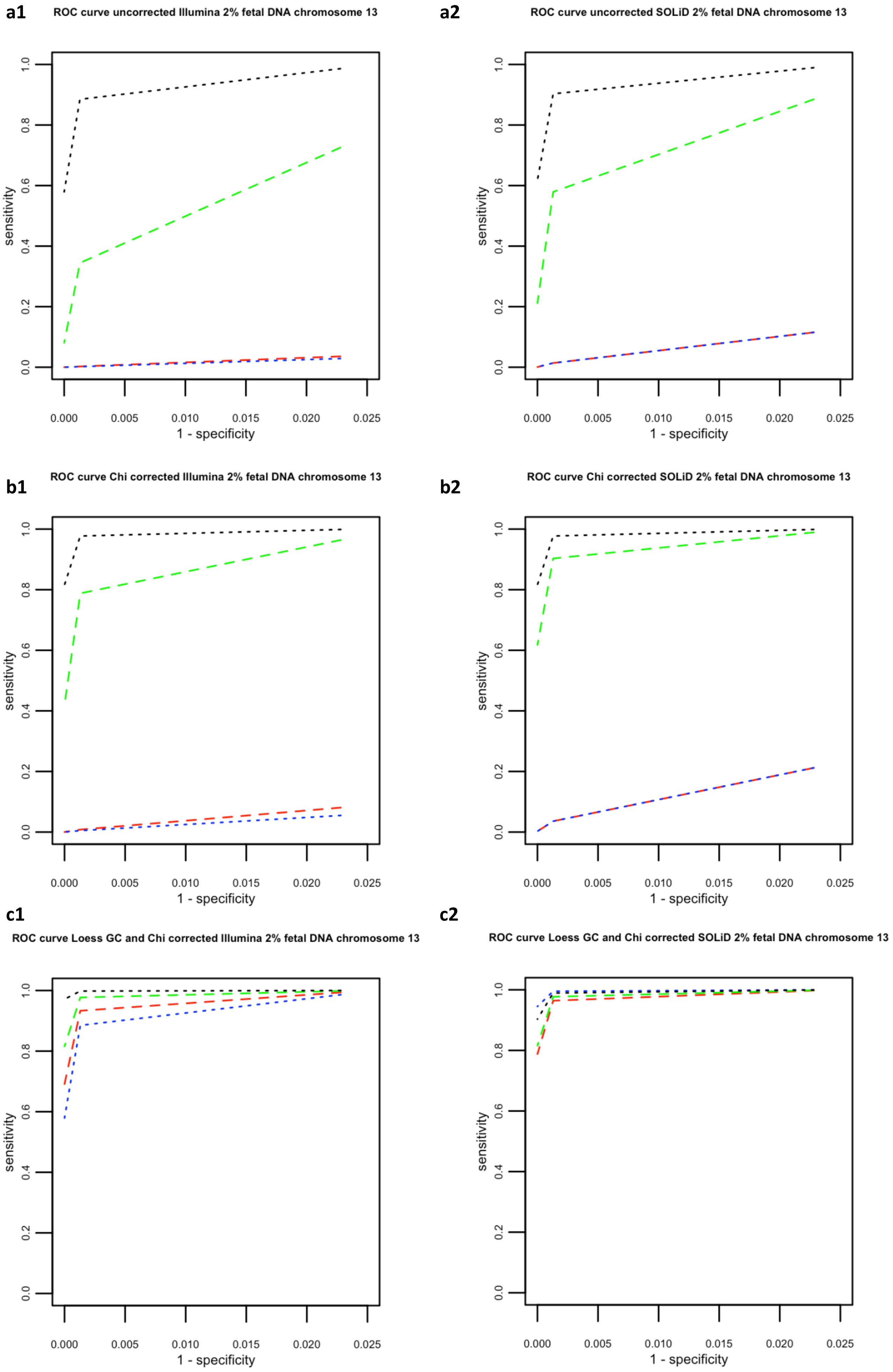
a1
ROC curve uncorrected Illumina 2% fetal DNA chromosome 13
a2
ROC curve uncorrected SOLiD 2% fetal DNA chromosome 13
b1
ROC curve Chi corrected Illumina 2% fetal DNA chromosome 13
b2
ROC curve Chi corrected SOLiD 2% fetal DNA chromosome 13
c1
ROC curve Loess GC and Chi corrected Illumina 2% fetal DNA chromosome 13
c2
ROC curve Loess GC and Chi corrected SOLiD 2% fetal DNA chromosome 13
sensitivity
0.0
0.2
0.4
0.6
0.8
1.0
0.000
0.005
0.010
0.015
0.020
0.025
1 - specificity

d1

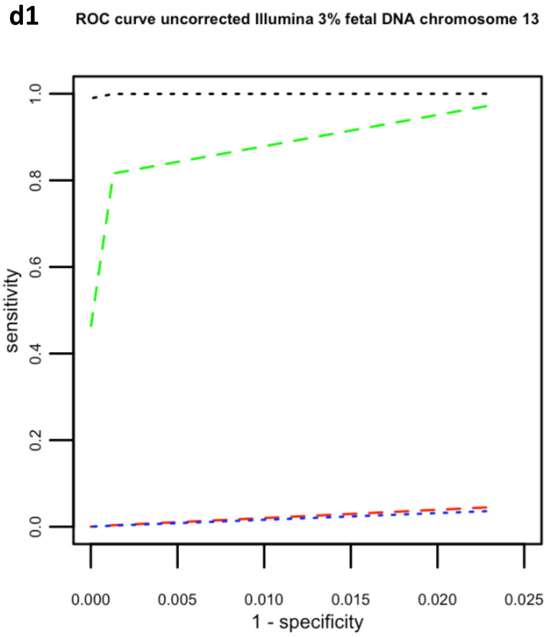

d2

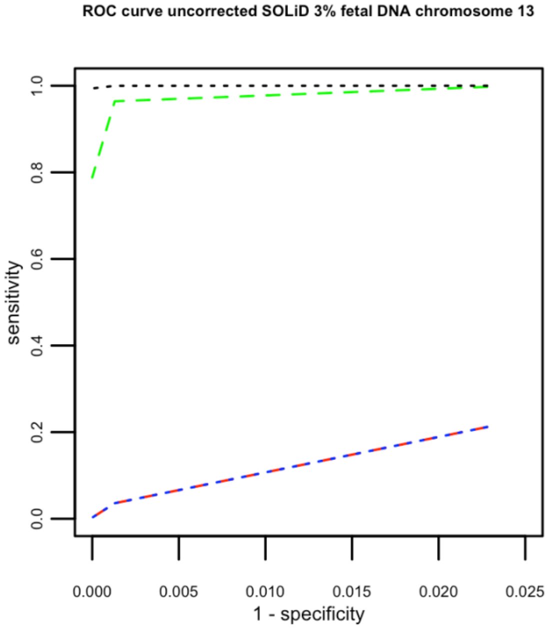

e1

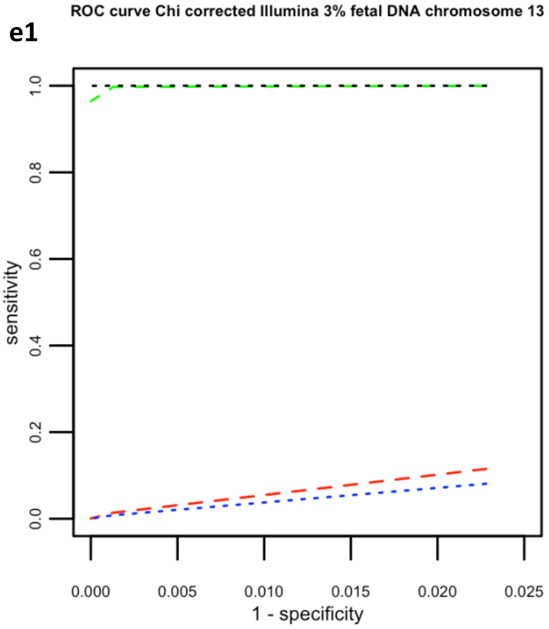

e2

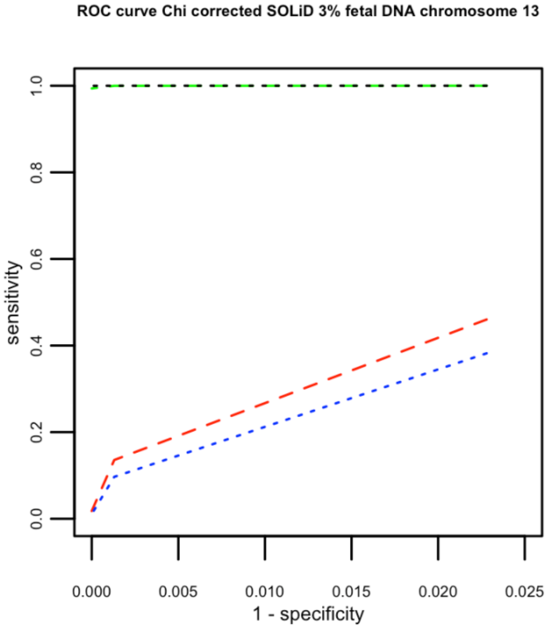

f1

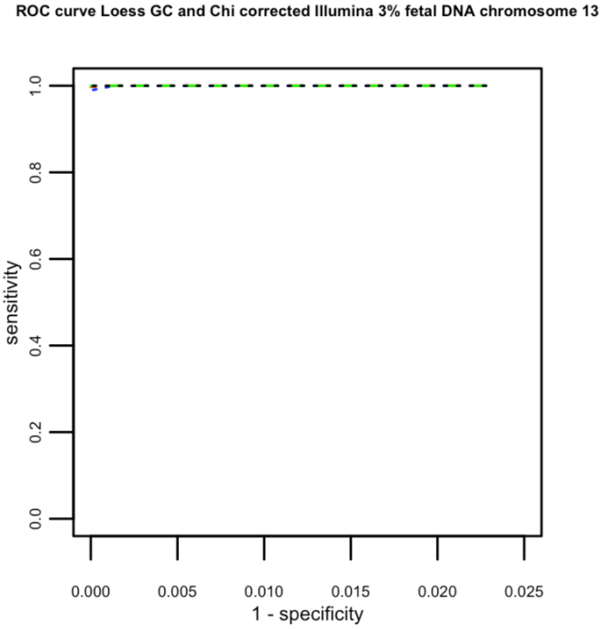

f2

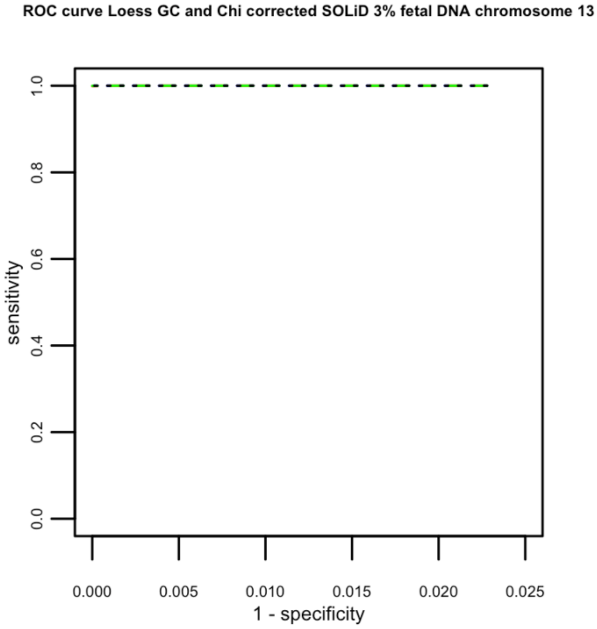

g1

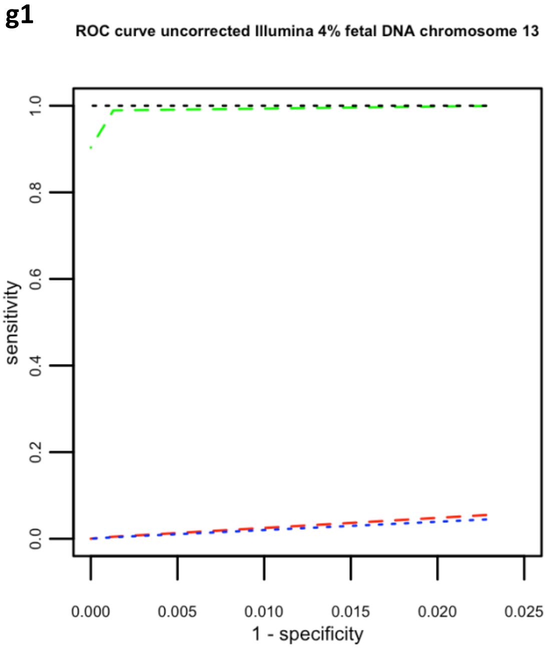

g2

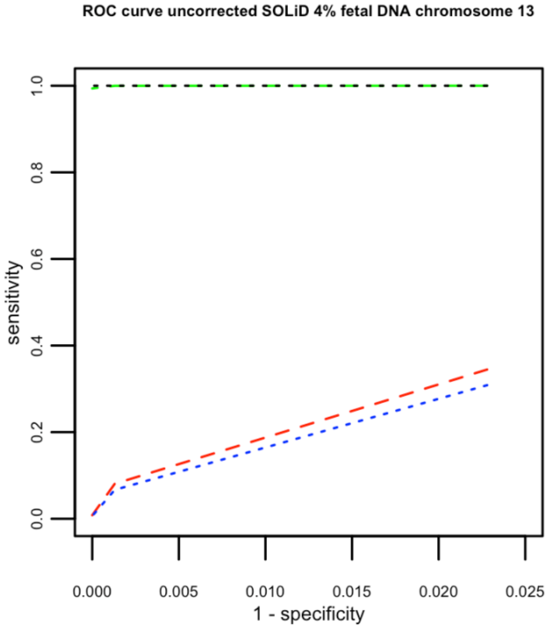

h1

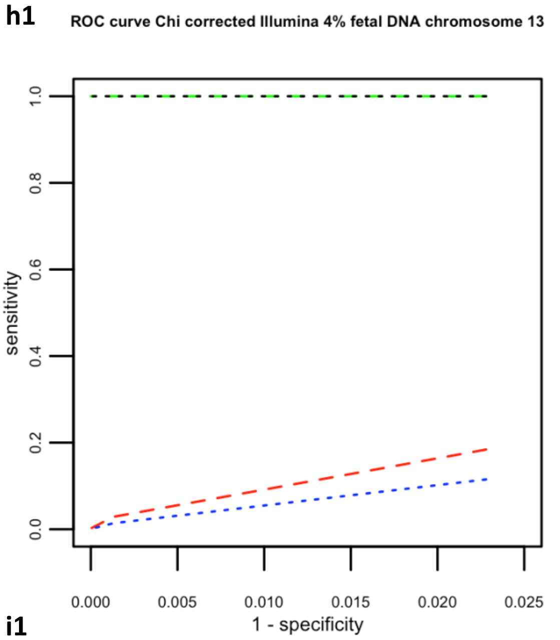

h2

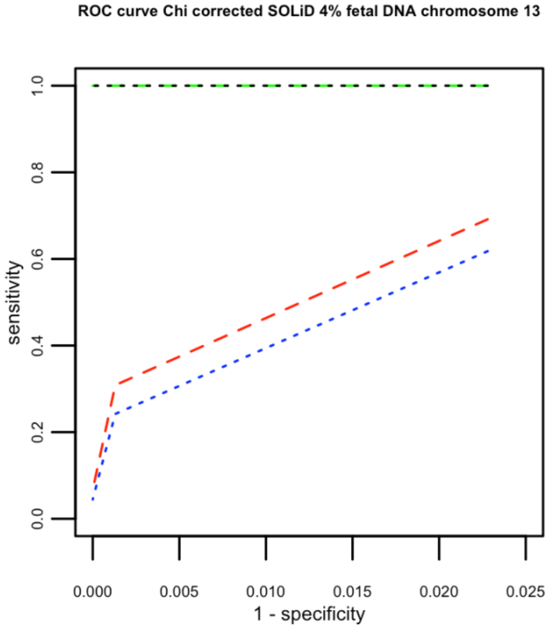

i1

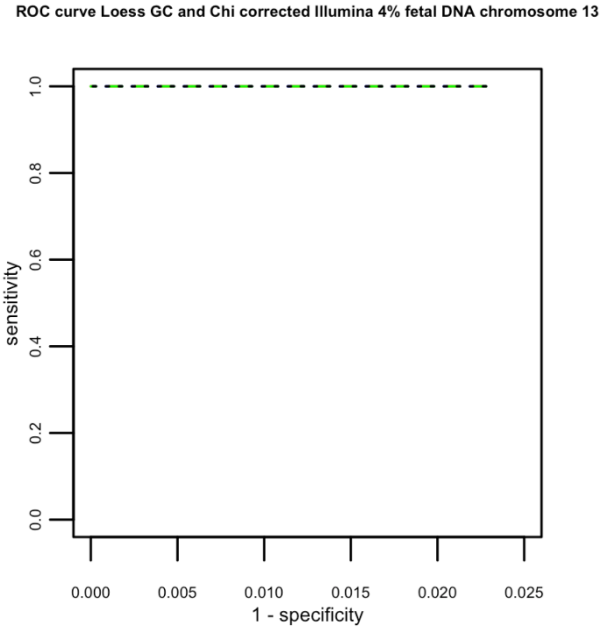

i2

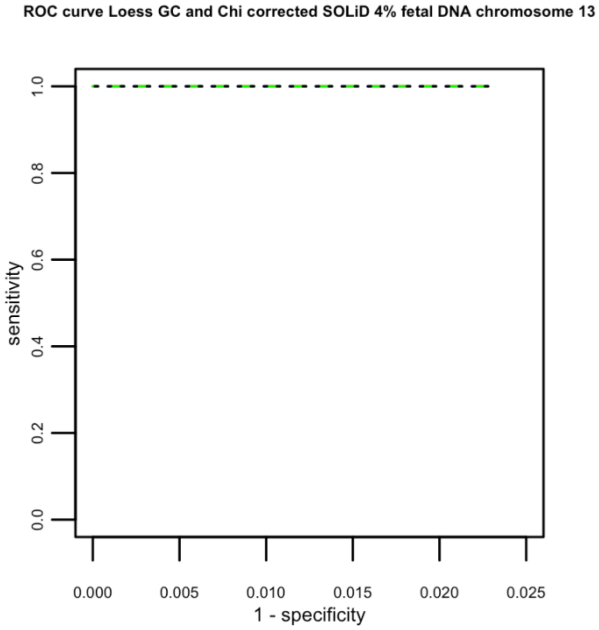

j1

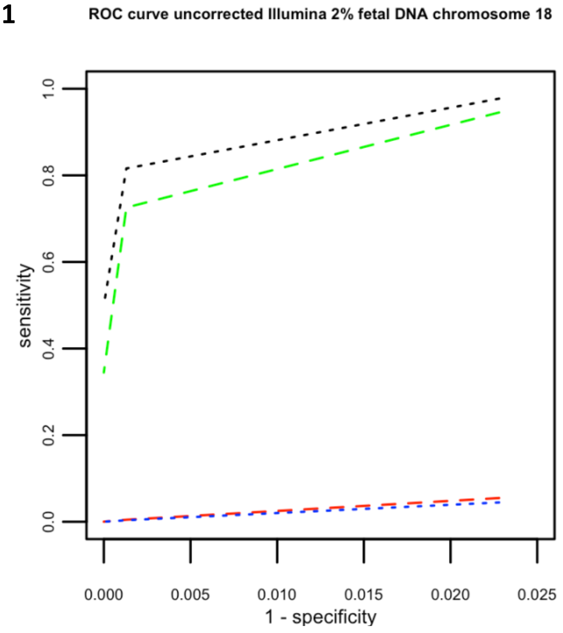

j2

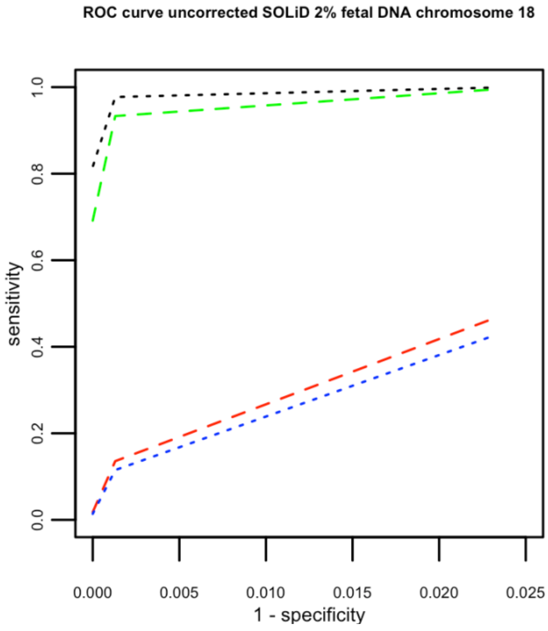

k1

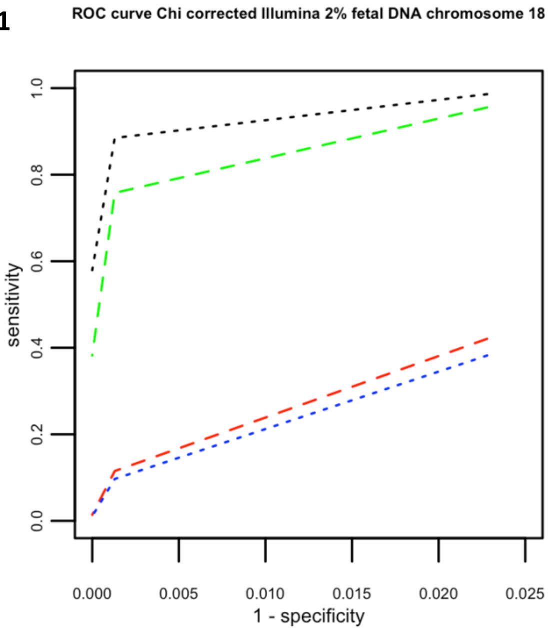

k2

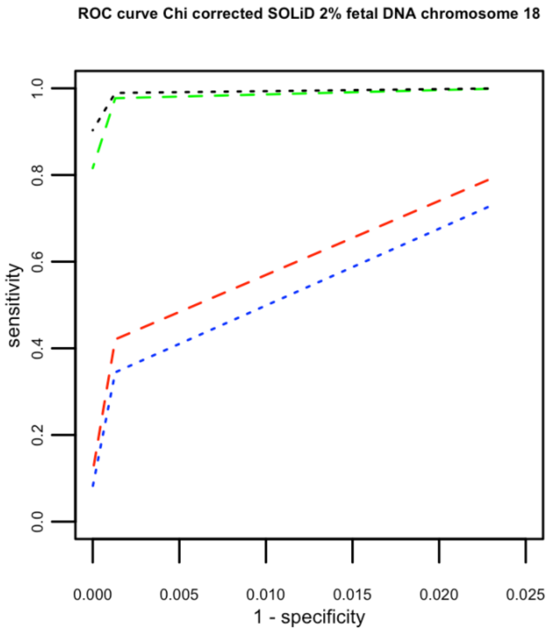

l1

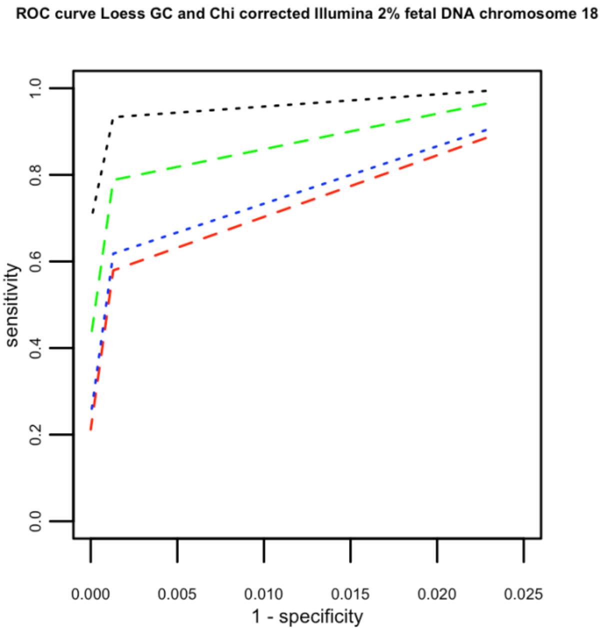

l2

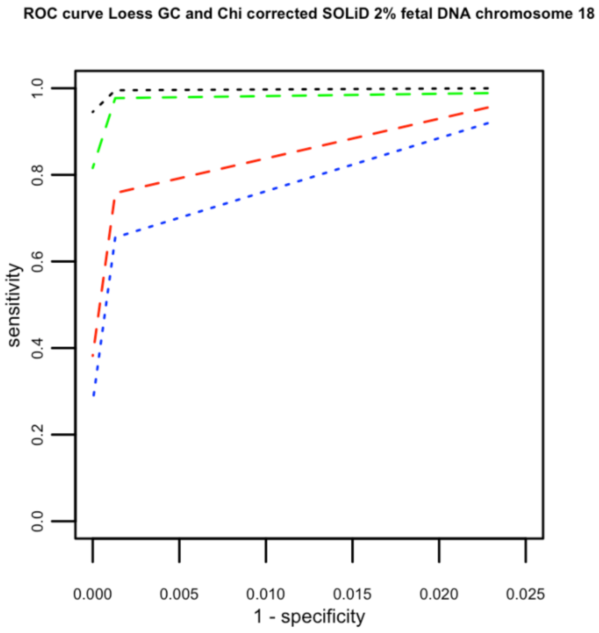

m1 ROC curve uncorrected Illumina 3% fetal DNA chromosome 18

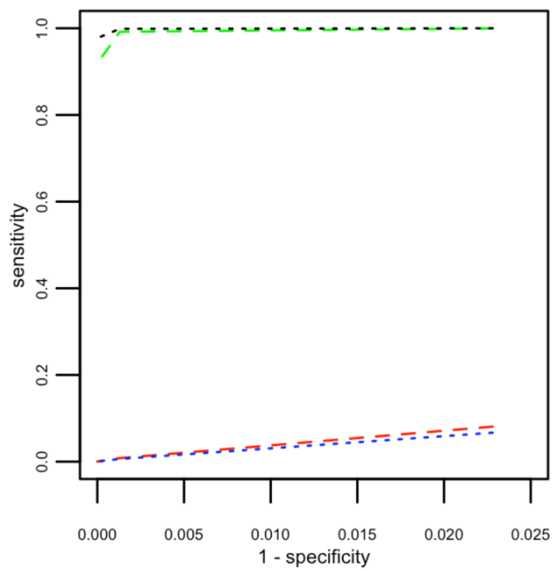

m2 ROC curve uncorrected SOLiD 3% fetal DNA chromosome 18

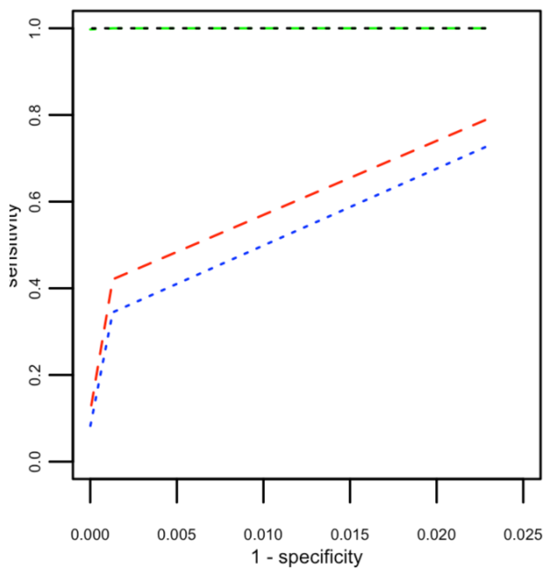

n1 ROC curve Chi corrected Illumina 3% fetal DNA chromosome 18

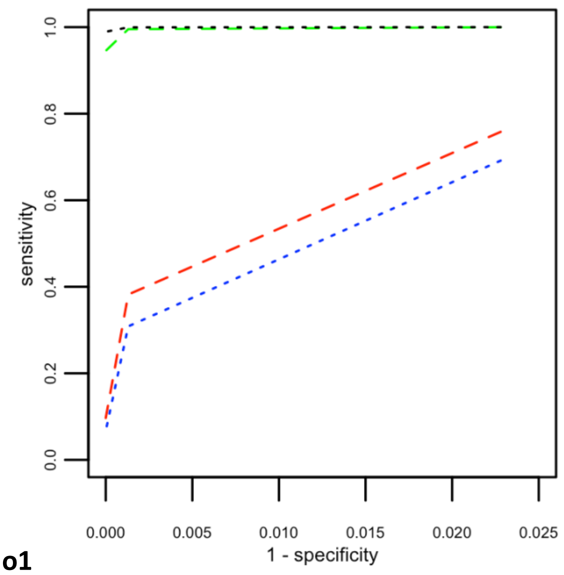

n2 ROC curve Chi corrected SOLiD 3% fetal DNA chromosome 18

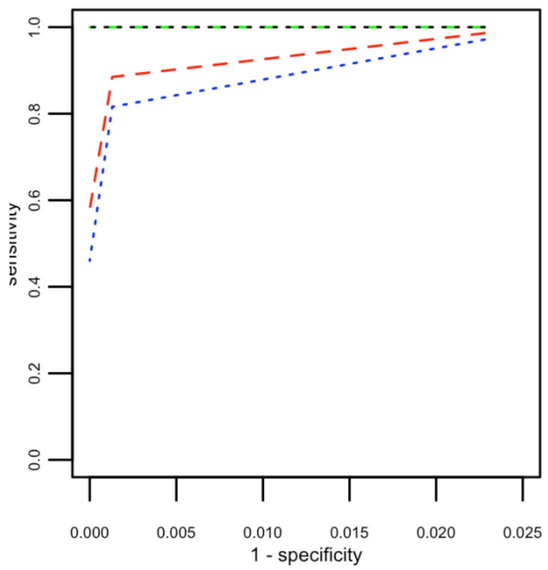

o1 ROC curve Loess GC and Chi corrected Illumina 3% fetal DNA chromosome 18

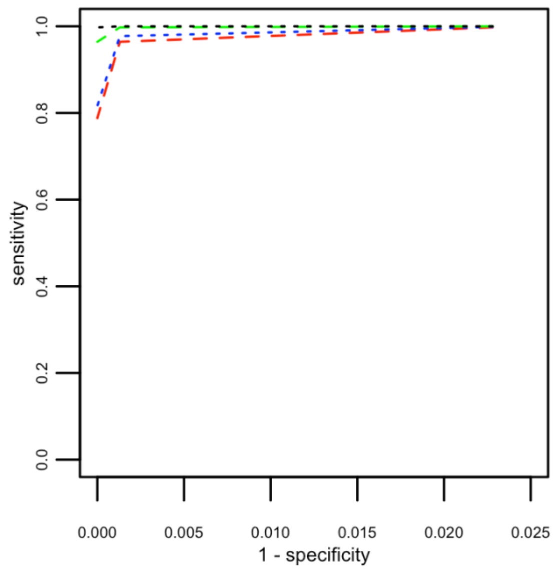

o2 ROC curve Loess GC and Chi corrected SOLiD 3% fetal DNA chromosome 18

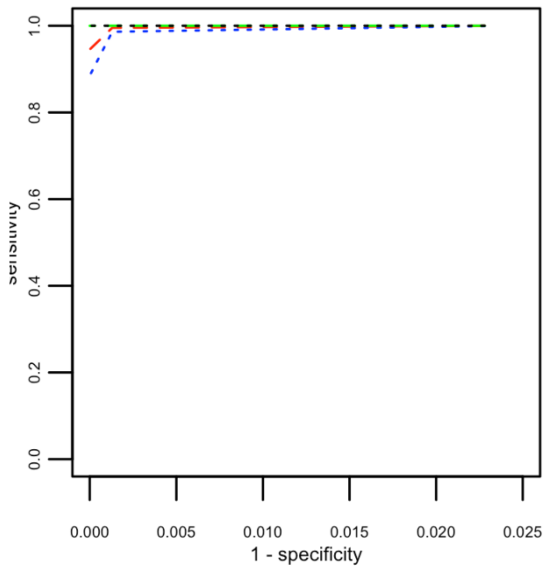

p1

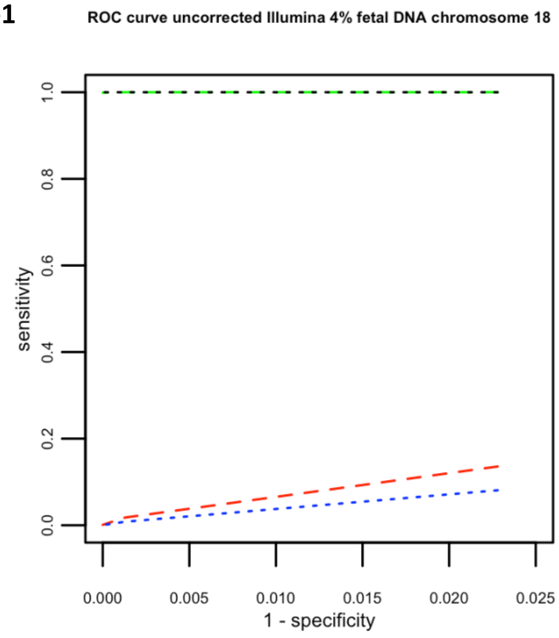

p2

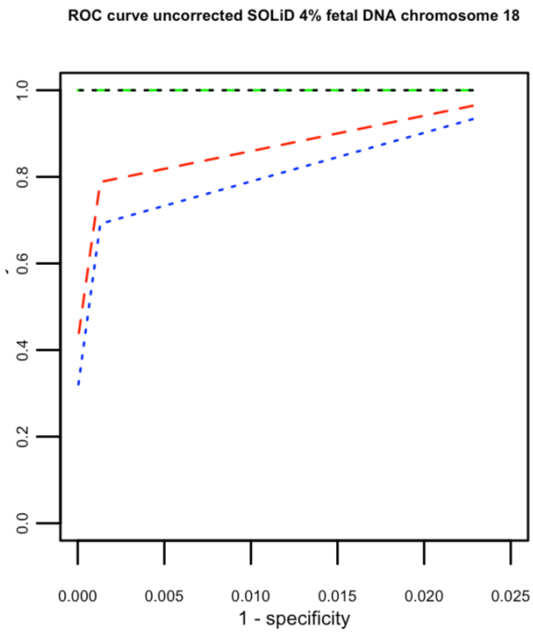

q1

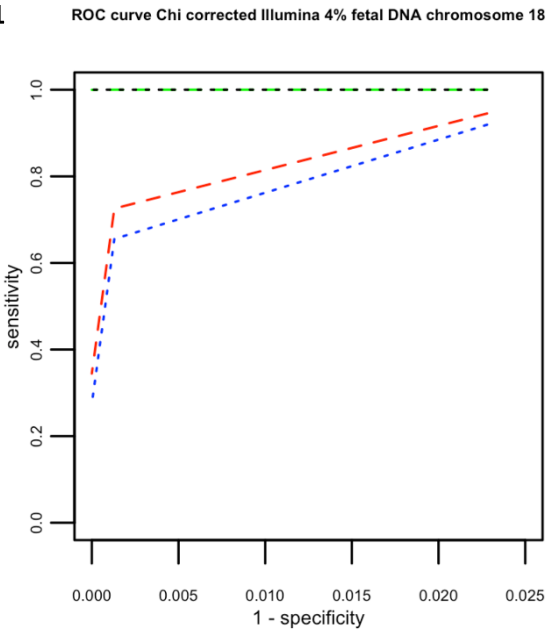

q2

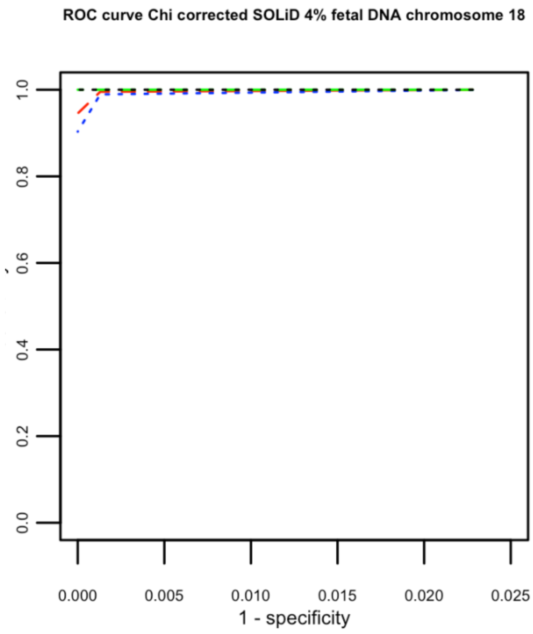

r1

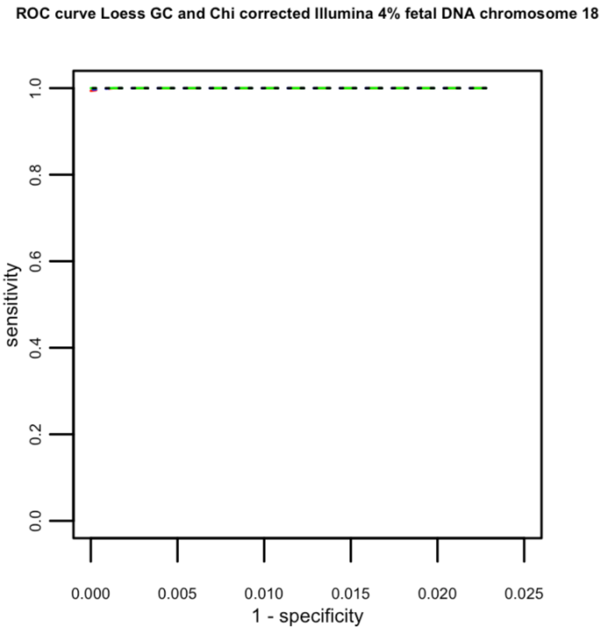

r2

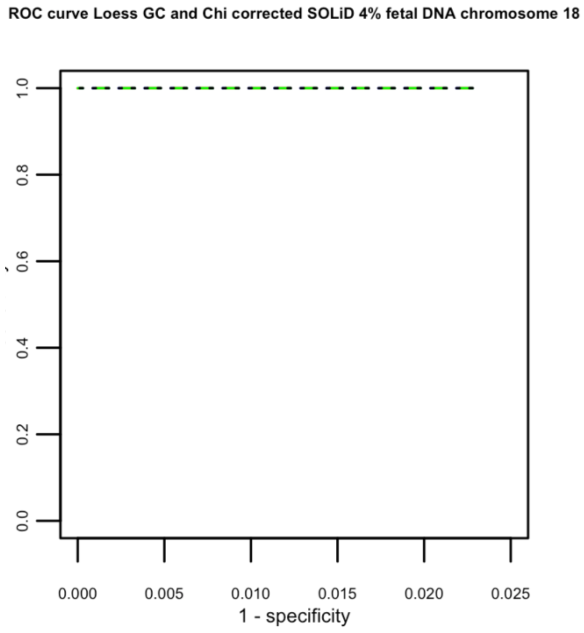

s1

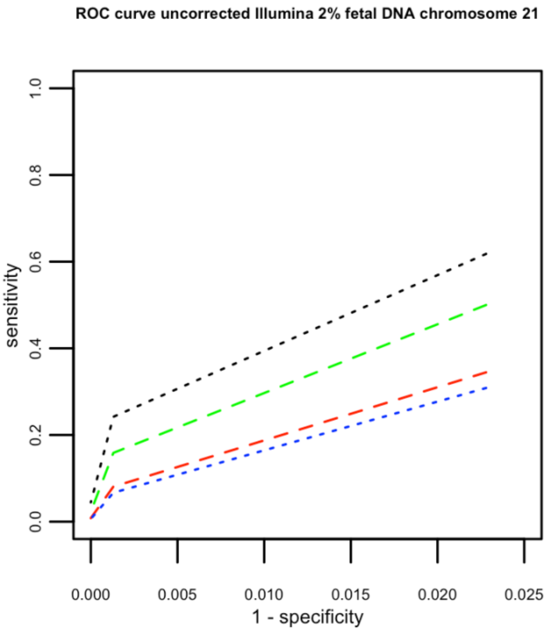

s2

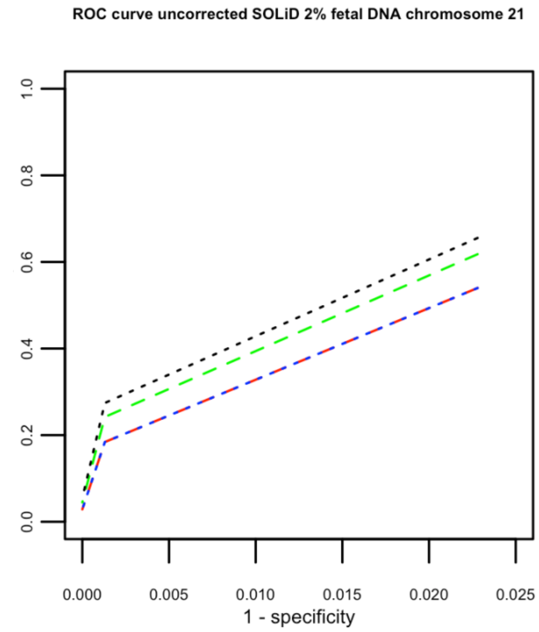

t1

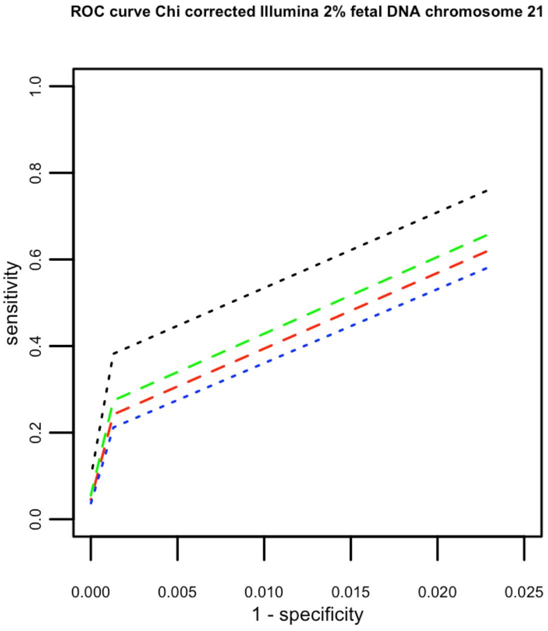

t2

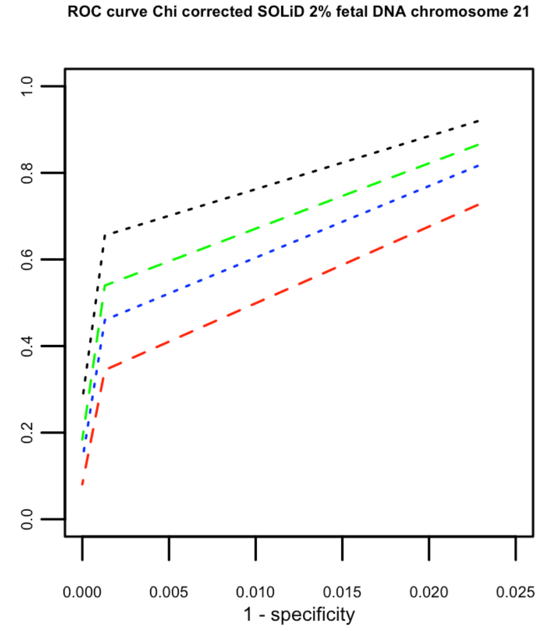

u1

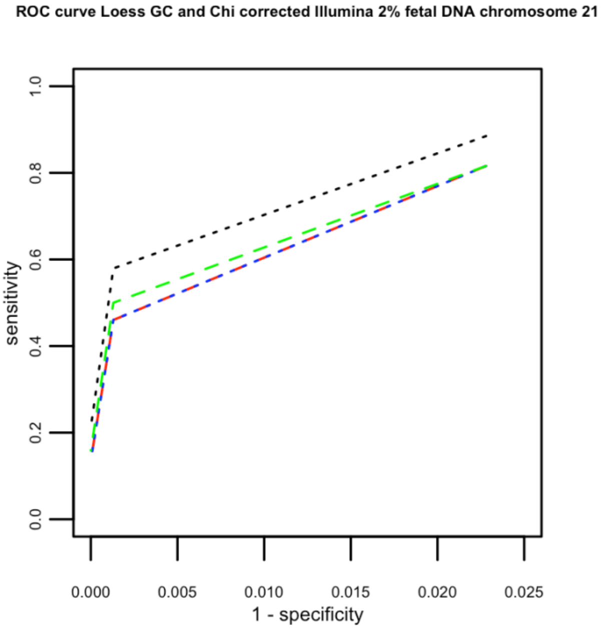

u2

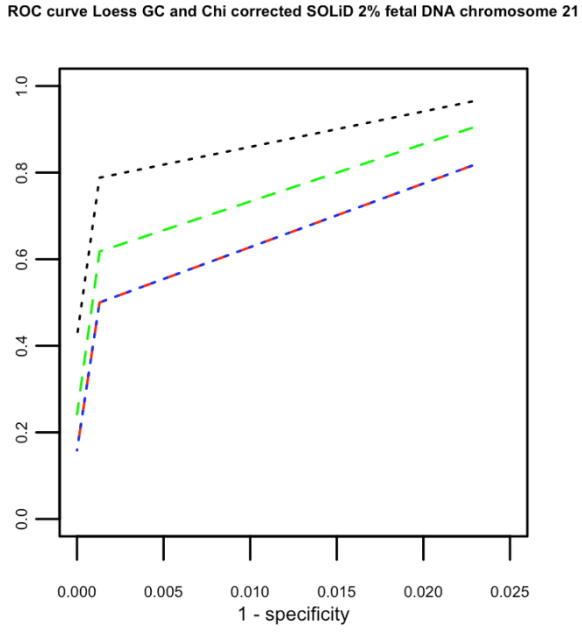

v1

ROC curve uncorrected Illumina 3% fetal DNA chromosome 21

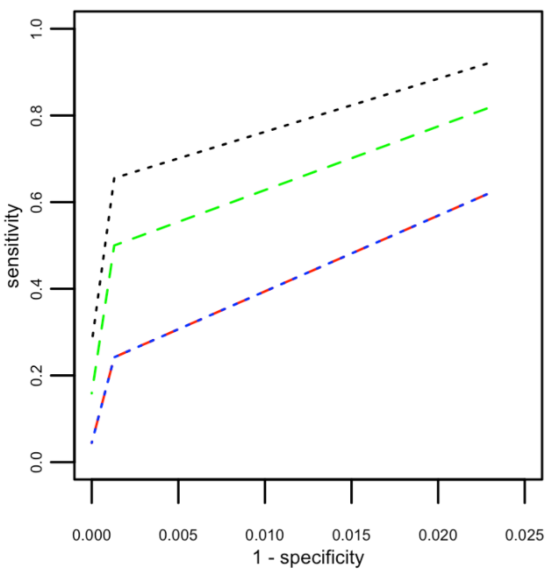

v2

ROC curve uncorrected SOLiD 3% fetal DNA chromosome 21

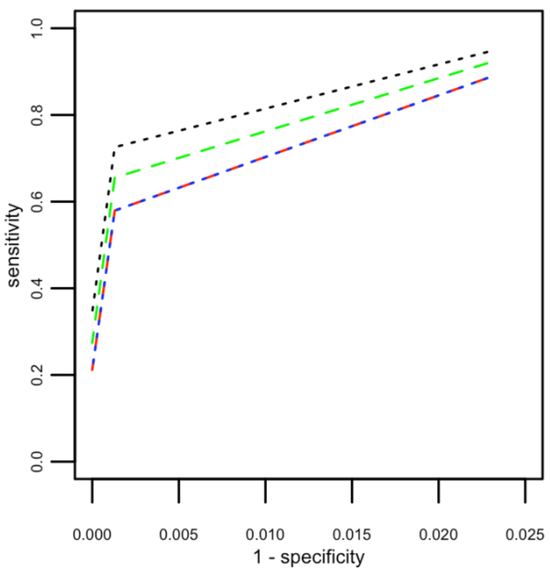

w1

ROC curve Chi corrected Illumina 3% fetal DNA chromosome 21

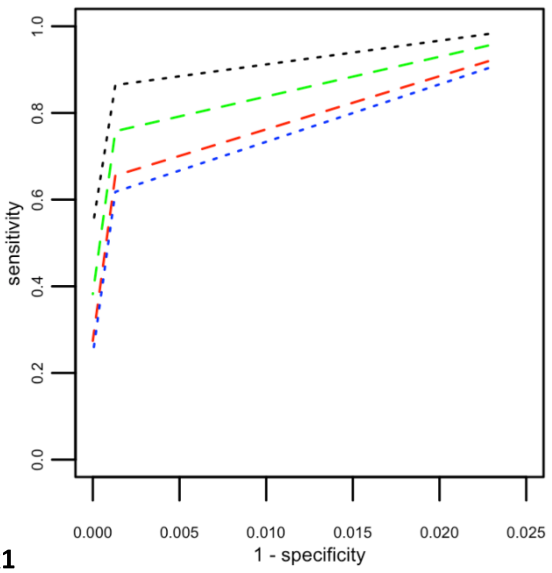

w2

ROC curve Chi corrected SOLiD 3% fetal DNA chromosome 21

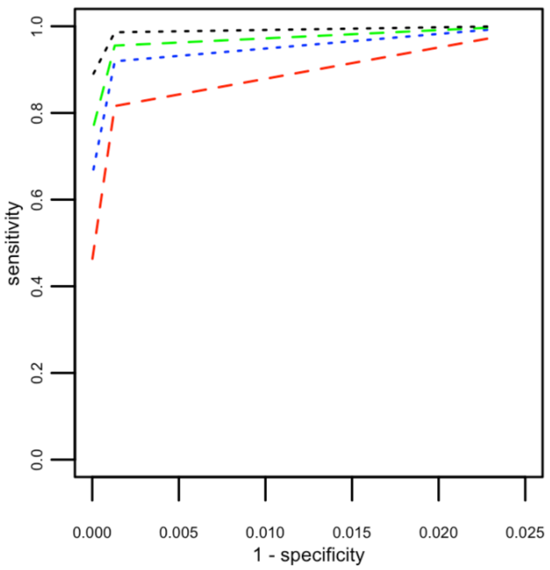

x1

ROC curve Loess GC and Chi corrected Illumina 3% fetal DNA chromosome 21

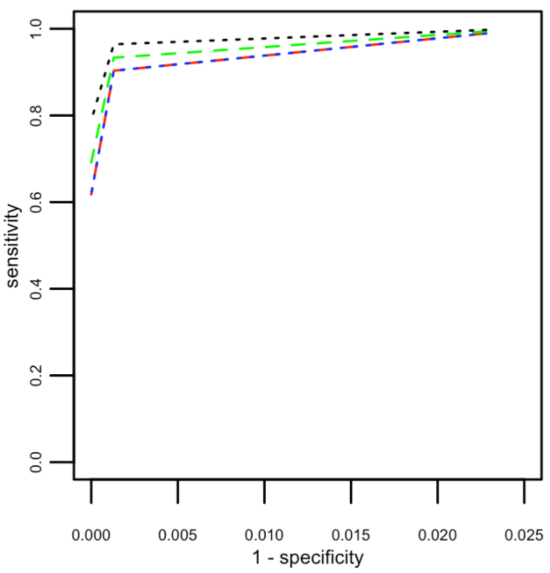

x2

ROC curve Loess GC and Chi corrected SOLiD 3% fetal DNA chromosome 21

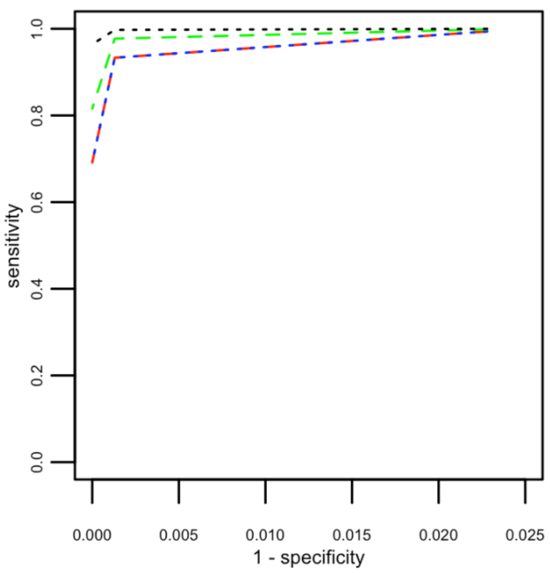

y1

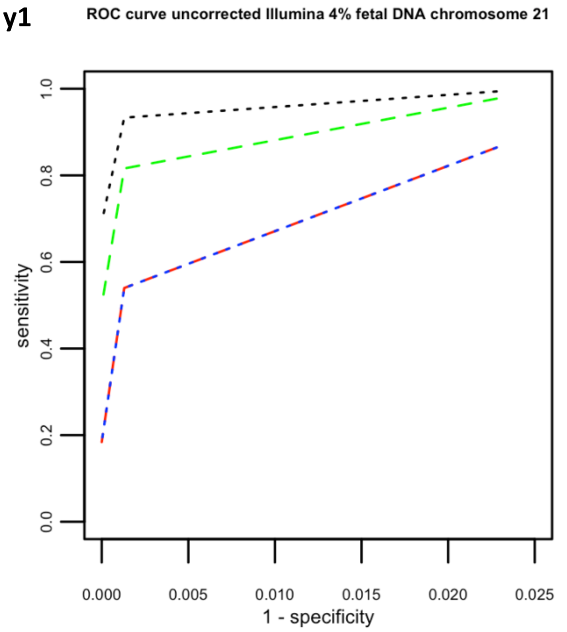

y2

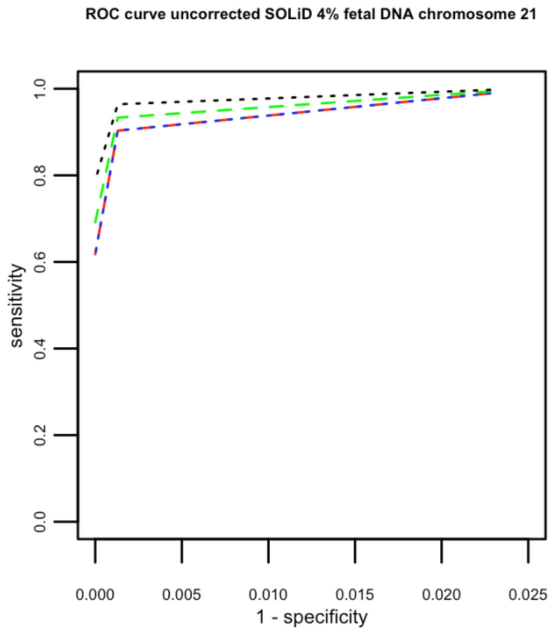

z1

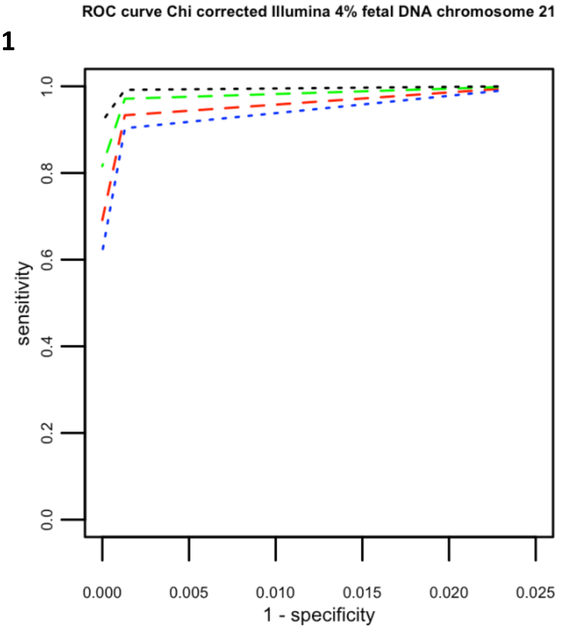

z2

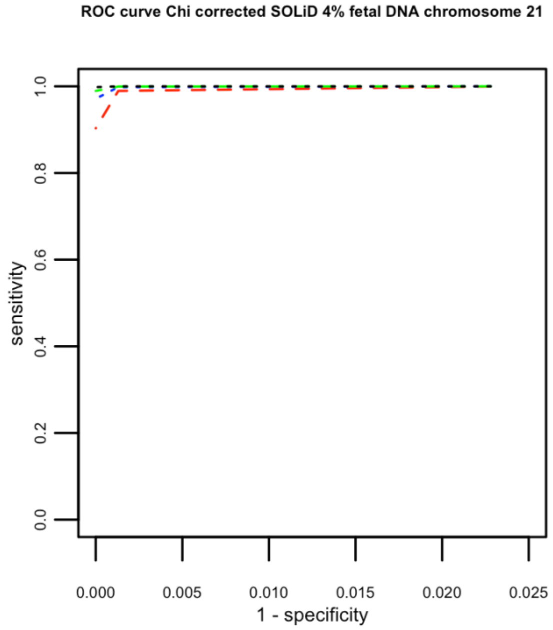

aa1

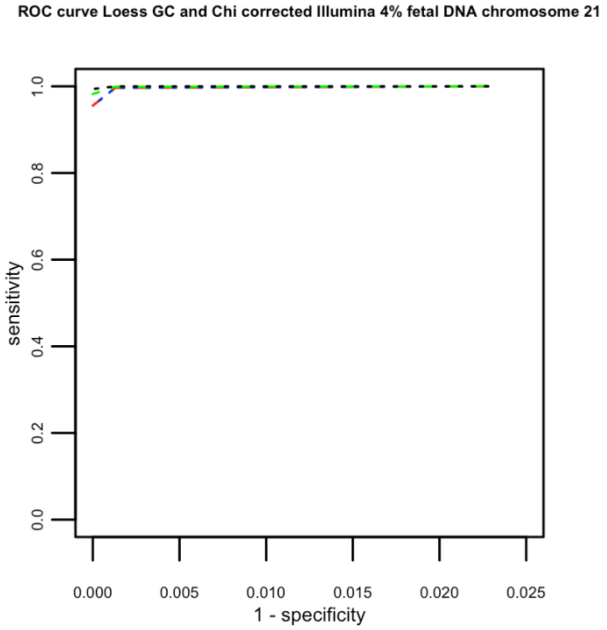

aa2

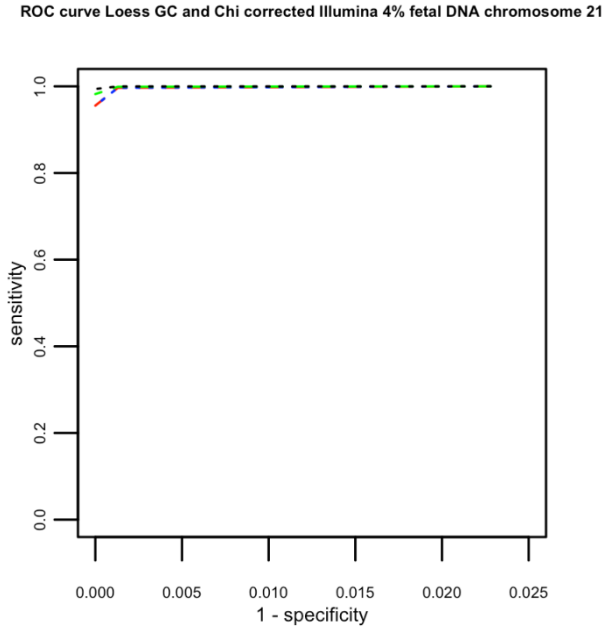

Figure S5.1: ROC curves (1- specificity shown up to 0.025) for uncorrected (a,d,g,j,m,p,s,v,y), χ^2VR corrected (b,e,h,k,n,q,t,w,z), and both LOESS GC and χ^2VR corrected (c,f,i,l,o,r,u,x,aa) data, shown for chromosomes 13 (a-i), 18 (j-r) and 21 (s-aa) for both Illumina (1) and SOLiD (2). Red line: Standard Z-score, blue line: MAD-based Z-score, green line: Normalized Chromosome Value, black line: Regression-based Z-score.

Supplement 6: Percentage cell-free fetal DNA trisomy samples

Based on the formula from Supplement 4, using the detected Z-score and the CV for the uncorrected, the χ^2VR corrected, the LOESS GC corrected and the both LOESS GC and χ^2VR corrected data for all four prediction methods (standard Z-score, MAD-based Z-score, Normalized Chromosome Value and Regression-based Z-score) the percentage cffDNA is calculated:

In short the formula is $\%cffDNA = 2 * CV * Z_{obs}$.

Based on the average of these 16 values an estimation was made of the percentage cffDNA present in these samples. These percentages are shown in **table S6.1**. Note that sample SOLiD_tri11 has an estimated percentage cffDNA of 0%. This is due to the very low, and in some cases negative, Z-scores for this sample. Note that sample Illumina_tri1 is missing. This is because this sample was processed differently and was not included in the study.

	Tri13	Tri18	Tri21			Tri13	Tri18	Tri21
SOLiD_tri1			13.6		SOLiD_tri26			11.5
SOLiD_tri2			16.4		SOLiD_tri27			21.5
SOLiD_tri3		6.3			SOLiD_tri28			12.2
SOLiD_tri4		8.2			SOLiD_tri29		8.8	
SOLiD_tri5			9.7		SOLiD_tri30		8.3	
SOLiD_tri6	17.3				SOLiD_tri31	8.3		
SOLiD_tri7	5.4				SOLiD_tri32			14.1
SOLiD_tri8	7.9				SOLiD_tri33	15.6		
SOLiD_tri9		5.4			SOLiD_tri34		5.7	
SOLiD_tri10			17.4		SOLiD_tri35			9.6
SOLiD_tri11		0.0			SOLiD_tri36		13.8	
SOLiD_tri12		11.6			SOLiD_tri37			5.8
SOLiD_tri13			16.9		SOLiD_tri38			5.0
SOLiD_tri14			15.1		SOLiD_tri39			12.3
SOLiD_tri15		7.6			SOLiD_tri40	5.9		
SOLiD_tri16			5.5		SOLiD_tri41			9.1
SOLiD_tri17		15.1			SOLiD_tri42			15.1
SOLiD_tri18			12.4		SOLiD_tri43		2.7	
SOLiD_tri19		5.2			Illumina_tri2			17.1
SOLiD_tri20			8.7		Illumina_tri3			14.2
SOLiD_tri21			8.1		Illumina_tri4			10.5
SOLiD_tri22			21.0		Illumina_tri5			6.3
SOLiD_tri23			21.5		Illumina_tri6			5.9
SOLiD_tri24			8.3		Illumina_tri7		12.1	
SOLiD_tri25			13.2		Illumina_tri8		11.5	

Table S6.1: Estimated percentages cffDNA in the positive control trisomy samples.

Supplement 7: Z-scores trisomy samples

	uncorrected				Chi corrected				LOESS GC corrected				LOESS GC and chi corrected			
	Trisomy 13				Trisomy 13				Trisomy 13				Trisomy 13			
	Zscore	MAD	NCV	RBZ	Zscore	MAD	NCV	RBZ	Zscore	MAD	NCV	RBZ	Zscore	MAD	NCV	RBZ
SOLiD_tri6	6.0	5.9	28.8	39.5	9.9	9.4	40.6	42.4	36.8	38.5	43.5	43.9	41.7	49.2	45.6	45.5
SOLiD_tri7	2.2	2.3	9.1	11.8	3.5	3.3	12.5	12.7	10.9	11.4	12.9	12.5	12.2	14.5	13.5	13.0
SOLiD_tri8	4.3	4.3	12.0	15.4	5.9	5.6	15.7	16.6	14.7	15.4	16.8	17.4	16.9	20.0	18.0	17.9
SOLiD_tri31	3.4	3.4	13.2	18.7	5.2	4.9	18.4	20.2	16.5	17.3	19.5	19.7	19.0	22.5	21.1	21.5
SOLiD_tri33	6.8	6.8	25.6	34.3	10.1	9.5	34.4	36.4	31.3	32.7	36.4	36.6	35.5	41.9	38.5	38.8
SOLiD_tri40	1.7	1.7	10.1	14.7	3.2	3.0	14.9	15.6	13.2	13.8	15.5	15.7	15.3	18.1	16.8	16.7

	Trisomy 18				Trisomy 18				Trisomy 18				Trisomy 18			
	Zscore	MAD	NCV	RBZ	Zscore	MAD	NCV	RBZ	Zscore	MAD	NCV	RBZ	Zscore	MAD	NCV	RBZ
Illumina_tri7	3.4	2.5	21.6	23.7	11.3	10.5	21.5	18.6	18.4	17.1	22.4	24.9	18.7	19.8	21.9	23.0
Illumina_tri8	2.7	2.0	21.5	23.5	10.4	9.6	20.9	21.2	18.0	16.7	21.9	24.0	18.3	19.3	21.6	23.7
SOLiD_tri3	7.1	6.5	14.5	15.9	9.4	8.8	15.1	15.0	8.3	7.8	14.3	16.1	10.5	9.9	16.0	16.5
SOLiD_tri4	6.5	6.0	18.8	21.3	10.6	10.0	21.4	22.7	12.7	12.0	18.3	20.0	16.0	15.0	20.7	23.3
SOLiD_tri9	5.9	5.4	12.2	13.0	8.2	7.7	13.0	12.1	7.8	7.4	12.2	12.1	10.0	9.4	13.6	13.2
SOLiD_tri11	-0.8	-0.7	0.1	0.1	-0.5	-0.4	0.7	0.2	0.7	0.6	1.0	1.2	0.6	0.5	1.0	-0.6
SOLiD_tri12	9.0	8.3	26.4	30.4	15.0	14.2	30.1	32.0	17.8	16.9	26.5	29.0	22.7	21.4	29.7	33.1
SOLiD_tri15	7.8	7.2	17.3	18.7	10.9	10.3	18.6	19.5	10.9	10.2	16.9	19.2	13.6	12.8	18.8	20.6
SOLiD_tri17	14.7	13.5	34.6	39.0	21.0	19.8	37.7	40.6	21.1	20.0	33.9	38.1	26.8	25.3	38.0	42.8
SOLiD_tri19	4.6	4.3	11.6	13.1	7.4	7.0	13.6	14.1	7.4	7.0	11.7	13.5	9.7	9.1	13.5	14.4
SOLiD_tri29	8.0	7.4	19.9	22.4	12.1	11.4	22.1	23.5	13.2	12.5	19.9	21.9	16.4	15.5	22.0	23.9
SOLiD_tri30	8.2	7.5	18.3	20.2	12.1	11.4	20.4	21.7	12.3	11.6	18.2	19.4	15.4	14.5	20.3	22.2
SOLiD_tri34	5.1	4.7	13.4	14.7	7.6	7.2	14.1	14.4	8.4	7.9	13.6	15.2	10.6	10.0	15.0	15.0
SOLiD_tri36	13.6	12.5	30.8	34.0	19.7	18.6	33.5	35.6	20.0	18.9	30.1	33.3	25.1	23.7	34.0	37.5
SOLiD_tri43	1.8	1.7	6.2	8.3	3.0	2.9	6.9	7.9	4.0	3.7	5.9	8.5	4.9	4.6	6.4	9.1

	Trisomy 21				Trisomy 21				Trisomy 21				Trisomy 21			
	Zscore	MAD	NCV	RBZ	Zscore	MAD	NCV	RBZ	Zscore	MAD	NCV	RBZ	Zscore	MAD	NCV	RBZ
Illumina_tri2	12.4	12.2	16.8	18.6	20.7	20.0	22.3	22.2	16.2	17.5	18.0	19.4	25.3	24.7	26.5	27.6
Illumina_tri3	10.4	10.3	14.4	14.3	17.0	16.4	18.5	16.2	13.7	14.8	15.3	15.4	21.9	21.4	22.9	23.3
Illumina_tri4	7.5	7.5	10.2	11.2	12.5	12.0	13.8	12.8	10.2	11.0	11.0	11.0	15.9	15.6	16.8	18.1
Illumina_tri5	4.4	4.4	5.7	7.3	8.0	7.7	8.5	8.4	6.2	6.6	6.0	6.8	9.2	9.0	9.4	10.9
Illumina_tri6	4.4	4.4	6.0	6.2	6.5	6.3	7.5	8.1	5.6	6.0	6.3	6.0	9.1	8.8	9.5	10.1
SOLiD_tri1	14.7	14.7	16.4	15.9	18.0	19.6	21.3	23.0	14.8	13.2	16.9	17.4	20.1	20.1	23.2	24.9
SOLiD_tri2	17.4	17.4	18.9	17.8	22.6	24.7	26.4	27.7	17.6	15.7	19.4	19.6	25.3	25.3	28.5	30.7
SOLiD_tri5	10.1	10.0	10.8	11.1	13.1	14.3	15.4	16.2	10.9	9.7	11.4	11.4	15.2	15.2	17.0	18.5
SOLiD_tri10	19.5	19.5	21.1	21.2	23.1	25.2	27.0	24.4	19.9	17.7	21.6	22.2	26.2	26.2	30.4	27.1

SOLiD_tri13	17.8	17.8	19.6	20.0	23.0	25.1	27.6	24.8	18.9	16.8	20.1	20.7	27.0	27.0	30.5	27.4
SOLiD_tri14	16.2	16.1	17.9	17.7	19.9	21.7	24.0	25.4	16.3	14.5	18.3	18.1	22.5	22.5	26.2	28.4
SOLiD_tri16	5.8	5.8	5.8	6.3	7.6	8.3	8.9	9.0	6.4	5.7	5.8	6.5	8.7	8.7	9.3	10.1
SOLiD_tri18	13.0	13.0	13.9	14.4	17.2	18.8	19.9	20.8	13.7	12.2	14.0	14.1	19.8	19.8	21.8	23.6
SOLiD_tri20	9.1	9.1	9.6	10.1	12.4	13.5	14.5	15.0	9.1	8.1	9.7	9.5	13.8	13.8	15.9	16.9
SOLiD_tri21	8.6	8.6	9.3	9.7	10.8	11.8	12.5	13.6	9.2	8.2	10.2	9.7	12.0	12.0	13.3	14.8
SOLiD_tri22	21.5	21.4	23.7	23.6	28.6	31.3	34.1	37.4	22.2	19.8	24.5	24.4	32.5	32.5	37.4	41.7
SOLiD_tri23	23.0	23.0	25.1	25.0	29.1	31.7	33.8	36.5	23.3	20.7	25.4	25.7	32.9	33.0	37.0	41.0
SOLiD_tri24	8.8	8.8	9.8	9.7	11.5	12.6	13.2	14.0	8.4	7.5	9.9	10.0	12.8	12.8	14.8	15.4
SOLiD_tri25	13.7	13.7	14.6	14.9	18.5	20.2	21.1	22.7	13.9	12.4	15.5	16.0	20.8	20.8	23.5	24.8
SOLiD_tri26	11.7	11.7	12.7	13.2	16.2	17.7	18.9	20.6	11.9	10.7	13.6	13.4	17.8	17.8	19.9	22.5
SOLiD_tri27	22.5	22.5	24.3	24.1	29.5	32.2	34.5	38.3	23.1	20.6	24.9	25.0	33.3	33.4	37.7	42.8
SOLiD_tri28	12.7	12.7	12.5	13.4	17.4	19.0	18.9	19.5	13.5	12.0	14.4	14.9	19.3	19.3	21.9	22.1
SOLiD_tri32	15.4	15.4	16.7	15.7	19.2	20.9	22.3	22.1	15.5	13.8	16.7	17.0	22.0	22.0	24.1	24.6
SOLiD_tri35	9.8	9.8	10.6	11.3	12.9	14.1	15.2	17.2	10.5	9.4	11.6	11.3	14.8	14.8	16.9	19.3
SOLiD_tri37	5.7	5.7	6.8	6.4	7.9	8.6	9.6	11.4	5.9	5.3	6.8	6.8	8.8	8.8	10.1	12.4
SOLiD_tri38	4.6	4.6	5.1	4.9	7.4	8.1	8.5	9.7	4.7	4.2	5.5	5.4	8.7	8.7	9.8	11.4
SOLiD_tri39	14.0	14.0	14.8	15.3	16.5	18.0	19.1	17.5	14.2	12.6	14.9	14.9	18.7	18.7	20.6	19.6
SOLiD_tri41	9.9	9.9	10.7	10.3	11.7	12.8	14.3	13.9	10.6	9.5	11.3	11.4	13.9	13.9	16.0	15.6
SOLiD_tri42	17.5	17.5	18.8	19.4	20.2	22.1	24.5	18.8	18.1	16.1	18.7	18.4	22.5	22.5	25.2	19.9

Table S7.1: All Z-scores for the trisomy samples for combinations of variation reduction (χ2VR and LOESS GC) and trisomy prediction methods (Z-score, MAD-based Zscore, Normalized Chromosome Value and Regression-based Z-score) for Illumina and SOLiD data.

Supplement 8: False positive and false negative results

	Illumina								SOLiD							
Trisomy 13	TN				FP				TN				FP			
	Zscore	MAD	NCV	RBZ	Zscore	MAD	NCV	RBZ	Zscore	MAD	NCV	RBZ	Zscore	MAD	NCV	RBZ
uncorrected	142	142	142	142	0	0	0	0	128	128	128	127	0	0	0	1
chi corrected	142	142	142	142	0	0	0	0	128	128	126	126	0	0	2	2
LOESS GC corrected	142	142	142	142	0	0	0	0	128	128	126	126	0	0	2	2
LOESS GC and chi corrected	142	142	142	142	0	0	0	0	127	126	127	126	1	2	1	2

Trisomy 18	TN				FP				TN				FP			
	Zscore	MAD	NCV	RBZ	Zscore	MAD	NCV	RBZ	Zscore	MAD	NCV	RBZ	Zscore	MAD	NCV	RBZ
uncorrected	142	142	142	142	0	0	0	0	128	128	126	128	0	0	2	0
chi corrected	142	142	142	141	0	0	0	1	127	128	128	127	1	0	0	1
LOESS GC corrected	142	142	142	142	0	0	0	0	128	128	127	128	0	0	1	0
LOESS GC and chi corrected	142	142	142	141	0	0	0	1	128	128	127	128	0	0	1	0

Trisomy 21	TN				FP				TN				FP			
	Zscore	MAD	NCV	RBZ	Zscore	MAD	NCV	RBZ	Zscore	MAD	NCV	RBZ	Zscore	MAD	NCV	RBZ
uncorrected	142	142	141	141	0	0	1	1	128	128	128	128	0	0	0	0
chi corrected	141	142	142	142	1	0	0	0	128	128	128	128	0	0	0	0
LOESS GC corrected	142	142	141	142	0	0	1	0	128	128	128	127	0	0	0	1
LOESS GC and chi corrected	142	142	142	142	0	0	0	0	128	128	128	128	0	0	0	0

Table S8.1: True negative and false positive results for combinations of variation reduction (χ^2VR and LOESS GC) and trisomy prediction methods (Z-score, MAD-based Zscore, Normalized Chromosome Value and Regression-based Z-score) for Illumina and SOLiD data.

Chr13: false positives						
	Sample No		Zscore	MAD	NCV	RBZ
SOLiD	37	chi corrected	1.52	1.44	3.04	3.14
SOLiD	37	LOESS GC corrected	2.90	3.00	3.19	3.59
SOLiD	37	LOESS GC and chi corrected	2.63	3.13	2.82	3.21
SOLiD	50	uncorrected	-0.14	-0.09	2.18	4.47
SOLiD	50	chi corrected	0.14	0.14	4.12	5.17
SOLiD	50	LOESS GC corrected	2.43	2.54	4.36	4.19
SOLiD	50	LOESS GC and chi corrected	3.05	3.55	4.22	5.12

Chr18: false positives						
	Sample No		Zscore	MAD	NCV	RBZ
Illumina	23	LOESS GC and chi corrected	0.84	0.99	1.84	3.04
Illumina	54	chi corrected	0.59	0.42	2.70	3.37
SOLiD	25	uncorrected	-1.40	-1.25	3.02	1.96
SOLiD	83	uncorrected	2.85	2.57	3.12	2.85
SOLiD	83	chi corrected	3.13	2.90	2.77	3.28
SOLiD	83	LOESS GC corrected	1.78	1.59	3.16	2.60
SOLiD	83	LOESS GC and chi corrected	2.16	1.93	3.44	2.50

Chr21: false positives						
	Sample No		Zscore	MAD	NCV	RBZ
Illumina	10	uncorrected	1.73	1.74	3.12	3.03
Illumina	10	chi corrected	3.20	2.95	2.75	1.59
Illumina	10	LOESS GC corrected	2.63	2.74	3.08	2.49
SOLiD	26	LOESS GC corrected	1.58	1.43	2.29	3.22

Table S8.2: Z-scores of all trisomy prediction methods for samples and variation reduction methods showing a false positive result for one or more of the trisomy prediction methods.

Chr13						
	Sample No		Prediction set	Z score	CV(%)	Regression model
SOLiD	37	chi corrected	1	3.14	0.20	0.058 + 0.338 Chr4R + 0.4431 Chr5F - 0.6218 Chr20R - 0.5101 Chr12F
			2	2.98	0.20	0.1128 + 0.221 Chr4F - 0.4654 Chr20F - 0.4139 Chr1F - 0.4033Chr16R
			3	2.26	0.20	0.1824 - 1.1462 Chr22F - 0.6591 Chr1R - 0.6834 Chr16F - 0.3875Chr5R
			4	1.68	0.23	0.1639 - 1.4983 Chr22R - 0.6946 Chr11F -0.7605 Chr15F - 0.4033 Chr12R
SOLiD	37	LOESS GC corrected	1	3.59	0.19	0.143 - 0.4397 Chr12F - 0.3078 Chr1F - 0.4031 Chr16F - 0.2494 Chr10R
			2	2.55	0.20	-0.0045 + 0.4038 Chr8R + 0.2854 Chr4R + 0.5004 Chr14R + 0.2758 Chr6R
			3	2.95	0.20	0.1021 + 0.2043 Chr5F - 0.2531 Chr1R - 0.3516 Chr16R - 0.2572 Chr10F
			4	2.87	0.21	0.1029 - 0.4415 Chr12R + 0.1201 Chr4F - 0.6052 Chr22R - 0.209 Chr11R
SOLiD	37	LOESS GC and chi corrected	1	3.21	0.18	0.1613 - 0.4651 Chr12F - 0.2781 Chr1F - 0.5081 Chr16F - 0.2672 Chr2R
			2	2.83	0.18	0.1527 - 0.3516 Chr12R - 0.2709 Chr1R - 0.4253 Chr16R - 0.2643 Chr2F
			3	3.05	0.19	0.0409 + 0.2117 Chr4R + 0.3092 Chr5F - 0.1787 Chr11R + 0.2495 Chr14R
			4	2.59	0.19	0.0447 + 0.3886 Chr8R - 0.2596 Chr20F + 0.1245 Chr4F + 0.1151 Chr6R
SOLiD	50	uncorrected	1	4.47	0.20	0.1047 + 0.3937Chr4R - 0.601 Chr16F - 0.2773 Chr1F - 0.4079 Chr10F
			2	3.33	0.22	0.1005 + 0.3367 Chr4F - 0.5507 Chr16R - 0.3291 Chr1R - 0.3902 Chr20R
			3	2.76	0.25	0.2081 - 1.5333 Chr20F - 0.9201 Chr15F - 0.9796 Chr12F -0.4884 Chr11R

			4	2.65	0.25	0.0926 - 0.8416 Chr22F - 0.5454 Chr10R - 0.6478 Chr11F + 0.7197 Chr5F
SOLiD	50	chi corrected	1	5.17	0.19	0.1225 + 0.2787 Chr4R -0.8094 Chr16F - 0.3861 Chr1R - 0.1728 Chr2R
			2	4.29	0.20	0.0665 + 0.3618 Chr4F + 0.2821 Chr6F - 0.3406 Chr1F - 0.3681 Chr16R
			3	3.41	0.21	0.1741 - 1.1141 Chr20F -1.0274 Chr15F -0.5911 Chr12F -0.2952 Chr11F
			4	3.70	0.21	0.074 -0.8074 Chr22F - 0.528 Chr11R + 0.6276 Chr5F - 0.502 Chr20R
SOLiD	50	LOESS GC corrected	1	4.19	0.19	0.0581 - 0.3925 Chr12F + 0.3667 Chr4R + 0.3793 Chr14R - 0.1934 Chr16F
			2	4.62	0.20	0.0207 + 0.3053 Chr8R + 0.2495 Chr4F + 0.482 Chr6F - 0.16 Chr1F
			3	3.97	0.19	0.0799 + 0.4557 Chr5F - 0.3635 Chr11R - 0.3116 Chr12R - 0.1804 Chr16R
			4	3.46	0.20	0.1329 - 0.1604 Chr5R - 0.3202 Chr1R - 0.2967 Chr19F - 0.3721 Chr10R
SOLiD	50	LOESS GC and chi corrected	1	5.12	0.17	0.1695 - 0.4492 Chr12F - 0.3222 Chr1R - 0.7131 Chr16F - 0.2702 Chr2R
			2	4.22	0.18	0.0523 - 0.3785 Chr12R + 0.2397 Chr4R + 0.3479 Chr14R + 0.227 Chr6F
			3	3.67	0.18	0.1486 - 0.3318 Chr1F - 0.6403 Chr16R - 0.2186 Chr2F -0.144 Chr11R
			4	4.59	0.19	0.0822 + 0.1523 Chr4F + 0.1784 Chr8R - 0.3631 Chr11F - 0.3471 Chr17R

Chr18						
	Sample No		Prediction set	Z score	CV(%)	Regression coefficients
Illumina	23	LOESS GC and chi corrected	1	3.04	0.23	0.0714 + 0.2577 Chr4F + 0.1437 Chr8R -0.3443 Chr19F - 0.3952 Chr1F
			2	1.20	0.23	0.1362 - 0.4921 Chr1R - 0.5043 Chr19R - 0.2539 Chr11R - 0.3119 Chr10R
			3	1.13	0.27	-0.0168 + 0.3766 Chr8F + 0.3394 Chr4R + 0.3139 Chr10F + 0.2215 Chr5R
			4	0.61	0.27	0.1328 - 0.6516 Chr17F - 0.4544 Chr11F -0.2762 Chr6F -0.2364 Chr3F
Illumina	54	chi corrected	1	3.37	0.27	0.0876 - 0.4045 Chr17F - 0.4098 Chr12F + 0.3689 Chr10F - 0.2007 Chr1F
			2	2.18	0.27	0.0905 - 0.2826 Chr17R + 0.8149 Chr22R - 0.3518 Chr19F - 0.2983 Chr1R
			3	2.35	0.27	0.0613 + 0.1255 Chr5R - 0.4593 Chr12R + 0.2676 Chr8F - 0.1357 Chr19R
			4	1.95	0.27	-0.0024 + 0.3427 Chr8R + 0.238 Chr4F + 0.503 Chr20F + 0.1821 Chr2F
SOLiD	83	chi corrected	1	3.28	0.18	0.036 + 0.3034 Chr5R + 0.4129 Chr8R -0.2172 Chr12R - 0.1046 Chr1R
			2	2.76	0.18	0.1207 - 0.366 Chr17F - 0.3922 Chr3F -0.2067 Chr1F - 0.2838 Chr19R
			3	3.12	0.17	0.0394 - 0.2558 Chr17R + 0.7371 Chr16F + 0.2105 Chr4R - 0.4806 Chr19F
			4	3.44	0.21	0.0148 + 0.1797 Chr5F + 0.5151 Chr8F + 0.3109 Chr14F - 0.18 Chr15F

Chr21						
	Sample No		Prediction set	Z score	CV(%)	Regression coefficients
Illumina	10	uncorrected	1	3.03	0.43	0.0857 -0.2346 Chr1F -0.3978 Chr3F -0.1676 Chr16F - 0.1507 Chr15F
			2	2.14	0.47	0.0605 -0.1517 Chr1R -0.344 Chr3R + 0.0969 Chr4F -0.0727 Chr16R
			3	3.57	0.50	0.0302 + 0.1485 Chr7R - 0.0548 Chr15R - 0.307 Chr12F + 0.0956 Chr8R
			4	3.63	0.51	0.0338 + 0.1946 Chr8F - 0.2307 Chr12R - 0.1285 Chr10R + 0.0345 Chr19R
SOLiD	26	LOESS GC corrected	1	3.22	0.39	0.0393 + 0.2247 Chr16F + 0.249 Chr20R -0.4209 Chr9R - 0.0883 Chr1R
			2	2.37	0.40	0.0581 - 0.1378 Chr6R - 0.4135 Chr14F - 0.2161 Chr3F + 0.2877 Chr20F
			3	2.43	0.41	0.0385 -0.2197 Chr6F -0.3005 Chr12F + 0.2131 Chr16R + 0.2139 Chr10F
			4	1.73	0.42	0.0571 + -0.176 Chr1F + 0.1022 Chr11R - 0.2862 Chr14R - 0.1622 Chr4R

Table S8.3: Results from additional RBZ models for samples with a false-positive result in the first RBZ model.

	Illumina								SOLiD							
Trisomy 13	TP				FN				TP				FN			
	Zscore	MAD	NCV	RBZ	Zscore	MAD	NCV	RBZ	Zscore	MAD	NCV	RBZ	Zscore	MAD	NCV	RBZ
uncorrected	0	0	0	0	0	0	0	0	4	4	6	6	2	2	0	0
chi corrected	0	0	0	0	0	0	0	0	6	6	6	6	0	0	0	0
LOESS GC corrected	0	0	0	0	0	0	0	0	6	6	6	6	0	0	0	0
LOESS GC and chi corrected	0	0	0	0	0	0	0	0	6	6	6	6	0	0	0	0

Trisomy 18	TP				FN				TP				FN			
	Zscore	MAD	NCV	RBZ	Zscore	MAD	NCV	RBZ	Zscore	MAD	NCV	RBZ	Zscore	MAD	NCV	RBZ
uncorrected	1	0	2	2	1	2	0	0	11	11	12	12	2	2	1	1
chi corrected	2	2	2	2	0	0	0	0	12	11	12	12	1	2	1	1
LOESS GC corrected	2	2	2	2	0	0	0	0	12	12	12	12	1	1	1	1
LOESS GC and chi corrected	2	2	2	2	0	0	0	0	12	12	12	12	1	1	1	1

Trisomy 21	TP				FN				TP				FN			
	Zscore	MAD	NCV	RBZ	Zscore	MAD	NCV	RBZ	Zscore	MAD	NCV	RBZ	Zscore	MAD	NCV	RBZ
uncorrected	5	5	5	5	0	0	0	0	24	24	24	24	0	0	0	0
chi corrected	5	5	5	5	0	0	0	0	24	24	24	24	0	0	0	0
LOESS GC corrected	5	5	5	5	0	0	0	0	24	24	24	24	0	0	0	0
LOESS GC and chi corrected	5	5	5	5	0	0	0	0	24	24	24	24	0	0	0	0

Table S8.4: True positive and false negative results for combinations of variation reduction (χ2VR and LOESS GC) and trisomy prediction methods (Z-score, MAD-based Zscore, Normalized Chromosome Value and Regression-based Z-score) for Illumina and SOLiD data.

Chr13						
	Sample No		Zscore	MAD	NCV	RBZ
SOLiD	tri7	uncorrected	2.25	2.28	9.08	11.76
SOLiD	tri40	uncorrected	1.70	1.74	10.11	14.70

Chr18						
	Sample No		Zscore	MAD	NCV	RBZ
Illumina	tri7	uncorrected	3.44	2.53	21.59	23.71
Illumina	tri8	uncorrected	2.70	2.01	21.49	23.52
SOLiD	tri11	uncorrected	-0.82	-0.71	0.11	0.09
SOLiD	tri11	chi corrected	-0.47	-0.44	0.70	0.18
SOLiD	tri11	LOESS GC corrected	0.72	0.58	0.97	1.25
SOLiD	tri11	LOESS GC and chi corrected	0.62	0.50	0.99	-0.59
SOLiD	tri43	uncorrected	1.82	1.71	6.18	8.33
SOLiD	tri43	chi corrected	3.04	2.88	6.86	7.94

Table S8.5: Z-scores of all trisomy prediction methods for samples and variation reduction methods showing a false negative result for one or more of the trisomy prediction methods.

Supplement 9: Match QC values and statistics

This supplement shows the Match QC values of all Illumina samples, using 85 Illumina control group samples as a training set. The remaining 57 control group samples were used as a matching test set and 34 plasma samples isolated using a single centrifugation step as a non-matching set **(Table S9.1)**. A summary of the statistics of the three groups is shown in **Table S9.2**. Density plots of Z-scores belonging to the groups are shown in **Figure S9.1.**

uncorrected			chi corrected			LOESS GC corrected			LOESS GC and chi corrected		
training set	test set	single centrifugation samples	training set	test set	single centrifugation samples	training set	test set	single centrifugation samples	training set	test set	single centrifugation samples
4.4E-05	3.5E-05	8.6E-05	8.9E-06	7.3E-06	2.0E-05	7.2E-07	6.8E-07	1.2E-05	3.1E-07	3.3E-07	1.1E-05
2.7E-05	5.0E-05	2.5E-05	5.9E-06	1.0E-05	5.9E-06	7.0E-07	6.4E-07	3.6E-05	3.9E-07	2.9E-07	3.7E-05
3.0E-05	3.4E-05	1.0E-04	6.7E-06	7.2E-06	2.5E-05	7.3E-07	1.1E-06	2.0E-05	3.8E-07	5.0E-07	2.1E-05
4.5E-05	4.8E-05	2.7E-05	9.2E-06	9.9E-06	7.1E-06	7.0E-07	1.0E-06	1.2E-05	3.1E-07	4.4E-07	1.1E-05
7.6E-05	3.6E-05	5.9E-05	1.5E-05	7.4E-06	1.6E-05	8.1E-07	8.2E-07	5.7E-06	5.0E-07	3.2E-07	5.6E-06
2.9E-05	5.5E-05	4.6E-05	6.5E-06	1.1E-05	1.4E-05	7.6E-07	1.1E-06	4.3E-06	3.7E-07	4.4E-07	3.9E-06
7.4E-05	8.9E-05	3.6E-05	1.5E-05	1.7E-05	1.0E-05	8.2E-07	7.9E-07	5.5E-06	5.3E-07	3.3E-07	5.3E-06
7.6E-05	4.8E-05	3.6E-05	1.5E-05	9.7E-06	9.6E-06	7.6E-07	8.0E-07	3.4E-06	5.1E-07	4.0E-07	3.1E-06
6.1E-05	4.1E-05	2.8E-05	1.3E-05	8.7E-06	7.8E-06	6.8E-07	7.2E-07	9.5E-06	3.8E-07	4.4E-07	8.9E-06
4.7E-05	4.2E-05	8.8E-05	9.9E-06	8.8E-06	2.1E-05	1.5E-06	6.7E-07	1.4E-05	5.0E-07	3.2E-07	1.4E-05
3.4E-05	3.8E-05	2.4E-05	7.0E-06	8.3E-06	6.2E-06	1.3E-06	9.8E-07	1.8E-05	9.4E-07	5.6E-07	1.9E-05
4.6E-05	4.0E-05	2.5E-05	9.4E-06	9.0E-06	6.6E-06	7.9E-07	6.8E-07	1.6E-05	5.3E-07	3.4E-07	1.7E-05
3.2E-05	3.2E-05	4.1E-05	6.8E-06	7.0E-06	7.8E-06	2.4E-06	6.1E-07	7.6E-05	1.1E-06	3.2E-07	8.0E-05
4.6E-05	4.0E-05	6.6E-05	9.8E-06	8.3E-06	1.7E-05	1.2E-06	7.9E-07	5.9E-06	4.8E-07	2.8E-07	6.2E-06
8.4E-05	5.0E-05	3.0E-05	1.8E-05	1.0E-05	6.8E-06	1.0E-06	8.2E-07	5.0E-05	3.0E-07	3.1E-07	5.1E-05
5.1E-05	2.6E-05	5.0E-05	1.1E-05	5.6E-06	1.4E-05	6.1E-07	8.7E-07	4.0E-06	3.7E-07	3.9E-07	3.9E-06
4.9E-05	7.8E-05	3.0E-05	1.1E-05	1.7E-05	1.0E-05	6.1E-07	6.8E-07	1.2E-05	3.9E-07	4.0E-07	1.2E-05
4.9E-05	3.8E-05	3.6E-05	1.0E-05	7.9E-06	1.1E-05	7.5E-07	8.0E-07	4.7E-06	4.3E-07	2.9E-07	4.8E-06
4.4E-05	3.4E-05	2.7E-05	9.3E-06	7.6E-06	7.9E-06	7.1E-07	7.1E-07	1.1E-05	4.0E-07	3.4E-07	1.1E-05
3.5E-05	4.1E-05	2.9E-05	7.4E-06	9.2E-06	8.2E-06	8.8E-07	7.6E-07	8.0E-06	4.3E-07	4.7E-07	8.1E-06
3.7E-05	6.3E-05	4.2E-05	7.4E-06	1.5E-05	1.2E-05	7.6E-07	1.1E-06	4.0E-06	4.4E-07	6.0E-07	4.3E-06
4.3E-05	3.5E-05	3.8E-05	9.0E-06	8.1E-06	7.7E-06	8.2E-07	1.1E-06	7.1E-05	3.8E-07	5.1E-07	7.4E-05
4.8E-05	4.5E-05	9.9E-05	1.0E-05	1.1E-05	2.4E-05	9.9E-07	9.0E-07	1.9E-05	2.9E-07	4.4E-07	2.0E-05
3.5E-05	4.6E-05	2.4E-05	7.6E-06	9.6E-06	6.0E-06	8.1E-07	7.2E-07	2.7E-05	5.0E-07	3.0E-07	2.6E-05
5.0E-05	2.3E-05	6.9E-05	1.1E-05	4.9E-06	1.8E-05	6.4E-07	7.0E-07	8.2E-06	3.9E-07	3.6E-07	8.3E-06
1.0E-04	2.3E-05	3.4E-05	2.1E-05	4.9E-06	1.1E-05	1.3E-06	8.1E-07	7.0E-06	5.4E-07	3.6E-07	6.6E-06
4.7E-05	2.5E-05	3.9E-05	9.4E-06	5.3E-06	1.1E-05	1.6E-06	8.0E-07	3.4E-06	4.2E-07	3.1E-07	3.2E-06
4.7E-05	2.5E-05	2.6E-05	9.8E-06	5.5E-06	7.2E-06	9.1E-07	6.9E-07	1.2E-05	3.4E-07	3.5E-07	1.1E-05
1.2E-04	5.6E-05	4.2E-05	2.5E-05	1.2E-05	1.1E-05	7.3E-07	7.5E-07	2.3E-06	3.2E-07	4.4E-07	2.1E-06
2.5E-05	6.2E-05	2.5E-05	5.3E-06	1.3E-05	6.2E-06	6.2E-07	6.5E-07	3.7E-05	3.1E-07	4.7E-07	3.8E-05
3.7E-05	4.9E-05	3.4E-05	8.2E-06	1.0E-05	8.6E-06	6.2E-07	6.9E-07	3.7E-06	3.3E-07	3.3E-07	4.2E-06
3.0E-05	5.6E-05	8.0E-05	6.4E-06	1.2E-05	1.8E-05	7.3E-07	6.6E-07	1.0E-05	3.9E-07	4.2E-07	1.0E-05

3.5E-05	5.1E-05	7.7E-05	7.6E-06	1.0E-05	1.7E-05	6.5E-07	1.1E-06	9.5E-06	3.9E-07	2.7E-07	9.5E-06
4.7E-05	5.1E-05	4.5E-05	9.3E-06	1.1E-05	9.5E-06	7.8E-07	5.6E-07	2.0E-06	2.9E-07	2.6E-07	1.4E-06
2.6E-05	4.1E-05		5.4E-06	8.8E-06		9.3E-07	9.2E-07		3.6E-07	4.8E-07	
4.9E-05	4.4E-05		9.7E-06	9.4E-06		1.2E-06	6.5E-07		3.4E-07	3.5E-07	
3.3E-05	5.5E-05		6.9E-06	1.2E-05		6.4E-07	8.6E-07		3.0E-07	4.6E-07	
4.3E-05	4.2E-05		8.8E-06	9.0E-06		7.8E-07	6.5E-07		2.9E-07	3.5E-07	
3.6E-05	7.1E-05		7.9E-06	1.5E-05		5.2E-07	5.9E-07		3.0E-07	2.9E-07	
2.6E-05	2.9E-05		5.5E-06	6.2E-06		6.7E-07	6.8E-07		3.2E-07	3.9E-07	
2.9E-05	7.3E-05		6.2E-06	1.6E-05		7.5E-07	9.6E-07		3.9E-07	4.1E-07	
2.5E-05	6.0E-05		5.3E-06	1.3E-05		6.9E-07	9.7E-07		2.9E-07	5.3E-07	
3.8E-05	8.4E-05		7.8E-06	1.7E-05		9.5E-07	8.4E-07		3.8E-07	5.4E-07	
3.1E-05	5.5E-05		7.0E-06	1.1E-05		8.5E-07	5.6E-07		5.0E-07	3.4E-07	
2.7E-05	4.3E-05		5.6E-06	8.9E-06		7.6E-07	9.2E-07		3.5E-07	4.8E-07	
3.0E-05	6.2E-05		6.1E-06	1.4E-05		8.7E-07	7.2E-07		4.7E-07	4.0E-07	
2.8E-05	4.1E-05		5.8E-06	8.7E-06		5.8E-07	6.4E-07		2.9E-07	3.8E-07	
2.9E-05	3.3E-05		6.4E-06	7.0E-06		6.6E-07	6.8E-07		4.6E-07	3.0E-07	
4.3E-05	5.1E-05		8.5E-06	1.0E-05		7.4E-07	1.2E-06		2.8E-07	3.5E-07	
5.9E-05	5.1E-05		1.2E-05	1.0E-05		6.1E-07	9.4E-07		2.8E-07	4.8E-07	
5.3E-05	3.4E-05		1.1E-05	6.9E-06		7.1E-07	1.4E-06		4.5E-07	4.9E-07	
5.5E-05	4.8E-05		1.1E-05	9.9E-06		6.6E-07	9.2E-07		4.0E-07	4.7E-07	
5.6E-05	2.8E-05		1.2E-05	5.8E-06		8.5E-07	8.8E-07		3.4E-07	4.2E-07	
6.4E-05	4.5E-05		1.3E-05	9.7E-06		6.9E-07	1.4E-06		3.0E-07	4.5E-07	
3.4E-05	2.4E-05		7.2E-06	5.3E-06		7.2E-07	9.7E-07		3.3E-07	5.4E-07	
4.1E-05	2.4E-05		8.5E-06	5.2E-06		6.7E-07	6.2E-07		2.9E-07	3.2E-07	
4.9E-05	2.5E-05		1.0E-05	5.2E-06		6.9E-07	8.6E-07		3.5E-07	3.1E-07	
2.9E-05			6.3E-06			6.6E-07			3.5E-07		
3.3E-05			7.3E-06			7.3E-07			4.2E-07		
5.5E-05			1.2E-05			7.2E-07			3.1E-07		
5.8E-05			1.1E-05			5.8E-07			3.3E-07		
4.1E-05			8.0E-06			1.0E-06			2.8E-07		
4.4E-05			9.1E-06			5.6E-07			2.9E-07		
3.0E-05			6.1E-06			9.3E-07			5.1E-07		
4.1E-05			8.9E-06			7.3E-07			4.3E-07		
3.5E-05			7.1E-06			7.6E-07			4.5E-07		
4.3E-05			8.9E-06			6.8E-07			2.9E-07		
3.2E-05			6.9E-06			8.0E-07			4.5E-07		
4.0E-05			8.5E-06			8.0E-07			5.0E-07		
6.3E-05			1.3E-05			6.3E-07			3.6E-07		
4.5E-05			9.6E-06			6.9E-07			4.5E-07		
4.6E-05			9.3E-06			1.1E-06			2.7E-07		
5.1E-05			1.0E-05			6.7E-07			2.8E-07		
6.7E-05			1.5E-05			8.2E-07			3.6E-07		
4.2E-05			8.7E-06			6.0E-07			3.6E-07		

6.3E-05			1.4E-05			9.9E-07			5.2E-07		
4.8E-05			9.6E-06			7.1E-07			3.8E-07		
4.7E-05			9.5E-06			1.1E-06			2.6E-07		
4.1E-05			8.4E-06			8.5E-07			3.9E-07		
5.6E-05			1.1E-05			7.2E-07			2.8E-07		
6.5E-05			1.4E-05			8.4E-07			3.3E-07		
6.8E-05			1.5E-05			1.1E-06			3.9E-07		
4.1E-05			8.5E-06			8.8E-07			3.8E-07		
2.5E-05			5.1E-06			6.9E-07			3.4E-07		
3.2E-05			6.4E-06			1.0E-06			3.0E-07		

Table S9.1 Match QC values training set, test set (containing samples that underwent the same preparation as the training set) and samples that underwent a single centrifugation step during plasma collection. Match QC values exceeding the training set threshold are colored pink.

	uncorrected			chi corrected			LOESS GC corrected			LOESS GC and chi corrected		
	training set	test set	single centrifugation samples	training set	test set	single centrifugation samples	training set	test set	single centrifugation samples	training set	test set	single centrifugation samples
min	2.5E-05	2.3E-05	2.4E-05	5.1E-06	4.9E-06	5.9E-06	5.2E-07	5.6E-07	2.0E-06	2.6E-07	2.6E-07	1.4E-06
max	1.2E-04	8.9E-05	1.0E-04	2.5E-05	1.7E-05	2.5E-05	2.4E-06	1.4E-06	7.6E-05	1.1E-06	6.0E-07	8.0E-05
average	4.5E-05	4.5E-05	4.6E-05	9.5E-06	9.5E-06	1.2E-05	8.2E-07	8.3E-07	1.6E-05	3.9E-07	3.9E-07	1.6E-05
stdev	1.7E-05			3.4E-06			2.6E-07			1.2E-07		
Threshold: average +3sd	9.5E-05			2.0E-05			1.6E-06			7.5E-07		
Average above threshold		No	No		No	No		No	Yes		No	Yes

Table S9.2 Match QC statistics training set, test set (containing samples that underwent the same preparation as the training set) and samples that have underwent a single centrifugation step during plasma collection.

Figure S8.1 (on next page): Z-scores training set, test set (containing samples that underwent the same preparation as the training set) and samples that underwent a single centrifugation step during plasma collection. (a) chromosome 13; **(b)** chromosome 18; **(c)** chromosome 21; **(1)** uncorrected; **(2)** χ^2VR corrected; **(3)** LOESS GC corrected; **(4)** LOESS GC and χ^2VR corrected.

a1

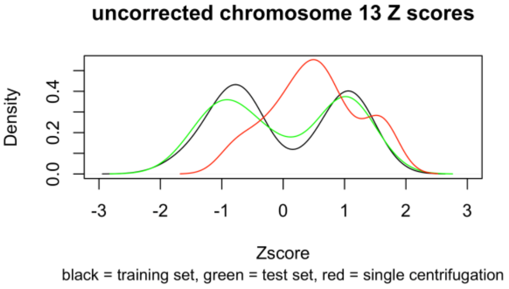

a2

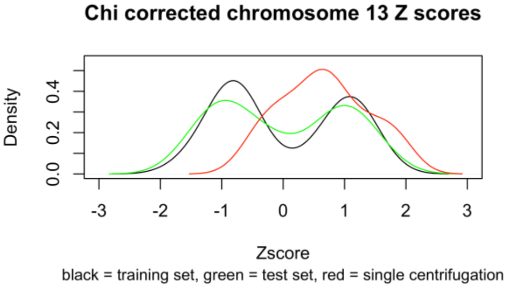

a3

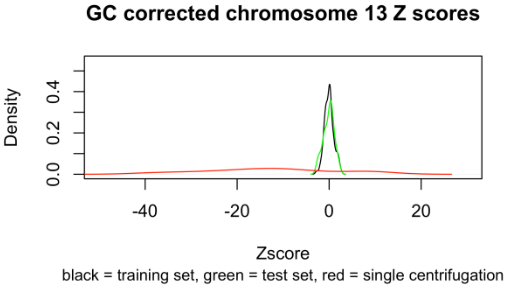

a4

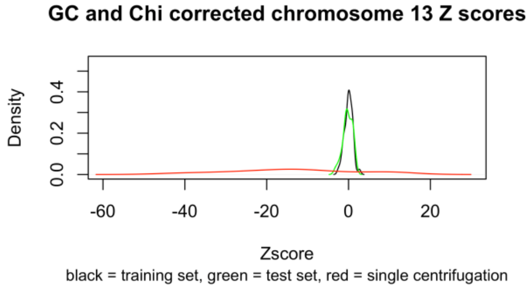

b1

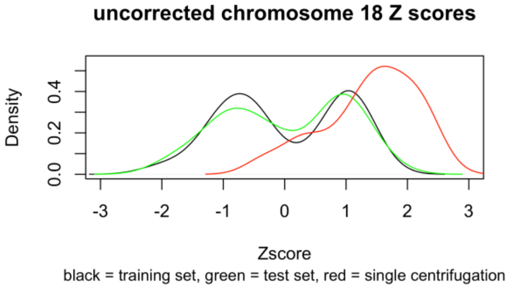

b2

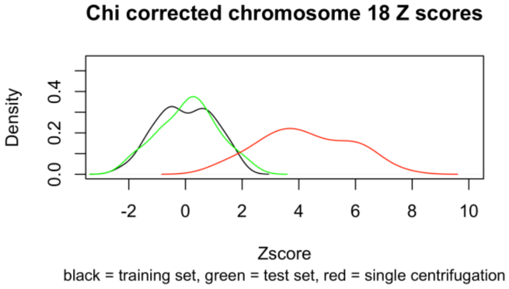

b3

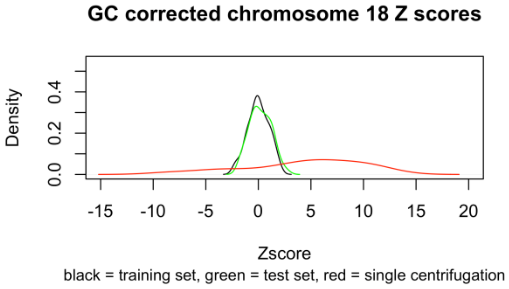

b4

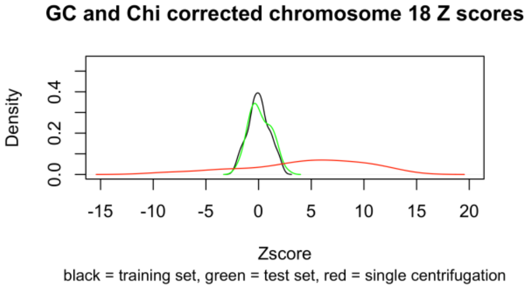

c1

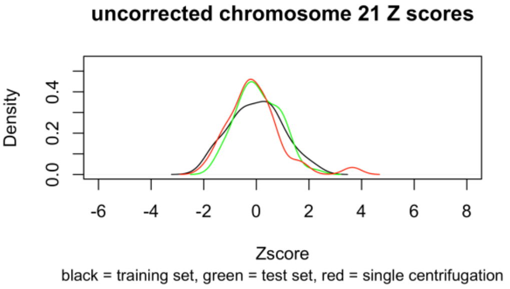

c2

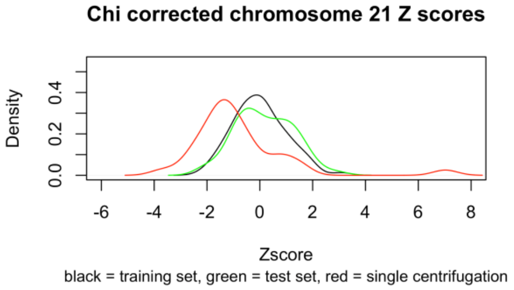

c3

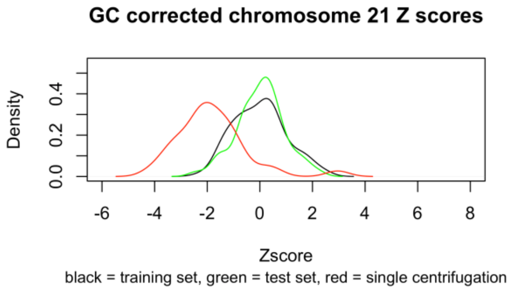

c4

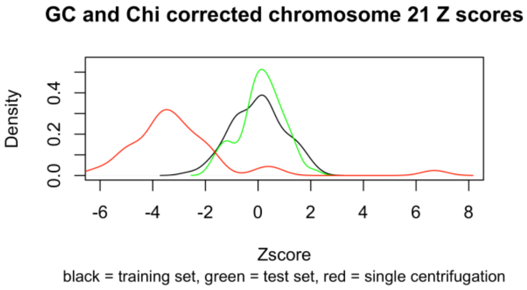

Supplement: Supplementary file 1 — Supplementary material [file 41598_2017_2031_MOESM1_ESM.pdf]
